# Supplementary material for: Automated design of multi-target ligands by generative deep learning
Source: Nat Commun. 2024 Sep 11;15:7946. doi: 10.1038/s41467-024-52060-8 (PMC11390726; doi:10.1038/s41467-024-52060-8)
Supplement: Supplementary file 1 — Supplementary Information [file 41467_2024_52060_MOESM1_ESM.pdf]

– Supplementary Information –

Automated design of multi-target ligands by generative deep learning

Laura Isigkeit[1], Tim Hörmann[2], Espen Schallmayer[1], Katharina Scholz[2], Felix F. Lillich[1,3], Johanna H. M. Ehrler[1], Benedikt Hufnagel[1], Jasmin Büchner[1], Julian A. Marschner[2], Jörg Pabel[2], Ewgenij Proschak[1,3], Daniel Merk[1,2]*

[1] Goethe University Frankfurt, Institute of Pharmaceutical Chemistry, 60438 Frankfurt, Germany

[2] Ludwig-Maximilians-Universität München, Department of Pharmacy, 81377 Munich, Germany

[3] Fraunhofer Institute for Translational Medicine and Pharmacology ITMP, 60596 Frankfurt, Germany

* daniel.merk@cup.lmu.de

Table of Contents

Supplementary Figures and Tables 2

Supplementary Methods 20

Supplementary References 51

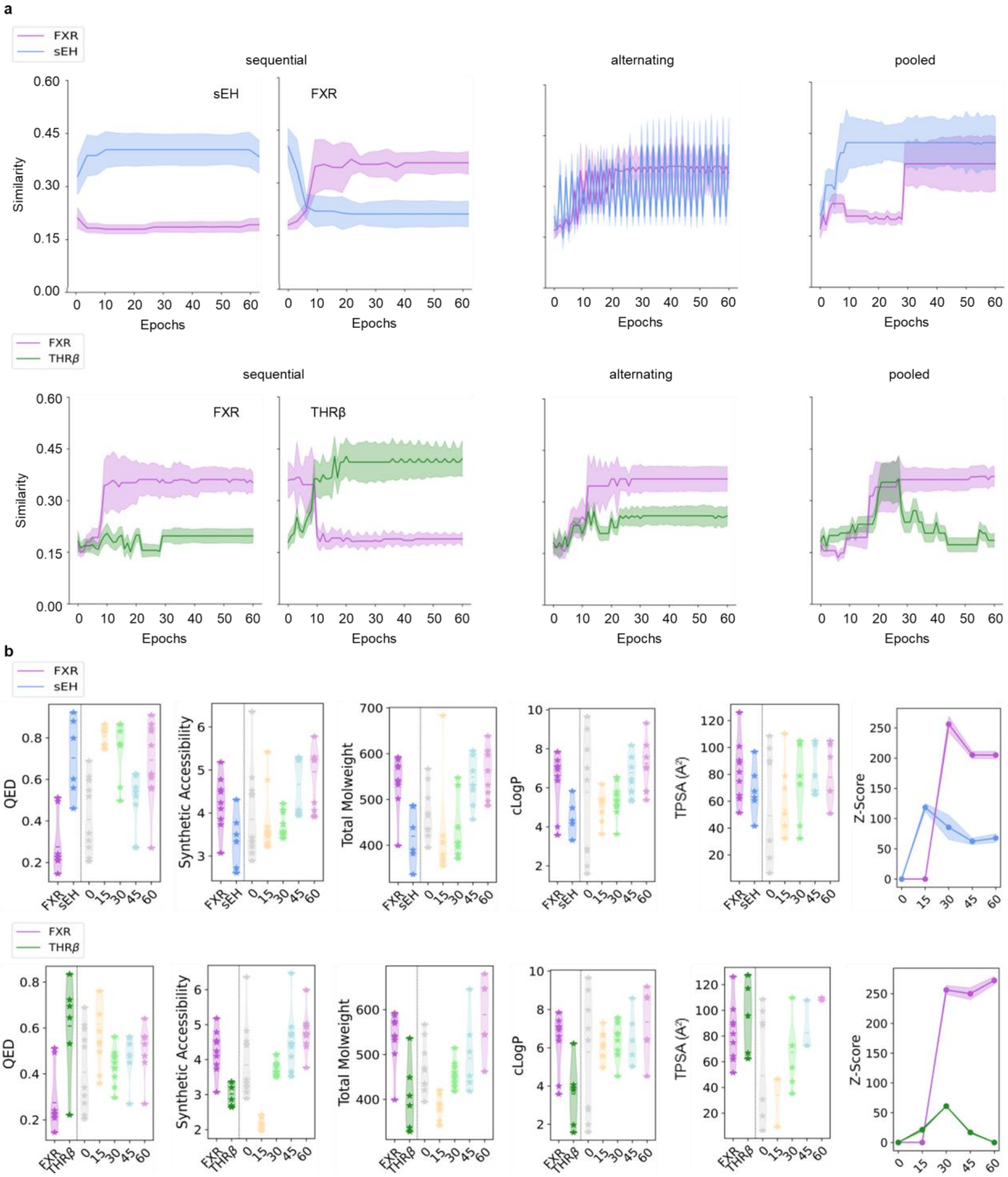

Suppl. Fig. 1. Fine-Tuning chemical language model (CLM) for multi-target design for target pairs FXR/sEH and FXR/THRβ. (a) Effects of different CLM fine-tuning strategies on the similarity of beam search designs (width 50) to the fine-tuning molecules (FXR/sEH and FXR/THRβ). Fine-tuning with pooled template sets was superior to sequential and alternating fine-tuning strategies in terms of design similarity to both fine-tuning collections. Graphs show the max. Tanimoto similarity similarity ± SD (of max. Tanimoto similarity) computed on Morgan fingerprints of the beam search designs per epoch to the fine-tuning molecules. For each epoch beam search designs (width 50) were designed and only SMILES that were valid were analyzed. (b) Quantitative estimation of drug-likeness (QED) scores[1], synthetic accessibility score[2], basic molecular features and target prediction (Z-Scores using Similarity Ensemble Approach (SEA)[3]) of CLM designs over the fine-tuning procedure (epochs 0, 15, 30, 45, 60). For each epoch beam search designs (width 50) were designed and only SMILES that were valid were analyzed. Source data are provided as a Source Data file.

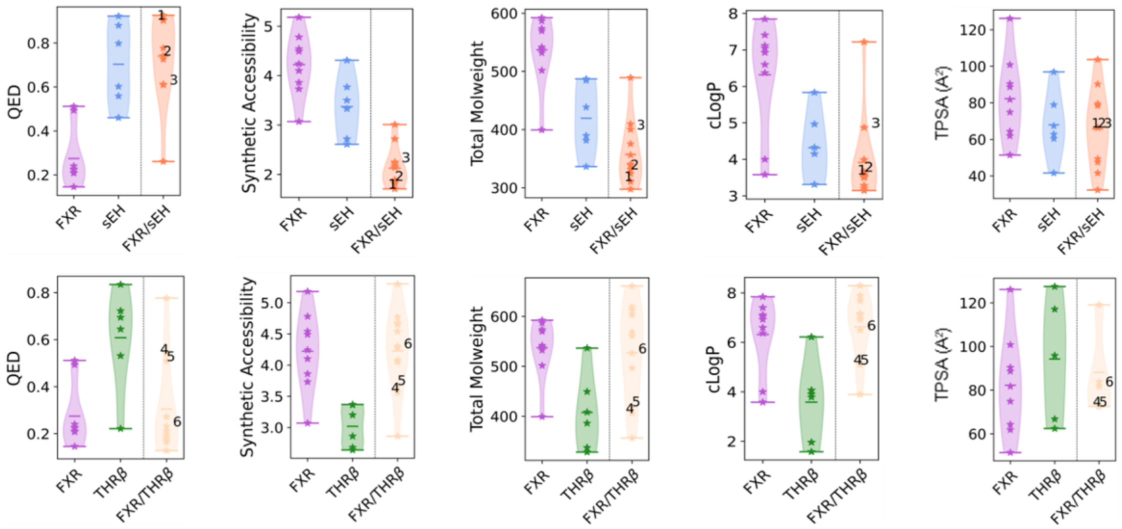

Suppl. Fig. 2. Quantitative estimation of drug-likeness (QED) scores[1], synthetic accessibility score[2] and basic molecular features of the chemical language model (CLM) designs were favorable and resembled the fine-tuning molecules. Numbers of analyzed ligands for FXR: 9, THRβ: 6, sEH: 6, FXR/THRβ and FXR/sEH: 12. Source data are provided as a Source Data file.

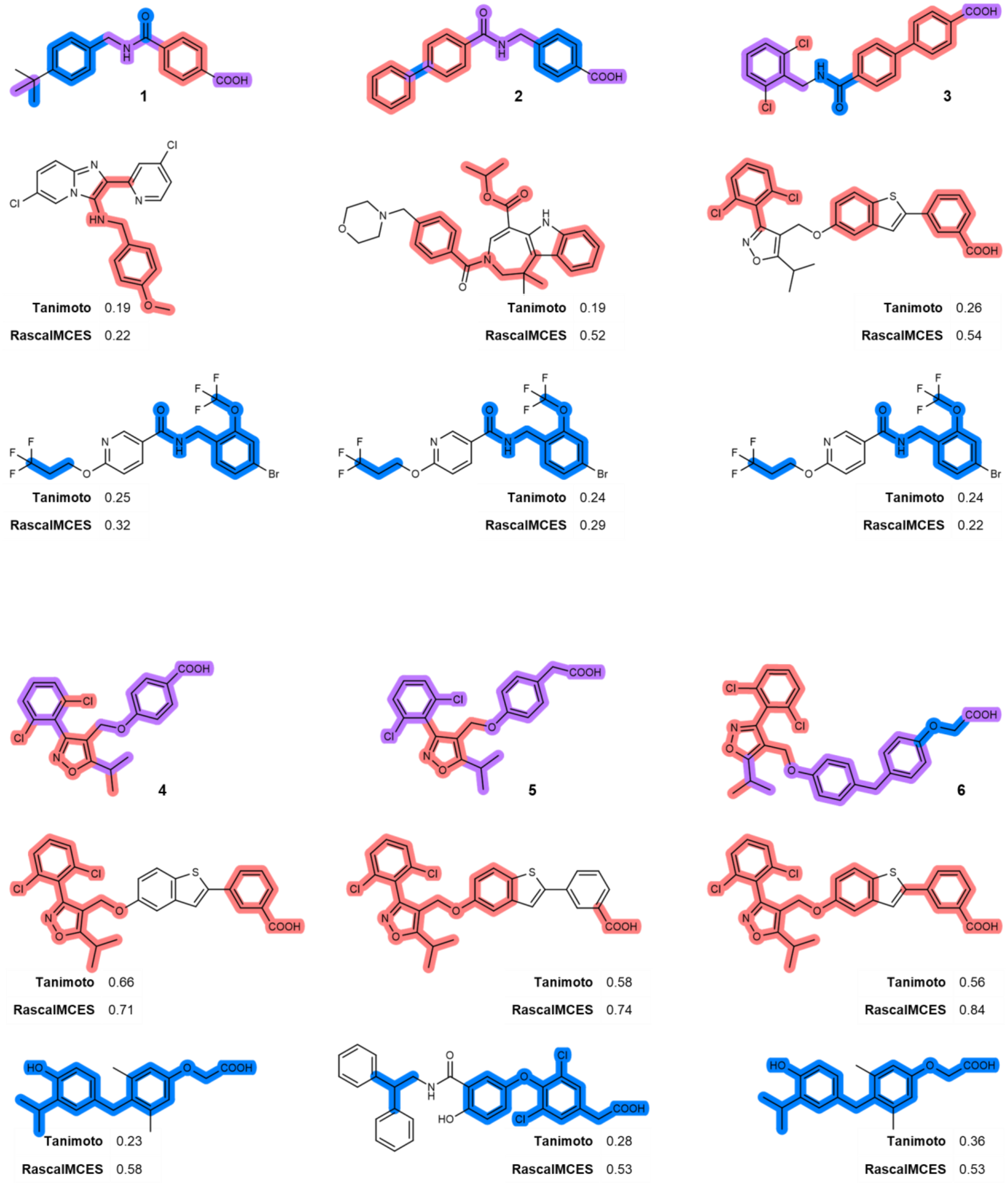

Suppl. Fig. 3. Comparison of designed dual modulators with the most similar fine-tuning templates by Tanimoto similarity computed on Morgan fingerprints and Rapid Similarity Calculation of Maximum Common Edge Subgraph (RascalMCES)[4]. The highlighted substructures represent the extracted RascalMCES between the most similar fine-tuning templates and the designed dual modulators (**1**-**3**: red – FXR, blue – sEH, violet – both; **4** - **6**: red – FXR, blue – THRβ, violet – both).

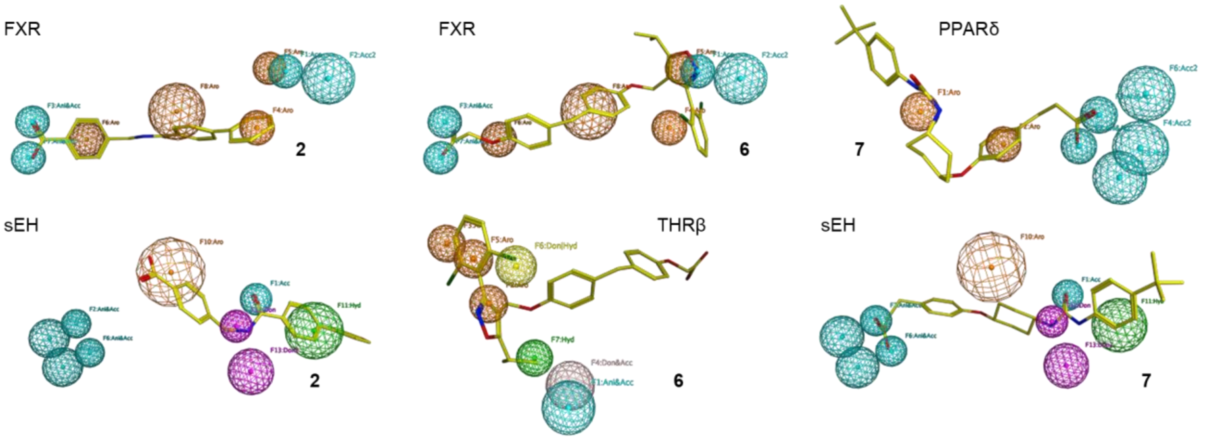

Suppl. Fig. 4. Pharmacophore models for evaluation of dual ligand design performance (cf. Fig. 6d). The models were developed in MOE based on ligand-bound co-crystal structures of FXR (Protein data bank (pdb) IDs: 6a60[5], 3dcu[6], 3fli[7]), THRβ (pdb IDs: 1nax[8], 1r6g[9], 6kkb[10]), PPARδ (pdb IDs: 5y7x, 3tkm[11], 5u46[12]), and sEH (pdb IDs: 5ali[13], 3ant[14], 3wke[15]). Models are shown with representative dual ligands developed by the chemical language model (CLM). Fractions of designs from the baseline CLM, the target pair focused CLMs and a CLM fine-tuned with ligands for FXR, THRβ, PPARδ and sEH matching the pharmacophore models are shown in Fig. 6d.

Suppl. Tab. 1. Fine-tuning sets for PPARδ, FXR, THRβ, sEH, AT_1 and FFAR1 with their IC_{50}/EC_{50} values (from [16]) used for chemical language model (CLM) fine-tuning.

Target	Chemical structure	SMILES	potency on target (EC_{50} for PPARδ, FXR, THRβ, FFAR1; IC_{50} for sEH, AT_1)
PPARδ	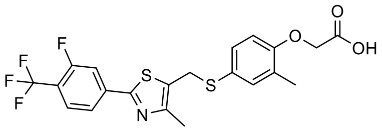	OC(=O)COc1ccc(cc1C)SCc1sc(nc1C)c1ccc(c(c1)F)C(F)(F)F	13 nM
PPARδ	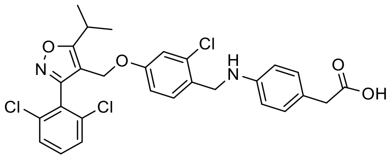	OC(=O)Cc1ccc(cc1)NCc1ccc(cc1Cl)OCc1c(onc1c1c(Cl)cccc1Cl)C(C)C	2 µM
PPARδ	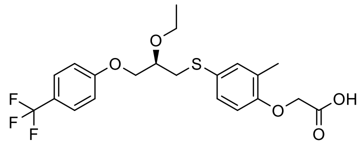	CCO[C@H](COc1ccc(cc1)C(F)(F)F)CSc1ccc(c(c1)C)OCC(=O)O	2 nM
PPARδ	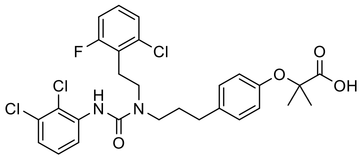	O=C(N(CCc1c(F)cccc1Cl)CCCc1ccc(cc1)OC(C(=O)O)(C)C)Nc1cccc(c1Cl)Cl	190 nM
PPARδ	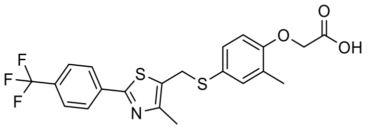	OC(=O)COc1ccc(cc1C)SCc1sc(nc1C)c1ccc(cc1)C(F)(F)F	1 nM
FXR	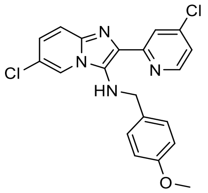	COc1ccc(CNc2c(-c3cc(Cl)ccn3)nc3ccc(Cl)cn23)cc1	0.3 nM
FXR	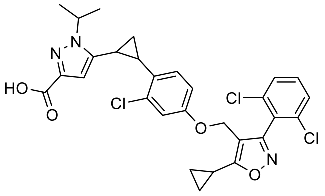	CC(C)n1nc(C(=O)O)cc1C1CC1c1ccc(OCc2c(-c3c(Cl)cccc3Cl)noc2C2CC2)cc1Cl	4 nM
FXR	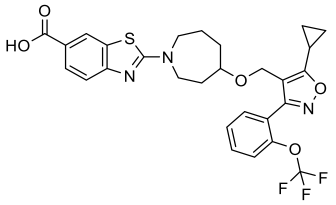	O=C(O)c1ccc2nc(N3CCCC(OCc4c(-c5ccccc5OC(F)(F)F)noc4C4CC4)CC3)sc2c1	7 nM
FXR	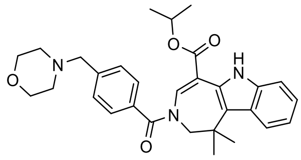	CC(C)OC(=O)C1=CN(C(=O)c2ccc(CN3CCOCC3)cc2)CC(C)(C)c2c1[nH]c1ccccc21	23 nM
FXR	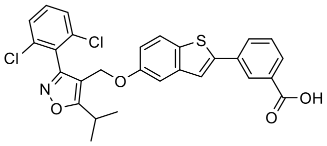	CC(C)c1onc(-c2c(Cl)cccc2Cl)c1COc1ccc2sc(-c3cccc(C(=O)O)c3)cc2c1	32 nM

Target	Chemical structure	SMILES	potency on target (EC50 for PPARδ, FXR, THRβ, FFAR1; IC50 for sEH, AT1)
FXR	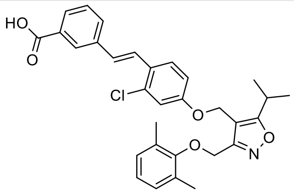	Cc1cccc(C)c1OCc1noc(C(C)C)c1COc1ccc(C=Cc2cccc(C(=O)O)c2)c(Cl)c1	56 nM
FXR	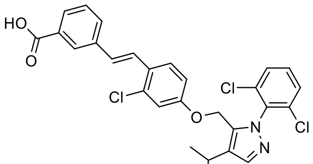	CC(C)c1cnn(-c2c(Cl)cccc2Cl)c1COc1ccc(C=Cc2cccc(C(=O)O)c2)c(Cl)c1	20 nM
FXR	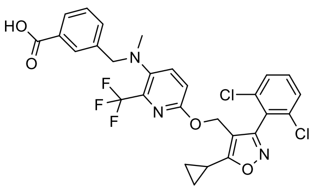	CN(Cc1cccc(C(=O)O)c1)c1ccc(OCc2c(-c3c(Cl)cccc3Cl)noc2C2CC2)nc1C(F)(F)F	9 nM
FXR	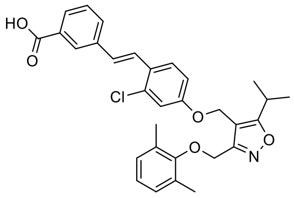	Cc1cccc(C)c1OCc1noc(C(C)C)c1COc1ccc(C=Cc2cccc(C(=O)O)c2)c(Cl)c1	56 nM
THRβ	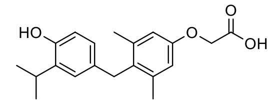	Cc1cc(OCC(=O)O)cc(C)c1Cc1ccc(O)c(C(C)C)c1	7 nM
THRβ	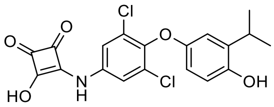	CC(C)c1cc(Oc2c(Cl)cc(Nc3c(O)c(=O)c3=O)cc2Cl)ccc1O	70 nM
THRβ	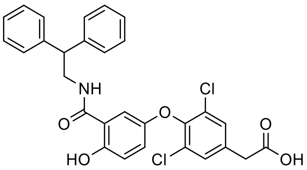	O=C(O)Cc1cc(Cl)c(Oc2ccc(O)c(C(=O)NCC(c3ccccc3)c3ccccc3)c2)c(Cl)c1	0.5 nM
THRβ	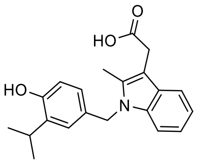	Cc1c(CC(=O)O)c2ccccc2n1Cc1ccc(O)c(C(C)C)c1	150 nM
THRβ	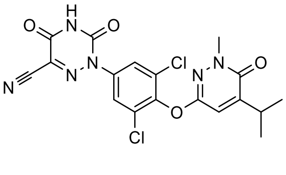	CC(C)c1cc(Oc2c(Cl)cc(-n3nc(C#N)c(=O)[nH]c3=O)cc2Cl)nn(C)c1=O	40 nM
THRβ	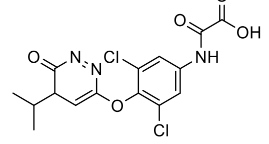	CC(C)c1cc(Oc2c(Cl)cc(NC(=O)C(=O)O)cc2Cl)n[nH]c1=O	120 nM

Target	Chemical structure	SMILES	potency on target (EC_{50} for PPARδ, FXR, THRβ, FFAR1; IC_{50} for sEH, AT_1)
sEH	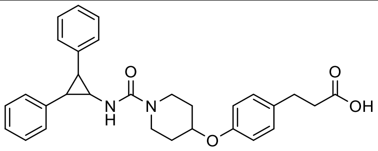	O=C(O)CCc1ccc(OC2CCN(C(=O)NC3C(c4ccccc4)C3c3ccccc3)CC2)cc1	2 nM
sEH	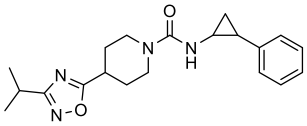	CC(C)c1noc(C2CCN(C(=O)NC3CC3c3ccccc3)CC2)n1	6 nM
sEH	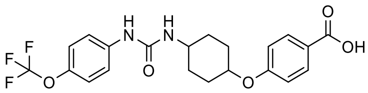	O=C(Nc1ccc(OC(F)(F)F)cc1)NC1CCC(Oc2ccc(C(=O)O)cc2)CC1	0.4 nM
sEH	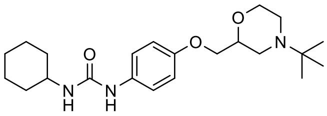	CC(C)(C)N1CCOC(COc2ccc(NC(=O)NC3CCCCC3)cc2)C1	77 nM
sEH	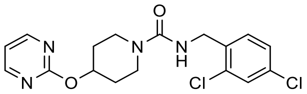	O=C(NCc1ccc(Cl)cc1Cl)N1CCC(Oc2ncccn2)CC1	15 nM
sEH	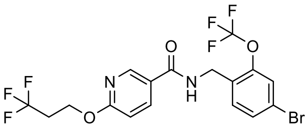	O=C(NCc1ccc(Br)cc1OC(F)(F)F)c1ccc(OCCC(F)(F)F)nc1	12 nM
AT_1	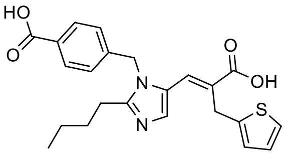	CCCCc1ncc(/C=C(\Cc2cccs2)/C(O)=O)n1Cc(cc1)ccc1C(O)=O	2 nM
AT_1	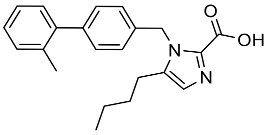	CCCCc1cnc(C(O)=O)n1Cc(cc1)ccc1-c1c(C)cccc1	3 nM
AT_1	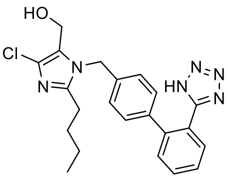	CCCCc1nc(Cl)c(CO)n1Cc(cc1)ccc1-c(cccc1)c1-c1nnn[nH]1	0.3 nM
AT_1	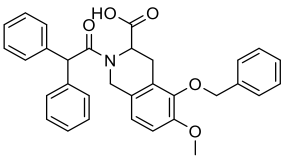	COc(ccc(C1)c2CC(C(O)=O)N1C(C(c1ccccc1)c1ccccc1)=O)c2OCc1ccccc1	1 μM

Target	Chemical structure	SMILES	potency on target (EC$_{50}$ for PPARδ, FXR, THRβ, FFAR1; IC$_{50}$ for sEH, AT$_1$)
AT$_1$	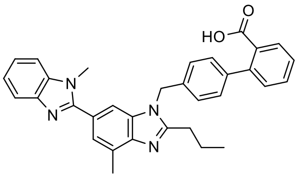	CCCc1nc(c(C)cc(-c2nc(cccc3)c3n2C)c2)c2n1Cc(cc1)ccc1-c(cccc1)c1C(O)=O	3 nM
AT$_1$	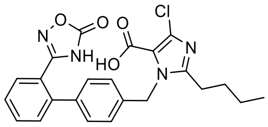	CCCCc1nc(Cl)c(C(O)=O)n1Cc(cc1)ccc1-c(cccc1)c1C(N1)=NOC1=O	2 nM
FFAR1	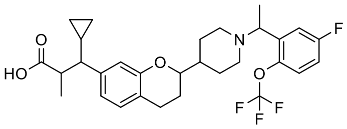	CC(C(=O)O)C(c1ccc2c(c1)OC(C1CCN(C(C)c3cc(F)ccc3OC(F)(F)F)CC1)CC2)C1CC1	2 nM
FFAR1	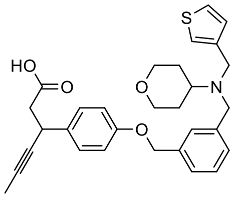	CC#CC(CC(=O)O)c1ccc(OCc2cccc(CN(Cc3ccsc3)C3CCOCC3)c2)cc1	8 nM
FFAR1	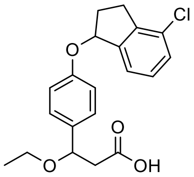	CCOC(CC(=O)O)c1ccc(OC2CCc3c(Cl)cccc32)cc1	5 nM
FFAR1	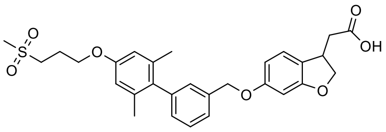	Cc1cc(OCCCS(C)(=O)=O)cc(C)c1-c1cccc(COc2ccc3c(c2)OCC3CC(=O)O)c1	370 nM
FFAR1	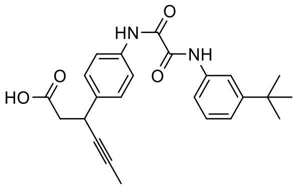	CC#CC(CC(=O)O)c1ccc(NC(=O)C(=O)Nc2cccc(C(C)(C)C)c2)cc1	4 nM
FFAR1	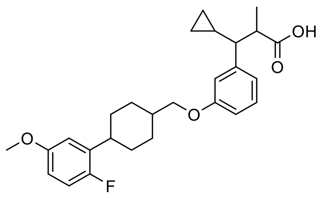	COc1ccc(F)c(C2CCC(COc3cccc(C(C4CC4)C(C)C(=O)O)c3)CC2)c1	2 nM

Suppl. Tab. 2. Novelty and property analysis of beam search designs from selected epochs of fine-tuning and the top 12 selected designs.

Epoch		0	15	30	45	60	Top 12
Novelty (%)[17] compared with ChEMBL32	FXR / sEH	53	86	80	94	94	83
	FXR / THRβ	53	100	100	100	100	92
	PPARδ / sEH	53	100	95	100	100	92
	AT_1 / FFAR1	53	100	100	100	100	92
	AT_1 / sEH	53	70	80	86	100	75
	FFAR1 / sEH	91	86	90	97	89	100
Scaffold diversity (% – ratio of unique scaffolds)	FXR / sEH	19	14	21	26	26	42
	FXR / THRβ	38	4	8	20	27	42
	PPARδ / sEH	53	78	79	92	94	83
	AT_1 / FFAR1	53	100	88	85	100	75
	AT_1 / sEH	53	50	73	93	67	75
	FFAR1 / sEH	53	55	55	50	46	75
Tanimoto-Similarity between designs of each target combination	FXR / sEH	0.23 ± 0.05	0.32 ± 0.03	0.30 ± 0.03	0.29 ± 0.03	0.31 ± 0.04	0.50 ± 0.06
	FXR / THRβ	0.23 ± 0.05	0.18 ± 0.04	0.30 ± 0.03	0.30 ± 0.03	0.34 ± 0.03	0.69 ± 0.09
	PPARδ / sEH	0.13 ± 0.05	0.24 ± 0.07	0.26 ± 0.04	0.24 ± 0.05	0.23 ± 0.03	0.28 ± 0.08
	AT_1 / FFAR1	0.13 ± 0.05	0.15 ± 0.02	0.18 ± 0.02	0.16 ± 0.02	0.16 ± 0.01	0.26 ± 0.02
	AT_1 / sEH	0.13 ± 0.05	0.32 ± 0.02	0.26 ± 0.07	0.24 ± 0.06	0.25 ± 0.05	0.30 ± 0.06
	FFAR1 / sEH	0.26 ± 0.03	0.30 ± 0.06	0.28 ± 0.06	0.25 ± 0.05	0.22 ± 0.04	0.42 ± 0.05
Synthetic accessibility[18] (1 - easy, 10 - very difficult)	FXR / sEH	4.0 ± 0.8	3.0 ± 0.6	3.3 ± 0.4	3.5 ± 0.7	3.8 ± 0.7	2.1 ± 0.4
	FXR / THRβ	4.0 ± 0.8	2.1 ± 0.2	3.6 ± 0.2	3.8 ± 0.6	4.0 ± 0.6	4.2 ± 0.6
	PPARδ / sEH	3.8 ± 1.4	3.8 ± 0.5	3.8 ± 0.9	3.9 ± 0.6	4.0 ± 0.6	3.3 ± 0.6
	AT_1 / FFAR1	3.8 ± 1.4	3.9 ± 0.9	3.9 ± 0.5	3.9 ± 0.4	3.3 ± 0.5	3.8 ± 0.3
	AT_1 / sEH	3.8 ± 1.4	2.9 ± 0.6	3.5 ± 0.6	3.3 ± 0.7	3.6 ± 0.8	3.1 ± 0.6
	FFAR1 / sEH	2.9 ± 0.6	2.9± 0.6	2.9 ± 0.8	3.2 ± 0.9	3.5 ± 0.9	3.3 ± 0.3
Quantitative estimate of drug-likeness[1] (0.0 – no drug-likeness, 1.0 – best drug-likeness)	FXR / sEH	0.56 ± 0.16	0.78 ± 0.13	0.73 ± 0.16	0.65 ± 0.21	0.58 ± 0.23	0.74 ± 0.19
	FXR / THRβ	0.56 ± 0.16	0.59 ± 0.11	0.49 ± 0.09	0.50 ± 0.10	0.46 ± 0.15	0.30 ± 0.19
	PPARδ / sEH	0.50 ± 0.20	0.58 ± 0.20	0.57 ± 0.17	0.49 ± 0.13	0.52 ± 0.15	0.57 ± 0.17
	AT_1 / FFAR1	0.50 ± 0.20	0.50 ± 0.26	0.42 ± 0.09	0.57 ± 0.14	0.58 ± 0.12	0.49 ± 0.08
	AT_1 / sEH	0.50 ± 0.20	0.65 ± 0.10	0.67 ± 0.17	0.65 ± 0.20	0.66 ± 0.16	0.64 ± 0.16
	FFAR1 / sEH	0.67 ± 0.20	0.73 ± 0.13	0.71 ± 0.17	0.69 ± 0.19	0.65 ± 0.21	0.71 ± 0.14

Suppl. Tab. 3. Validity, uniqueness, and novelty of designs (5000) from selected epochs (40-45 (FXR/sEH), 17-21 (FXR/THRβ), 51-55 (PPARδ/sEH), 34-38 (AT_1/FFAR1), 41-42 (AT_1/sEH), 7-10 (FFAR1/sEH)) with different sampling temperatures (0.2, 0.7).

	Validity (%)	Uniqueness (% - ratio of unique molecules)	Novelty (%)[17] compared with ChEMBL32
FXR / sEH (0.7)	58.7	98.7	99.9
FXR / THRβ (0.7)	63.9	95.9	99.9
PPARδ / sEH (0.2)	93.2	4.9	87.9
AT_1 / FFAR1 (0.2)	65.8	9.5	96.8
AT_1 / sEH (0.2)	61.3	3.7	90.5
FFAR1 / sEH (0.7)	91.3	96.2	99.5

Suppl. Tab. 4. Top 12 molecules generated by the chemical language model (CLM) per target pair. 5000 molecules were sampled from selected epochs and 12 molecules were selected for further evaluation based on their abundance (sampling frequency over the selected epochs) and mean similarity to their corresponding fine-tuning sets. IDs refer to the compounds selected for experimental validation.

designs for FXR/sEH (top 12 from epochs 40-45)	SMILES	Abun-dance (rank)	Similarity FXR / sEH	Sampling Temperature	ID
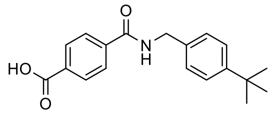	CC(C)(C)c1ccc(CNC(=O)c2ccc(C(=O)O)cc2)cc1	1 (1485)	0.16 / 0.20	0.7	**1**
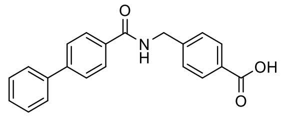	O=C(O)c1ccc(CNC(=O)c2ccc(-c3ccccc3)cc2)cc1	1 (1485)	0.17 / 0.20	0.7	**2**
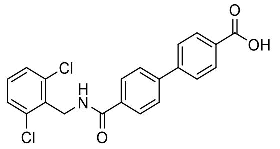	O=C(O)c1ccc(-c2ccc(C(=O)NCc3c(Cl)cccc3Cl)cc2)cc1	2 (792)	0.20 / 0.20	0.7	**3**
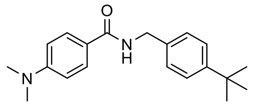	CN(C)c1ccc(C(=O)NCc2ccc(C(C)(C)C)cc2)cc1	1 (1485)	0.15 / 0.16	0.7	
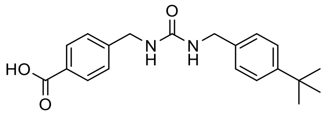	CC(C)(C)c1ccc(CNC(=O)NCc2ccc(C(=O)O)cc2)cc1	1 (1485)	0.16 / 0.20	0.7	
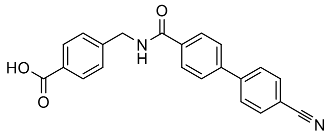	N#Cc1ccc(-c2ccc(C(=O)NCc3ccc(C(=O)O)cc3)cc2)cc1	1 (1485)	0.14 / 0.18	0.7	
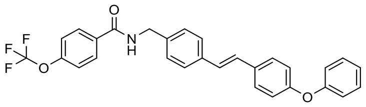	O=C(NCc1ccc(C=Cc2ccc(Oc3ccccc3)cc2)cc1)c1ccc(OC(F)(F)F)cc1	1 (1485)	0.18 / 0.23	0.7	
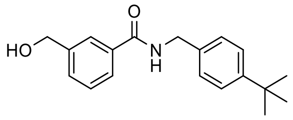	CC(C)(C)c1ccc(CNC(=O)c2cccc(CO)c2)cc1	1 (1485)	0.17 / 0.16	0.7	
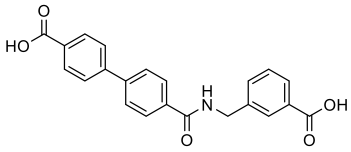	O=C(O)c1ccc(-c2ccc(C(=O)NCc3cccc(C(=O)O)c3)cc2)cc1	1 (1485)	0.20 / 0.19	0.7	
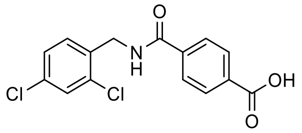	O=C(O)c1ccc(C(=O)NCc2ccc(Cl)cc2Cl)cc1	1 (1485)	0.17 / 0.24	0.7	
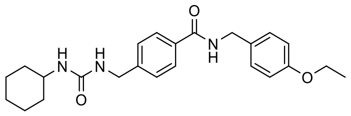	CCOc1ccc(CNC(=O)c2ccc(CNC(=O)NC3CCCCC3)cc2)cc1	1 (1485)	0.17 / 0.26	0.7	
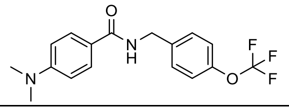	CN(C)c1ccc(C(=O)NCc2ccc(OC(F)(F)F)cc2)cc1	1 (1485)	0.18 / 0.22	0.7	

designs for FXR/THRβ (top 12 from epochs 17-21)	SMILES	Abundance (rank)	Similarity FXR / THRβ	Sampling Temperature	ID
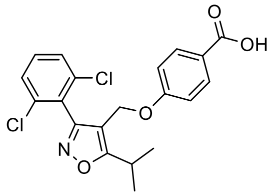	CC(C)c1onc(-c2c(Cl)cccc2Cl)c1COc1ccc(C(=O)O)cc1	15 (22)	0.34 / 0.20	0.7	**4**
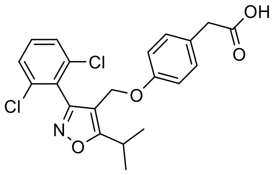	CC(C)c1onc(-c2c(Cl)cccc2Cl)c1COc1ccc(CC(=O)O)cc1	12 (28)	0.31 / 0.23	0.7	**5**
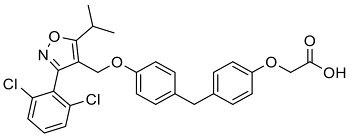	CC(C)c1onc(-c2c(Cl)cccc2Cl)c1COc1ccc(Cc2ccc(OCC(=O)O)cc2)cc1	8 (35)	0.31 / 0.22	0.7	**6**
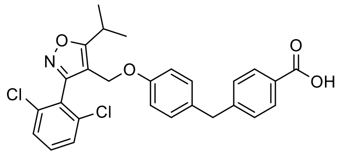	CC(C)c1onc(-c2c(Cl)cccc2Cl)c1COc1ccc(Cc2ccc(C(=O)O)cc2)cc1	3 (153)	0.34 / 0.20	0.7	
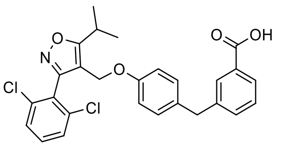	CC(C)c1onc(-c2c(Cl)cccc2Cl)c1COc1ccc(Cc2cccc(C(=O)O)c2)cc1	2 (889)	0.35 / 0.21	0.7	
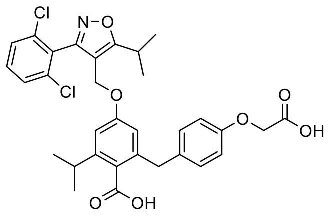	CC(C)c1cc(OCc2c(-c3c(Cl)cccc3Cl)noc2C(C)C)cc(Cc2ccc(OCC(=O)O)cc2)c1C(=O)O	2 (889)	0.30 / 0.24	0.7	
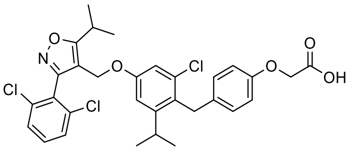	CC(C)c1cc(OCc2c(-c3c(Cl)cccc3Cl)noc2C(C)C)cc(Cl)c1Cc1ccc(OCC(=O)O)cc1	1 (1572)	0.30 / 0.25	0.7	
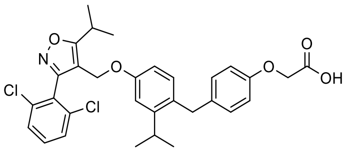	CC(C)c1cc(OCc2c(-c3c(Cl)cccc3Cl)noc2C(C)C)ccc1Cc1ccc(OCC(=O)O)cc1	1 (1572)	0.31 / 0.25	0.7	
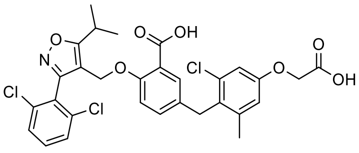	Cc1cc(OCC(=O)O)cc(Cl)c1Cc1ccc(OCc2c(-c3c(Cl)cccc3Cl)noc2C(C)C)c(C(=O)O)c1	1 (1572)	0.29 / 0.22	0.7	
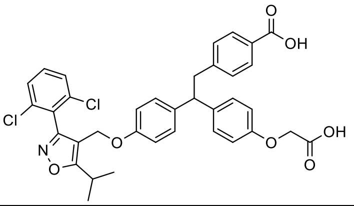	CC(C)c1onc(-c2c(Cl)cccc2Cl)c1COc1ccc(C(Cc2ccc(C(=O)O)cc2)c2ccc(OCC(=O)O)cc2)cc1	1 (1572)	0.30 / 0.21	0.7	

designs for FXR/THRβ (top 12 from epochs 17-21)	SMILES	Abun-dance (rank)	Similarity FXR / THRβ	Sampling Temperature	ID
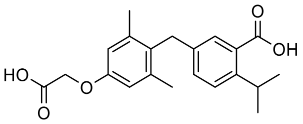	Cc1cc(OCC(=O)O)cc(C)c1Cc1ccc(C(C)C)c(C(=O)O)c1	1 (1572)	0.20 / 0.31	0.7	
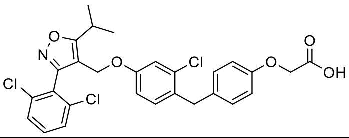	CC(C)c1onc(-c2c(Cl)cccc2Cl)c1COc1ccc(Cc2ccc(OCC(=O)O)cc2)c(Cl)c1	1 (1572)	0.31 /0.23	0.7	

designs for PPARδ/sEH (top 12 from epochs 51-55)	SMILES	Abun-dance (rank)	Similarity PPARδ / sEH	Sampling Temperature	ID
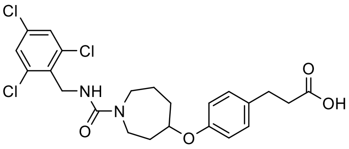	O=C(O)CCc1ccc(OC2CCCN(C(=O)NCc3c(Cl)cc(Cl)cc3Cl)CC2)cc1	2063 (1)	0.17 / 0.33	0.2	**8**
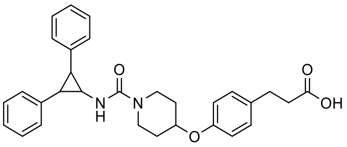	O=C(O)CCc1ccc(OC2CCN(C(=O)NC3C(c4ccccc4)C3c3ccccc3)CC2)cc1	1130 (2)	0.15 / 0.42	0.2	
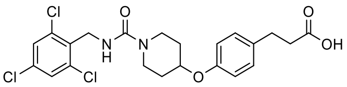	O=C(O)CCc1ccc(OC2CCN(C(=O)NCc3c(Cl)cc(Cl)cc3Cl)CC2)cc1	862 (3)	0.18 / 0.35	0.2	**9**
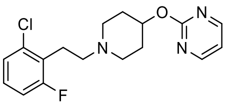	Fc1cccc(Cl)c1CCN1CCC(Oc2ncccn2)CC1	861 (4)	0.14 / 0.21	0.2	
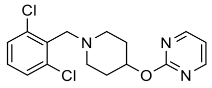	Clc1cccc(Cl)c1CN1CCC(Oc2ncccn2)CC1	816 (5)	0.12 / 0.21	0.2	
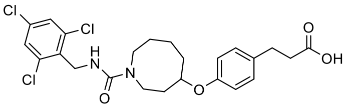	O=C(O)CCc1ccc(OC2CCCCN(C(=O)NCc3c(Cl)cc(Cl)cc3Cl)CC2)cc1	528 (6)	0.17 / 0.33	0.2	
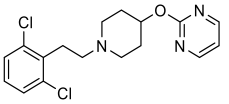	Clc1cccc(Cl)c1CCN1CCC(Oc2ncccn2)CC1	521 (7)	0.12 / 0.22	0.2	
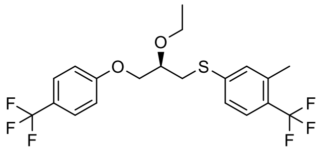	CCO[C@H](COc1ccc(C(F)(F)F)cc1)CSc1ccc(C(F)(F)F)c(C)c1	255 (8)	0.31 / 0.11	0.2	
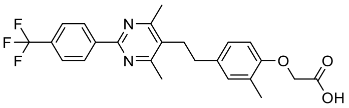	Cc1cc(CCc2c(C)nc(-c3ccc(C(F)(F)F)cc3)nc2C)ccc1OCC(=O)O	217 (9)	0.36 / 0.14	0.2	
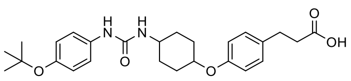	CC(C)(C)Oc1ccc(NC(=O)NC2CCC(Oc3ccc(CCC(=O)O)cc3)CC2)cc1	162 (10)	0.18 / 0.34	0.2	**7**
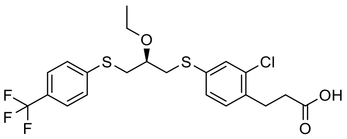	CCO[C@H](CSc1ccc(C(F)(F)F)cc1)CSc1ccc(CCC(=O)O)c(Cl)c1	144 (11)	0.30 / 0.15	0.2	
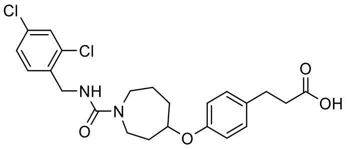	O=C(O)CCc1ccc(OC2CCCN(C(=O)NCc3ccc(Cl)cc3Cl)CC2)cc1	118 (12)	0.17 / 0.36	0.2	

designs for AT_1/sEH (top 12 from epochs 41-42)	SMILES	Abun-dance (rank)	Similarity AT_1 / sEH	Sampling Temperature	ID
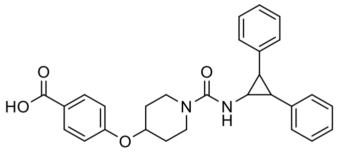	O=C(O)c1ccc(OC2CCN(C(=O)NC3C(c4ccccc4)C3c3ccccc3)CC2)cc1	906 (1)	0.14 / 0.42	0.2	
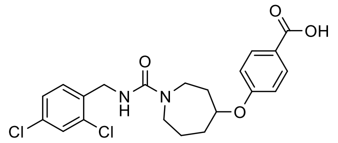	O=C(O)c1ccc(OC2CCCN(C(=O)NCc3ccc(Cl)cc3Cl)CC2)cc1	692 (2)	0.13 / 0.38	0.2	**10**
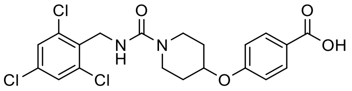	O=C(O)c1ccc(OC2CCN(C(=O)NCc3c(Cl)cc(Cl)cc3Cl)CC2)cc1	237 (3)	0.14 / 0.36	0.2	
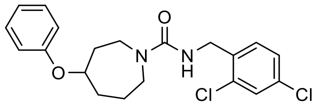	O=C(NCc1ccc(Cl)cc1Cl)N1CCCC(Oc2ccccc2)CC1	202 (4)	0.15 / 0.38	0.2	
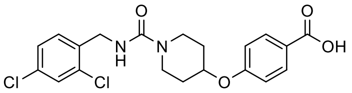	O=C(O)c1ccc(OC2CCN(C(=O)NCc3ccc(Cl)cc3Cl)CC2)cc1	132 (5)	0.13 / 0.40	0.2	
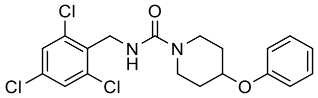	O=C(NCc1c(Cl)cc(Cl)cc1Cl)N1CCC(Oc2ccccc2)CC1	100 (6)	0.15 / 0.37	0.2	
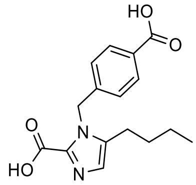	CCCCc1cnc(C(=O)O)n1Cc1ccc(C(=O)O)cc1	72 (7)	0.37 / 0.16	0.2	
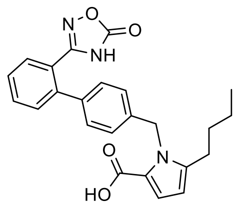	CCCCc1ccc(C(=O)O)n1Cc1ccc(-c2ccccc2-c2noc(=O)[nH]2)cc1	67 (8)	0.39 / 0.14	0.2	
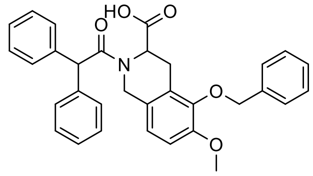	COc1ccc2c(c1OCc1ccccc1)CC(C(=O)O)N(C(=O)C(c1ccccc1)c1ccccc1)C2	60 (9)	0.29 / 0.16	0.2	
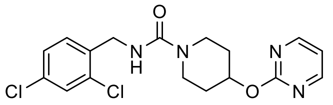	O=C(NCc1ccc(Cl)cc1Cl)N1CCC(Oc2ncccn2)CC1	44 (10)	0.14 / 0.39	0.2	
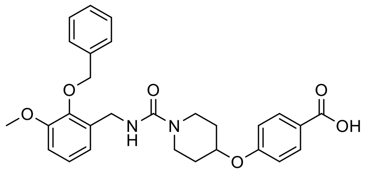	COc1cccc(CNC(=O)N2CCC(Oc3ccc(C(=O)O)cc3)CC2)c1OCc1ccccc1	38 (11)	0.19 / 0.33	0.2	
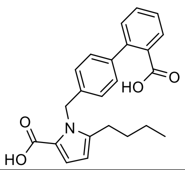	CCCCc1ccc(C(=O)O)n1Cc1ccc(-c2ccccc2C(=O)O)cc1	37 (12)	0.38 / 0.14	0.2	

designs for FFAR1/sEH (top 12 from epochs 7-10)	SMILES	Abun-dance (rank)	Similarity FFAR1 / sEH	Sampling Temperature	ID
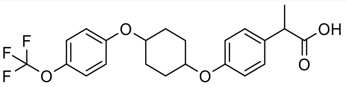	CC(C(=O)O)c1ccc(OC2CC<br>C(Oc3ccc(OC(F)(F)F)cc3)<br>CC2)cc1	6 (152)	0.24 / 0.27	0.7	
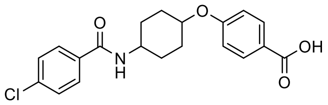	O=C(O)c1ccc(OC2CCC(N<br>C(=O)c3ccc(Cl)cc3)CC2)cc<br>1	2 (157)	0.18 / 0.32	0.7	
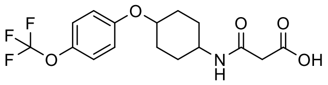	O=C(O)CC(=O)NC1CCC(<br>Oc2ccc(OC(F)(F)F)cc2)CC<br>1	1 (1296)	0.20 / 0.33	0.7	
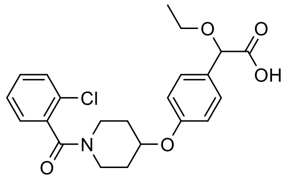	CCOC(C(=O)O)c1ccc(OC2<br>CCN(C(=O)c3ccccc3Cl)CC<br>2)cc1	1 (1296)	0.24 / 0.27	0.7	
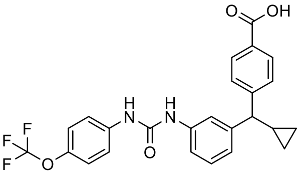	O=C(Nc1ccc(OC(F)(F)F)cc<br>1)Nc1cccc(C(c2ccc(C(=O)<br>O)cc2)C2CC2)c1	1 (1296)	0.24 / 0.26	0.7	**11**
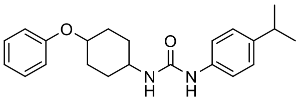	CC(C)c1ccc(NC(=O)NC2C<br>CC(Oc3ccccc3)CC2)cc1	1 (1296)	0.22 / 0.32	0.7	
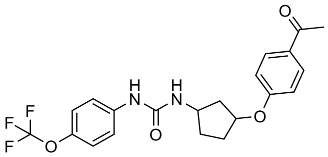	CC(=O)c1ccc(OC2CCC(N<br>C(=O)Nc3ccc(OC(F)(F)F)c<br>c3)C2)cc1	1 (1296)	0.17 / 0.33	0.7	
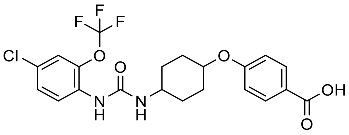	O=C(Nc1ccc(Cl)cc1OC(F)(<br>F)F)NC1CCC(Oc2ccc(C(=<br>O)O)cc2)CC1	1 (1296)	0.18 / 0.33	0.7	
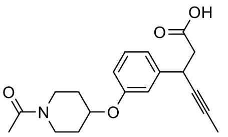	CC#CC(CC(=O)O)c1cccc(<br>OC2CCN(C(C)=O)CC2)c1	1 (1296)	0.29 / 0.25	0.7	
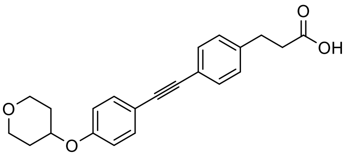	O=C(O)CCc1ccc(C#Cc2ccc<br>(OC3CCOCC3)cc2)cc1	1 (1296)	0.20 / 0.25	0.7	
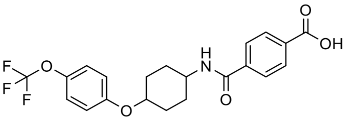	O=C(O)c1ccc(C(=O)NC2C<br>CC(Oc3ccc(OC(F)(F)F)cc3<br>)CC2)cc1	1 (1296)	0.18 / 0.35	0.7	
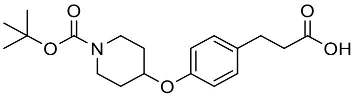	CC(C)(C)OC(=O)N1CCC(<br>Oc2ccc(CCC(=O)O)cc2)CC<br>1	1 (1296)	0.20 / 0.31	0.7	

designs for AT_1/FFAR1 (top 12 from epochs 34-38)	SMILES	Abundance (rank)	Similarity AT_1 / FFAR1	Sampling Temperature	ID
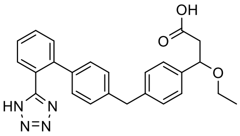	CCOC(CC(=O)O)c1ccc(Cc2ccc(-c3ccccc3-c3nnn[nH]3)cc2)cc1	686 (1)	0.27 / 0.21	0.2	
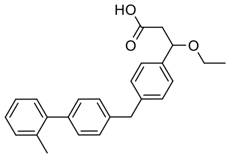	CCOC(CC(=O)O)c1ccc(Cc2ccc(-c3ccccc3C)cc2)cc1	488 (2)	0.26 / 0.23	0.2	
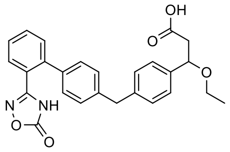	CCOC(CC(=O)O)c1ccc(Cc2ccc(-c3ccccc3-c3noc(=O)[nH]3)cc2)cc1	273 (3)	0.26 / 0.21	0.2	
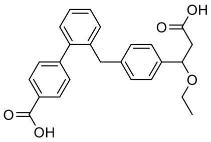	CCOC(CC(=O)O)c1ccc(Cc2ccccc2-c2ccc(C(=O)O)cc2)cc1	178 (4)	0.25 / 0.22	0.2	
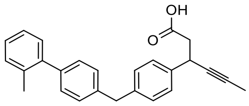	CC#CC(CC(=O)O)c1ccc(Cc2ccc(-c3ccccc3C)cc2)cc1	125 (5)	0.23 / 0.24	0.2	
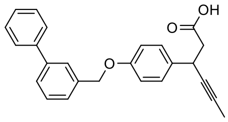	CC#CC(CC(=O)O)c1ccc(OCc2cccc(-c3ccccc3)c2)cc1	96 (6)	0.17 / 0.34	0.2	
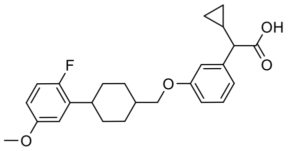	COc1ccc(F)c(C2CCC(COc3cccc(C(C(=O)O)C4CC4)c3)CC2)c1	67 (7)	0.14 / 0.32	0.2	
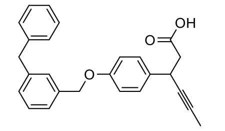	CC#CC(CC(=O)O)c1ccc(OCc2cccc(Cc3ccccc3)c2)cc1	59 (8)	0.16 / 0.33	0.2	
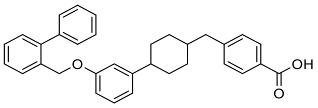	O=C(O)c1ccc(CC2CCC(c3cccc(OCc4ccccc4-c4ccccc4)c3)CC2)cc1	57 (9)	0.19 / 0.21	0.2	
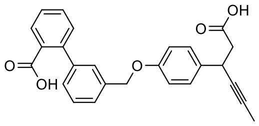	CC#CC(CC(=O)O)c1ccc(OCc2cccc(-c3ccccc3C(=O)O)c2)cc1	54 (10)	0.19 / 0.32	0.2	**12**
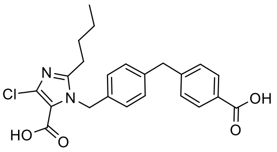	CCCCc1nc(Cl)c(C(=O)O)n1Cc1ccc(Cc2ccc(C(=O)O)cc2)cc1	51 (11)	0.37 / 0.13	0.2	
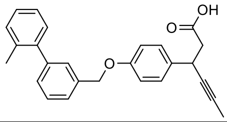	CC#CC(CC(=O)O)c1ccc(OCc2cccc(-c3ccccc3C)c2)cc1	43 (12)	0.20 / 0.32	0.2	

Suppl. Tab. 5. Retrospective application of the dual ligand design approach to the target pair PPARδ/FXR successfully re-designed compound **A** which has been previously developed as dual PPARδ / FXR agonist by rational design[19]. Compound **A** and various structural analogues (Tanimoto similarity to **A** > 0.5) were designed by a chemical language model (CLM) fine-tuned with the ligand sets for PPARδ and FXR (cf. Suppl. Tab. 1). The table shows the structure and abundance (sampling frequency over the selected epochs) of designs with Tanimoto similarity to **A** > 0.5 contained in the top 100 of 5000 molecules generated from selected epochs. **A** was removed from the CLM training/fine-tuning data.

Selected designs from epochs 43-47	SMILES	Abundance (rank)	Similarity to **A**[19]	Sampling Temperature
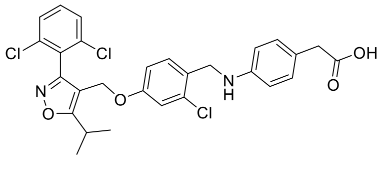 **A**	OC(CC1=CC=C(NCC2=CC=C(OCC3=C(C(C)C)ON=C3C4=C(C=CC=C4Cl)Cl)C=C2Cl)C=C1)=O	2 (52)	1.0	0.7
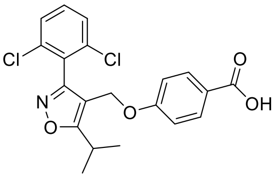	CC(C)c1onc(-c2c(Cl)cccc2Cl)c1COc1ccc(C(=O)O)cc1	2 (52)	0.61	0.7
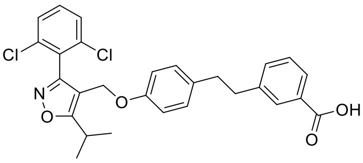	CC(C)c1onc(-c2c(Cl)cccc2Cl)c1COc1ccc(CCc2cccc(C(=O)O)c2)cc1	2 (52)	0.60	0.7
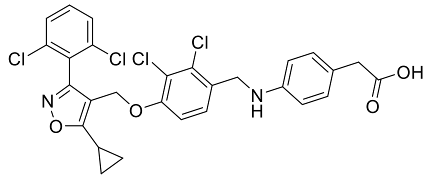	O=C(O)Cc1ccc(NCc2ccc(OCc3c(-c4c(Cl)cccc4Cl)noc3C3CC3)c(Cl)c2Cl)cc1	2 (52)	0.57	0.7
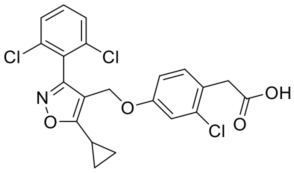	O=C(O)Cc1ccc(OCc2c(-c3c(Cl)cccc3Cl)noc2C2CC2)cc1Cl	6 (7)	0.56	0.7
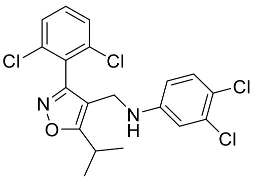	CC(C)c1onc(-c2c(Cl)cccc2Cl)c1CNc1ccc(Cl)c(Cl)c1	1 (1562)	0.54	0.7
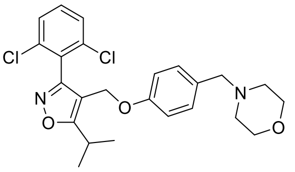	CC(C)c1onc(-c2c(Cl)cccc2Cl)c1COc1ccc(CN2CCOCC2)cc1	2 (52)	0.51	0.7
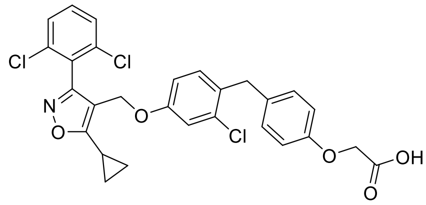	O=C(O)COc1ccc(Cc2ccc(OCc3c(-c4c(Cl)cccc4Cl)noc3C3CC3)cc2Cl)cc1	3 (26)	0.51	0.7

Suppl. Tab. 6. Most similar compounds in ChEMBL for each designed and experimentally tested dual ligand (FT-Set - Fine-tuning set).

Design (ID)	Most similar FXR agonist	Similarity	Contained in FT-Set	Most similar sEH inhibitor	Similarity	Contained in FT-Set
**1**	CHEMBL4079796	0.71	No	CHEMBL4096300	0.79	No
**2**	CHEMBL4104364	0.66	No	CHEMBL3765559	0.61	No
**3**	CHEMBL4078618	0.48	No	CHEMBL3765559	0.55	No
Design (ID)	Most similar FXR agonist	Similarity	Contained in FT-Set	Most similar THRβ agonist	Similarity	Contained in FT-Set
**4**	CHEMBL4592387	1.0	No	CHEMBL40738	0.32	No
**5**	CHEMBL4636295	0.96	No	CHEMBL393489	0.39	No
**6**	CHEMBL4636295	0.77	No	CHEMBL4778737	0.36	No
Design (ID)	Most similar PPARδ agonist	Similarity	Contained in FT-Set	Most similar sEH inhibitor	Similarity	Contained in FT-Set
**7**	CHEMBL247507	0.27	No	CHEMBL3677984	0.60	No
**8**	CHEMBL391180	0.33	No	CHEMBL589621	0.58	No
**9**	CHEMBL391180	0.33	No	CHEMBL589621	0.62	No

Supplementary Methods

Chemistry

4'-{[(2,6-Dichlorophenyl)methyl]carbamoyl}[1,1'-biphenyl]-4-carboxylic acid (3).

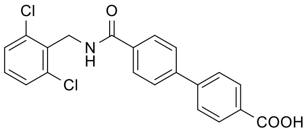

The synthesis was performed according to a modified literature procedure.[20] *N*-(2,6-Dichlorobenzyl)-4-iodobenzamide (**15,** 0.1 g, 0.3 mmol, 1 eq) and Na_2CO_3 (0.08 g, 0.7 mmol, 3 eq) were dissolved in a 4:1 mixture of degassed 1,4-dioxane (10 mL) and H_2O (2.5 mL). 4-Boronobenzoic acid (0.05 g, 0.3 mmol, 1.2 eq) and a catalytic amount of $Pd(PPh_3)_4$ (0.01 g, 0.01 mmol, 0.05 eq) were added. The reaction mixture was heated under reflux for 4 h. After cooling to rt, an aqueous HCl solution (10%, 20 mL) was added, the mixture extracted with EtOAc (3x30 mL) and dried over Na_2SO_4. The product was purified by CC (hexane/EtOAc = 2:1 + 2% AcOH) to obtain **3** as colorless crystals (0.09 g, 0.2 mmol, 91%). ^{1}H NMR (500 MHz, DMSO-d_6) δ = 8.75 (s, 1H), 8.00 (d, J = 28.8, 7.4 Hz, 4H), 7.81 (d, J = 6.5 Hz, 4H), 7.63–7.37 (m, 3H), 4.72 (s, 2H) ppm. ^{13}C NMR (126 MHz, DMSO-d_6) δ = 167.6, 165.9, 142.6, 141.9, 135.9, 133.5, 133.3, 130.2, 130.0, 128.6, 128.3, 126.8, 126.7, 39.8 ppm. HRMS (ESI$^-$): m/z calcd for $C_{21}H_{14}Cl_2NO_3$: 398.03562, found: 398.03556 ([M-H]$^-$).

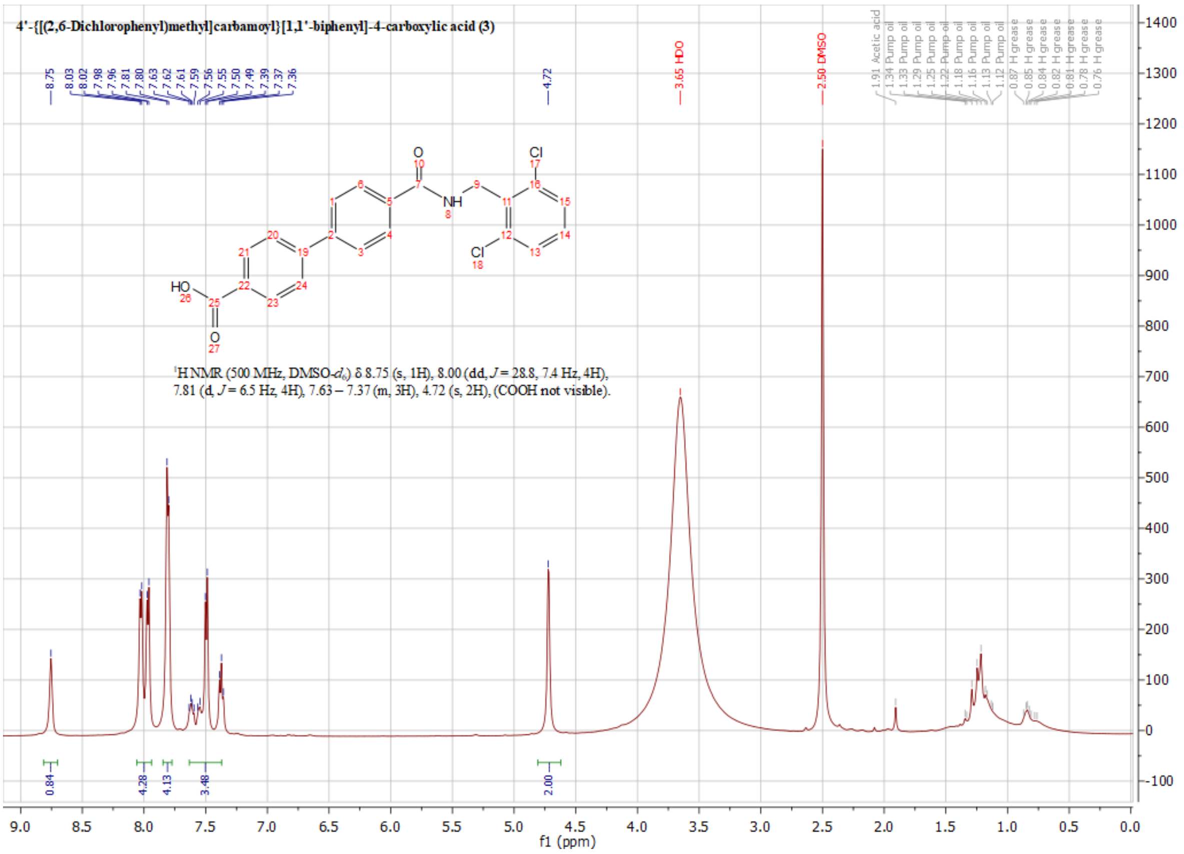

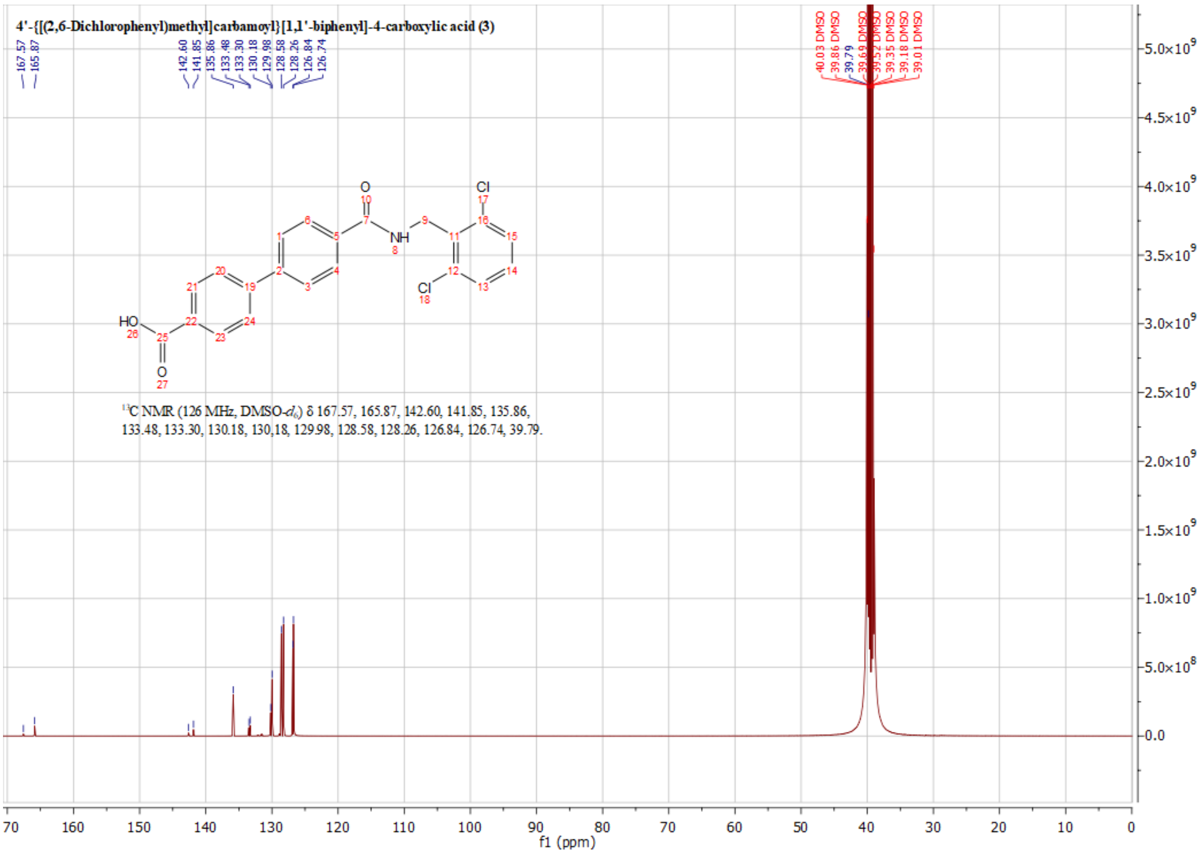
4'-{[(2,6-Dichlorophenyl)methyl]carbamoyl}[1,1'-biphenyl]-4-carboxylic acid (3)
167.57
165.87
142.60
141.85
135.86
133.48
133.30
130.18
129.98
128.58
128.26
126.84
126.74
40.03 DMSO
39.86 DMSO
39.79
39.69 DMSO
39.52 DMSO
39.35 DMSO
39.18 DMSO
39.01 DMSO
¹³C NMR (126 MHz, DMSO-d₆) δ 167.57, 165.87, 142.60, 141.85, 135.86, 133.48, 133.30, 130.18, 130,18, 129.98, 128.58, 128.26, 126.84, 126.74, 39.79.
HO
O
NH
Cl
Cl
f1 (ppm)

4-{[3-(2,6-Dichlorophenyl)-5-isopropylisoxazol-4-yl]methoxy}benzoic acid (4).

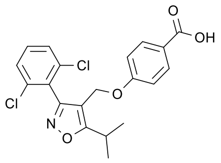

The synthesis was performed according to a modified literature procedure.[21] Ethyl-4-{[3-(2,6-dichlorophenyl)-5-isopropylisoxazol-4-yl]methoxy}benzoate (**22**, 0.12 g, 0.26 mmol, 1.0 eq) was dissolved in a 1:1 mixture of THF (3 mL) and EtOH (3 mL). LiOH·H_2O (0.05 g, 1 mmol, 5 eq) was dissolved in H_2O (3 mL). Both solutions were combined and stirred under reflux for 3 h. The reaction mixture was allowed to cool to rt and an aqueous HCl solution (10%, 20 mL) was added to quench the reaction. The aqueouos layer was extracted with EtOAc (3x30 mL). The combined organic layers were dried over Na_2SO_4 and the solvent was evaporated under reduced pressure. Further purification was performed by CC (hexane/EtOAc = 95.8:4.2 → 66.7:33.3) to obtain **4** as light yellow crystals (0.05 g, 0.1 mmol, 47%). ^{1}H NMR (500 MHz, Acetone-*d*$_6$) δ = 7.91 (d, *J* = 8.9 Hz, 2H), 7.58–7.49 (m, 3H), 6.91 (d, *J* = 8.9 Hz, 2H), 4.98 (s, 2H), 3.51 (hept, *J* = 7.0 Hz, 1H), 1.41 (d, *J* = 7.0 Hz, 6H) ppm. ^{13}C NMR (126 MHz, Acetone-*d*$_6$) δ = 177.1, 167.2, 163.0, 159.9, 136.2, 132.9, 132.4, 129.2, 128.9, 124.2, 115.2, 110.3, 0.2, 27.5, 21.1 ppm. HRMS (MALDI$^+$): *m/z* calcd for $C_{20}H_{18}Cl_2NO_4$: 406.06074, found: 406.06083 ([M+H]$^+$).

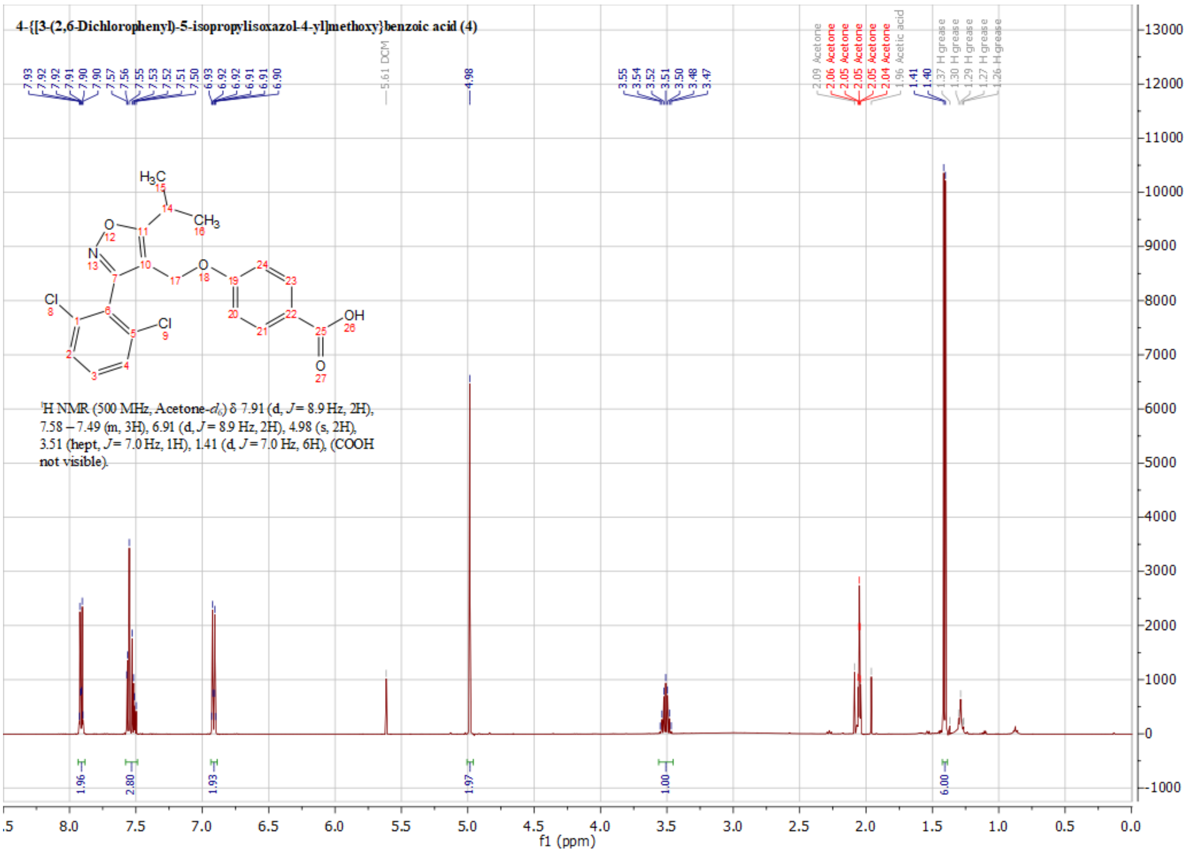

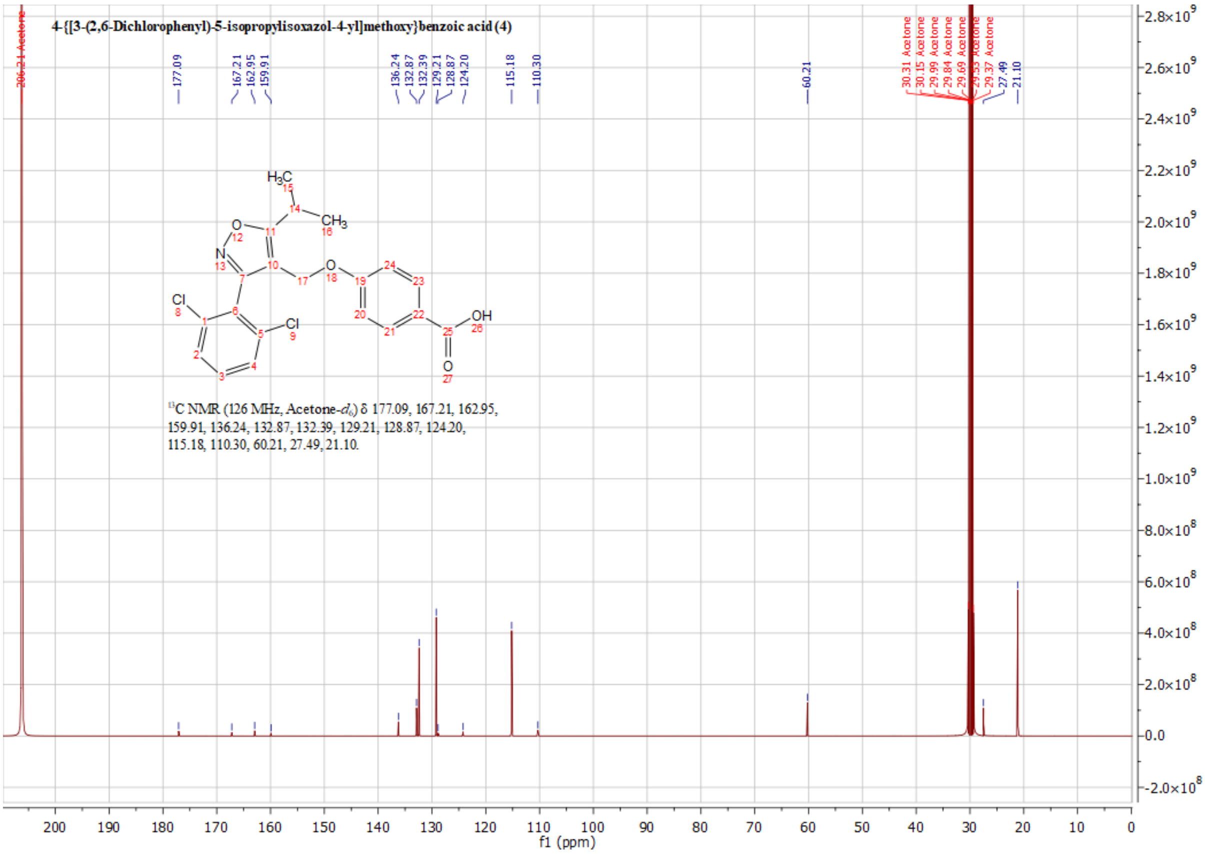
4-{[3-(2,6-Dichlorophenyl)-5-isopropylisoxazol-4-yl]methoxy}benzoic acid (4)
^{13}C NMR (126 MHz, Acetone-d_6) δ 177.09, 167.21, 162.95, 159.91, 136.24, 132.87, 132.39, 129.21, 128.87, 124.20, 115.18, 110.30, 60.21, 27.49, 21.10.
f1 (ppm)

2-(4-{[3-(2,6-Dichlorophenyl)-5-isopropylisoxazol-4-yl]methoxy}phenyl)acetic acid (5).

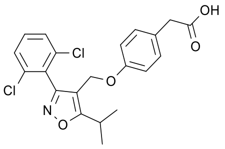

The synthesis was performed according to a modified literature procedure.[21] Ethyl 2-(4-[{3-(2,6-dichlorophenyl)-5-isopropylisoxazol-4-yl]methoxy}phenyl)acetate (**23**, 0.06 g, 0.1 mmol, 1 eq) was dissolved in a 1:1 mixture of THF (3 mL) and EtOH (3 mL). LiOH·H_2O (55 mg, 1.3 mmol, 5 eq) was dissolved in H_2O (3 mL). Both solutions were combined and stirred under reflux for 3 h. The reaction mixture was allowed to cool to rt and an aqueous HCl solution (10%, 20 mL) was added to quench the reaction. The aqueous layer was extracted with EtOAc (3x30 mL). The combined organic layers were dried over Na_2SO_4 and the solvent was evaporated under reduced pressure. Further purification was performed by CC (hexane/EtOAc = 95.8:4.2 → 66.7:33.3) to obtain **5** as light yellow crystals (0.05 g, 0.1 mmol, 87%). ^{1}H NMR (500 MHz, $CDCl_3$) δ = 7.33 (d, J = 8.1, 0.8 Hz, 2H), 7.25–7.19 (m, 1H), 7.06 (d, J = 8.7 Hz, 2H), 6.68 (d, J = 8.7 Hz, 2H), 4.64 (s, 2H), 3.49 (s, 2H), 3.26 (hept, J = 7.0 Hz, 1H), 1.35 (d, J = 7.0 Hz, 6H) ppm. ^{13}C NMR (126 MHz, $CDCl_3$) δ = 178.0, 176.5, 159.2, 157.6, 135.9, 131.4, 130.5, 128.2, 127.9, 126.1, 115.1, 109.5, 59.5, 40.2, 27.2, 20.9 ppm. HRMS (MALDI$^+$): m/z calcd for $C_{21}H_{20}Cl_2NO_4$: 420.07639, found: 420.07612 ([M+H]$^+$).

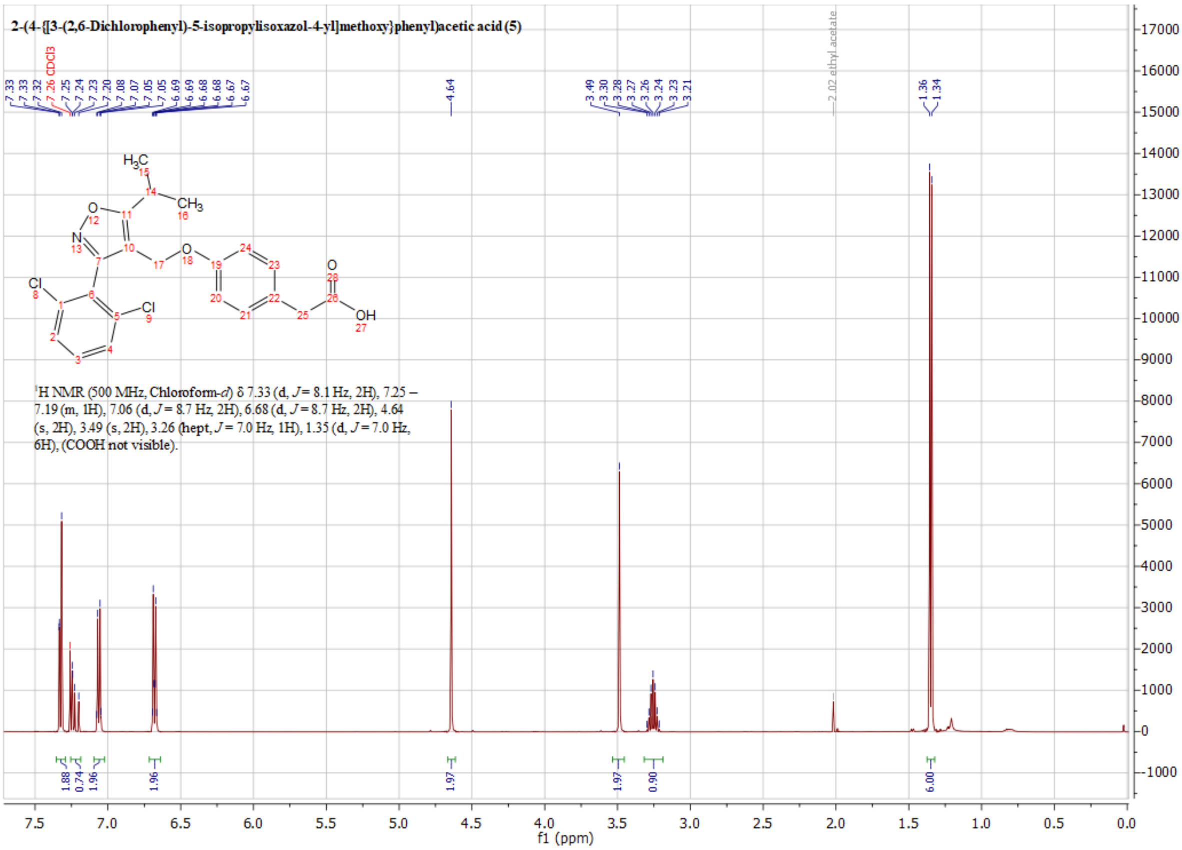

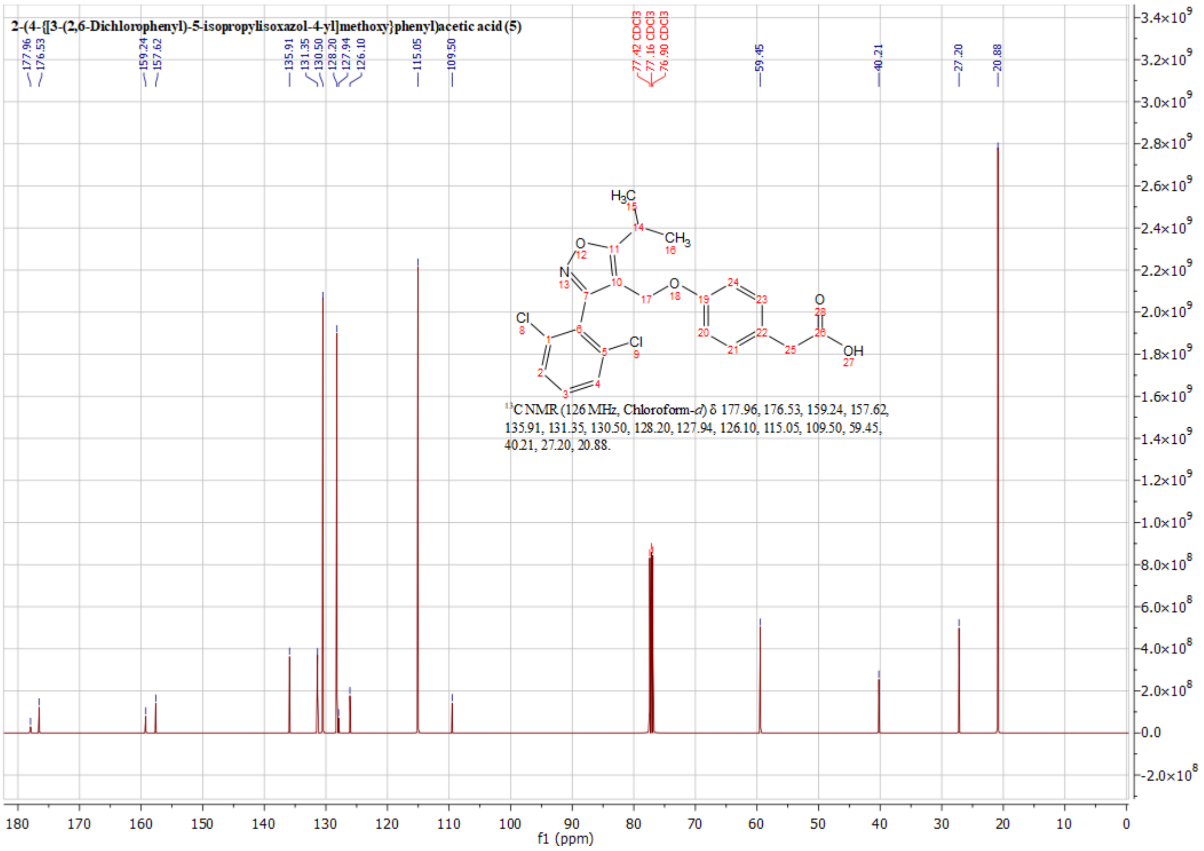
2-(4-{[3-(2,6-Dichlorophenyl)-5-isopropylisoxazol-4-yl]methoxy}phenyl)acetic acid (5)
177.96
176.53
159.24
157.62
135.91
131.35
130.50
128.20
127.94
126.10
115.05
109.50
77.42 CDCl3
77.16 CDCl3
76.90 CDCl3
59.45
40.21
27.20
20.88
13C NMR (126 MHz, Chloroform-d) δ 177.96, 176.53, 159.24, 157.62, 135.91, 131.35, 130.50, 128.20, 127.94, 126.10, 115.05, 109.50, 59.45, 40.21, 27.20, 20.88.
f1 (ppm)

2-[4-(4-{[3-(2,6-Dichlorophenyl)-5-isopropylisoxazol-4-yl]methoxy}benzyl)phenoxy]acetic acid (6).

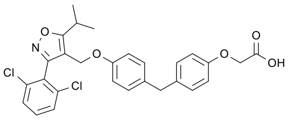

The synthesis was performed according to a modified literature procedure.[21] Ethyl 2-[4-(4-{[3-(2,6-dichlorophenyl)-5-isopropylisoxazol-4-yl]methoxy}benzyl)phenoxy]acetate (**20**, 0.23 g, 0.41 mmol, 1.0 eq) was dissolved in a 1:1 mixture of THF (5 mL) and EtOH (5 mL). LiOH·H_2O (0.34 g, 8.2 mmol, 20 eq) was dissolved in H_2O (5 mL). Both solutions were combined and stirred under reflux for 3 h. After cooling to rt, an aqueous HCl solution (10%, 20 mL) was added to quench the reaction. The aqueous layer was extracted with EtOAc (3x30 mL). The combined organic layers were dried over Na_2SO_4 and the solvent was evaporated under reduced pressure. The product was purified by CC (hexane/EtOAc = 1:1 + 2% AcOH) to obtain **6** as yellow oil (0.2 g, 0.4 mmol, 93%). ^{1}H NMR (500 MHz, Acetone-d_6) δ = 7.52–7.43 (m, 3H), 7.09 (d, J = 8.8 Hz, 2H), 7.04 (d, J = 8.7 Hz, 2H), 6.85 (d, J = 8.7 Hz, 2H), 6.73 (d, J = 8.7 Hz, 2H), 4.82 (s, 2H), 4.66 (s, 2H), 3.80 (s, 2H), 3.43 (hept, J = 7.0 Hz, 1H), 1.36 (d, J = 7.0 Hz, 6H) ppm. ^{13}C NMR (126 MHz, Acetone-d_6) δ = 176.9, 170.5, 159.9, 157.5, 157.3, 136.2, 135.4, 135.3, 132.7, 130.4, 130.4, 129.1, 128.9, 115.6, 115.3, 110.7, 65.4, 60.0, 40.6, 27.4, 21.1 ppm. HRMS (ESI$^+$): m/z calcd for $C_{28}H_{26}Cl_2NO_5$: 526.11825, found: 526.11771 ([M+H]$^+$).

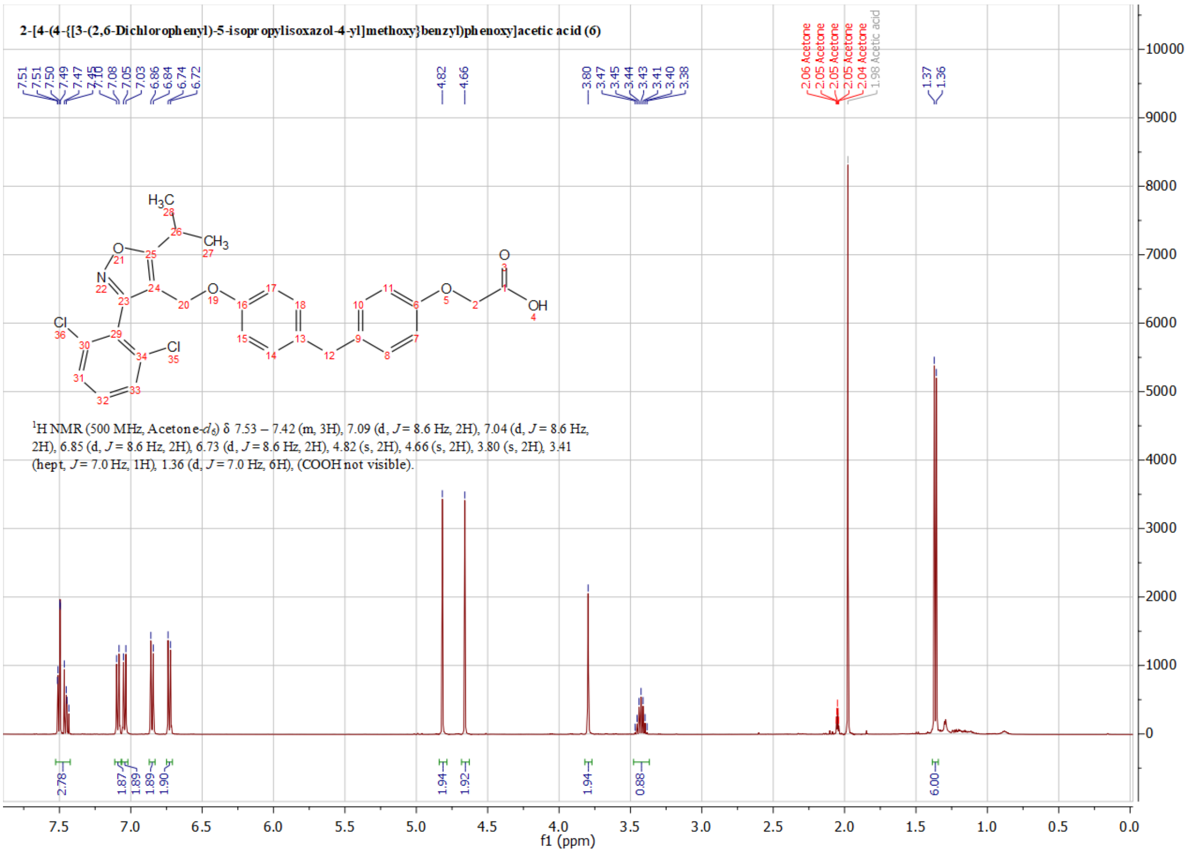

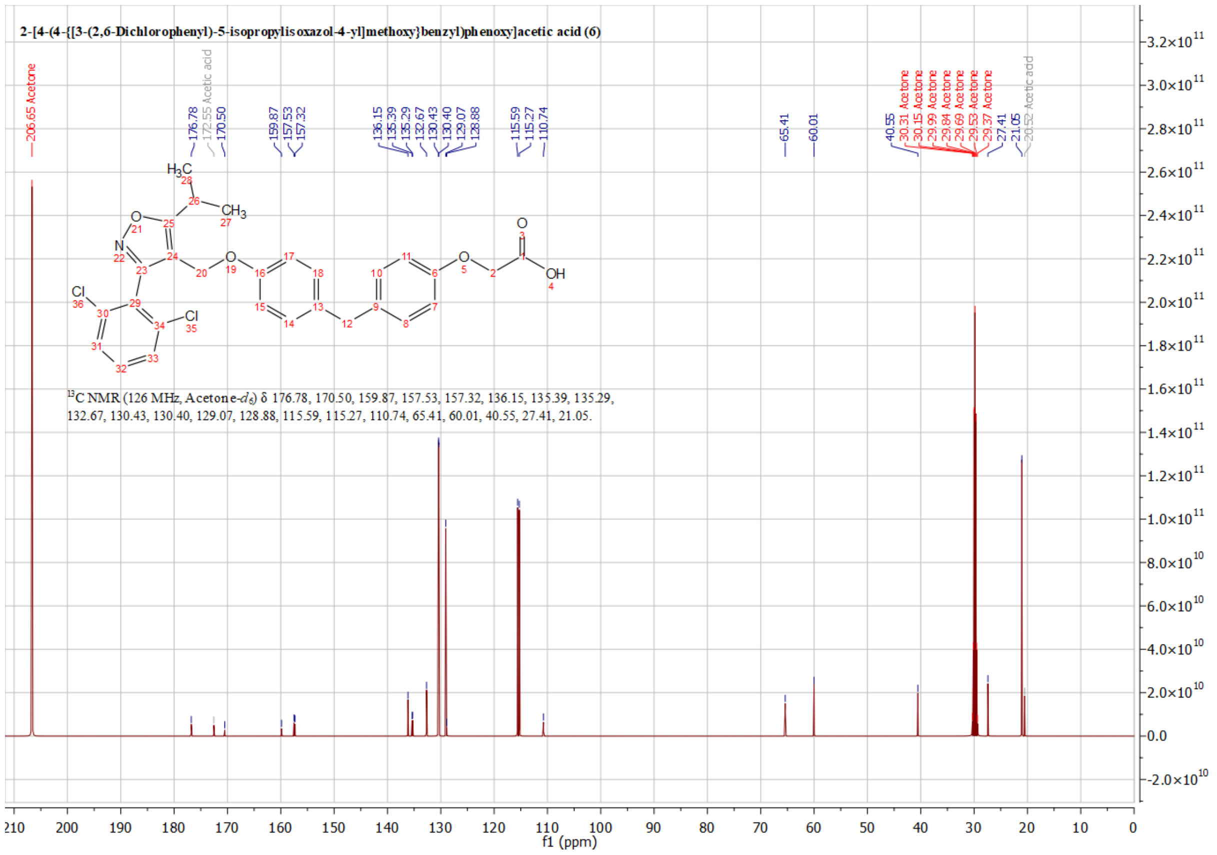
2-[4-(4-{[3-(2,6-Dichlorophenyl)-5-isopropylisoxazol-4-yl]methoxy}benzyl)phenoxy]acetic acid (6)
^{13}C NMR (126 MHz, Acetone-d_6) δ 176.78, 170.50, 159.87, 157.53, 157.32, 136.15, 135.39, 135.29, 132.67, 130.43, 130.40, 129.07, 128.88, 115.59, 115.27, 110.74, 65.41, 60.01, 40.55, 27.41, 21.05.
206.65 Acetone
172.55 Acetic acid
20.52 Acetic acid
f1 (ppm)

3-(4-{[*trans*-4-{[(4-*tert*-Butylphenyl)carbamoyl]amino}cyclohexyl]oxy}phenyl)propanoic acid (7).

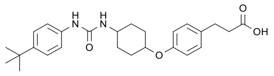

Synthesis was performed according to a standard procedure for ester deprotection.[22] Methyl 3-(4-{[trans-4-{[(4-tert-butylphenyl)carbamoyl]amino}cyclohexyl]oxy}phenyl)propanoate (**32**, 25 mg, 0.055 mmol, 1.0 eq) was dissolved in 1,4-dioxane (0.6 mL).An aqueous LiOH solution (0.4 M, 0.20 mL, 0.11 mmol, 2.0 eq) was added and the reaction stirred at rt for 20 h. Upon full conversion, H_2O was added, and the aqueous layer washed with EtOAc. Then, the aqueous layer was brought to pH 2 with an aqueous HCl solution (2 M) and the aqueous layer extracted with EtOAc (3x). The combined organic layers were dried over $MgSO_4$, filtered and the solvent removed under reduced pressure. Product **7** was obtained as colourless crystals (16 mg, 0.037 mmol, 66%). Purity: 96% (qHNMR). ^{1}H NMR (500 MHz, MeOD-d$_4$): δ = 7.29 (d, J = 9.1 Hz, 2H), 7.24 (d, J = 9.1 Hz, 2H), 7.12 (d, J = 8.8 Hz, 2H), 6.83 (d, J = 8.8 Hz, 2H), 4.29–4.18 (m, 1H), 3.69–3.57 (m, 1H), 2.84 (t, J = 7.7 Hz, 2H), 2.55 (t, J = 7.7 Hz, 2H), 2.16–1.99 (m, 4H), 1.60–1.47 (m, 2H), 1.44–1.31 (m, 2H), 1.29 (s, 9H) ppm. ^{13}C-NMR (126 MHz, MeOD-d$_4$): δ = 176.9, 157.9, 157.5, 146.4, 138.2, 134.5, 130.3, 126.6, 120.1, 117.2, 76.2, 49.9, 37.1, 35.0, 31.9, 31.6, 31.3, 31.2 ppm. MS (APCI+): m/z 439.2 ([M+H]$^+$). HRMS (ESI+): m/z calcd for $C_{26}H_{35}N_2O_4$: 439.25913, found 439.25882 ([M+H]$^+$).

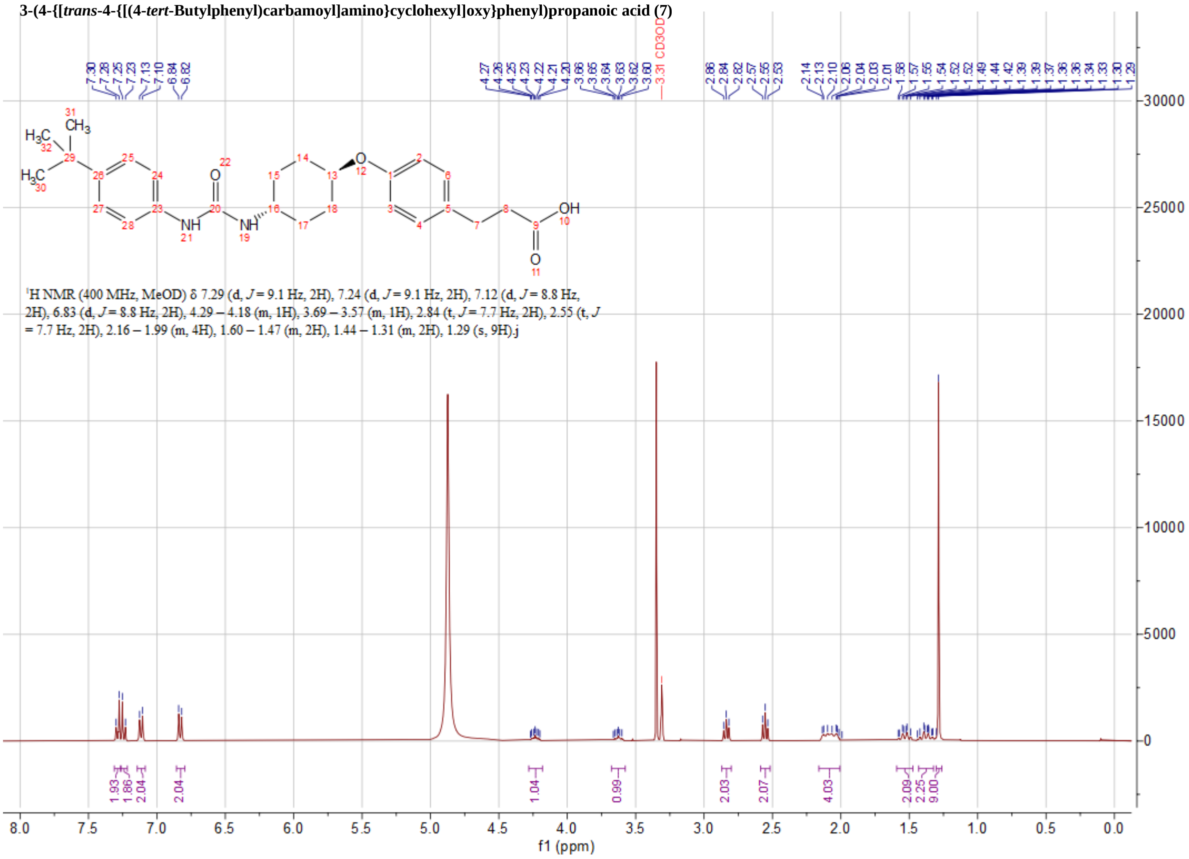

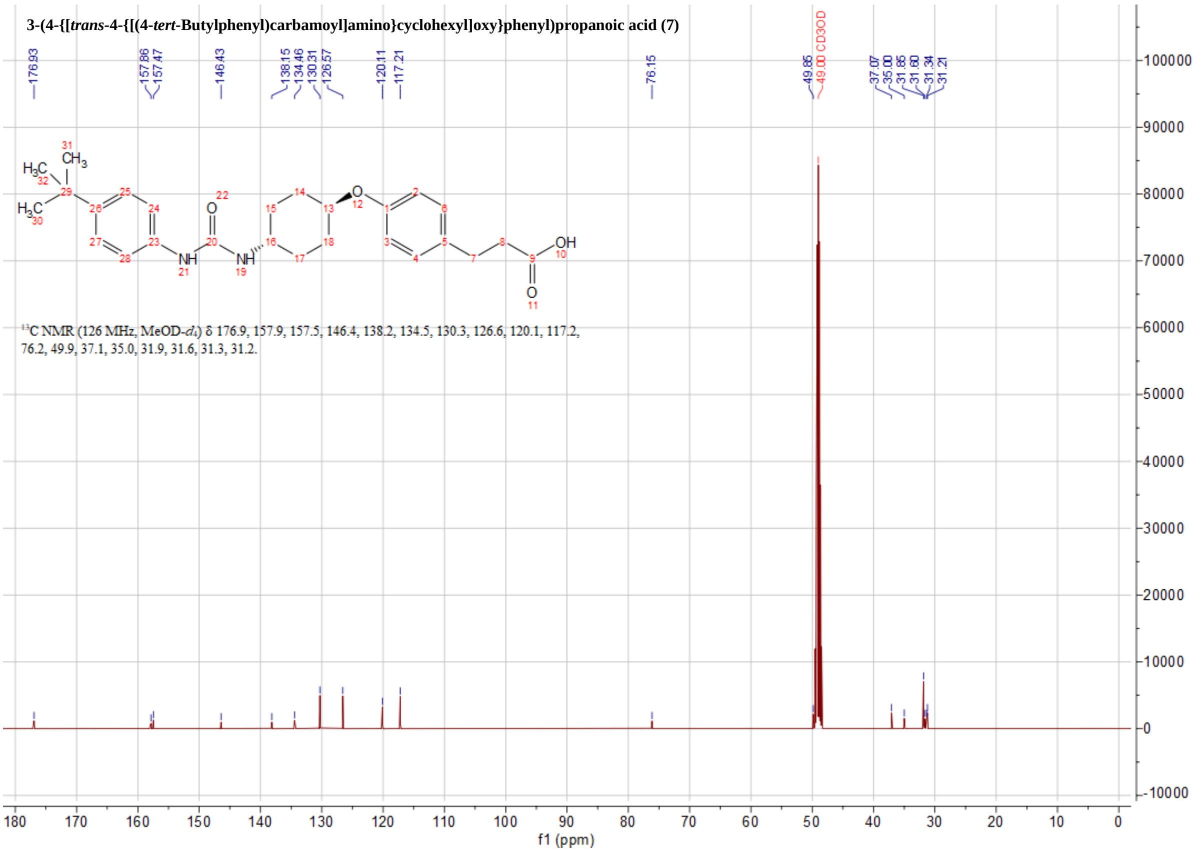
3-(4-{[trans-4-{[(4-tert-Butylphenyl)carbamoyl]amino}cyclohexyl]oxy}phenyl)propanoic acid (7)
^{13}C NMR (126 MHz, MeOD-d_4) δ 176.9, 157.9, 157.5, 146.4, 138.2, 134.5, 130.3, 126.6, 120.1, 117.2, 76.2, 49.9, 37.1, 35.0, 31.9, 31.6, 31.3, 31.2.
176.93
157.86
157.47
146.43
138.15
134.46
130.31
126.57
120.11
117.21
76.15
49.85
49.00 CD3OD
37.07
35.00
31.85
31.60
31.34
31.21
f1 (ppm)

3-[4-({1-[(2,4,6-Trichlorobenzyl)carbamoyl]azepan-4-yl}oxy)phenyl] propanoic acid (8).

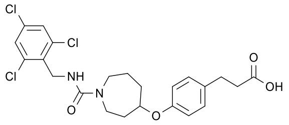

Synthesis was performed according to a standard procedure for ester deprotection and not under inert atmosphere.[22] Methyl 3-[4-({1-[(2,4,6-trichlorobenzyl)carbamoyl]azepan-4-yl}oxy)phenyl]propanoate (**39**, 21 mg, 0.041 mmol, 1.0 eq) was dissolved in 1,4-dioxane (0.20 mL). LiOH (0.4 M, aqu., 0.20 mL, 0.082 mmol, 2.0 eq) was added and the reaction stirred at rt for 20 h. Upon full conversion, H_2O was added, and the aqueous layer washed with EtOAc. Then, the aqueous layer was brought to pH 2 with aqueous HCl solution (2 M) and the aqueous layer extracted with EtOAc (3x). The combined organic layers were dried over $MgSO_4$, filtered and the solvent removed under reduced pressure. Product **8** was obtained as colourless crystals (14 mg, 0.028 mmol, 69%) with 97% purity. ^{1}H NMR (500 MHz, CD_2Cl_2): δ = 7.37 (s, 2H), 7.10 (d, J = 8.6 Hz, 2H), 6.77 (d, J = 8.6 Hz, 2H), 4.81 (t, J = 5.6 Hz, 1H), 4.64 (d, J = 5.6 Hz, 2H), 4.43–4.36 (m, 1H), 3.66–3.37 (m, 3H), 3.33–3.24 (m, 1H), 2.86 (t, J = 7.7 Hz, 2H), 2.61 (t, J = 7.7 Hz, 2H), 2.04–1.80 (m, 5H), 1.69–1.57 (m, 1H) ppm. ^{13}C NMR (126 MHz, CD_2Cl_2): δ = 176.8, 157.5, 156.2, 137.0, 134.4, 134.1, 133.3, 129.7, 128.8, 116.4, 75.7, 46.5, 41.3, 40.6, 36.0, 34.4, 32.0, 30.2, 22.8 ppm. MS (APCI+): m/z 498.4 ([M+H]$^+$). HRMS (ESI+): m/z calcd for $C_{23}H_{26}Cl_3N_2O_4$: 499.09582, found 499.09552 ([M+H]$^+$).

3-[4-({1-[(2,4,6-Trichlorobenzyl)carbamoyl]azepan-4-yl}oxy)phenyl] propanoic acid (8)

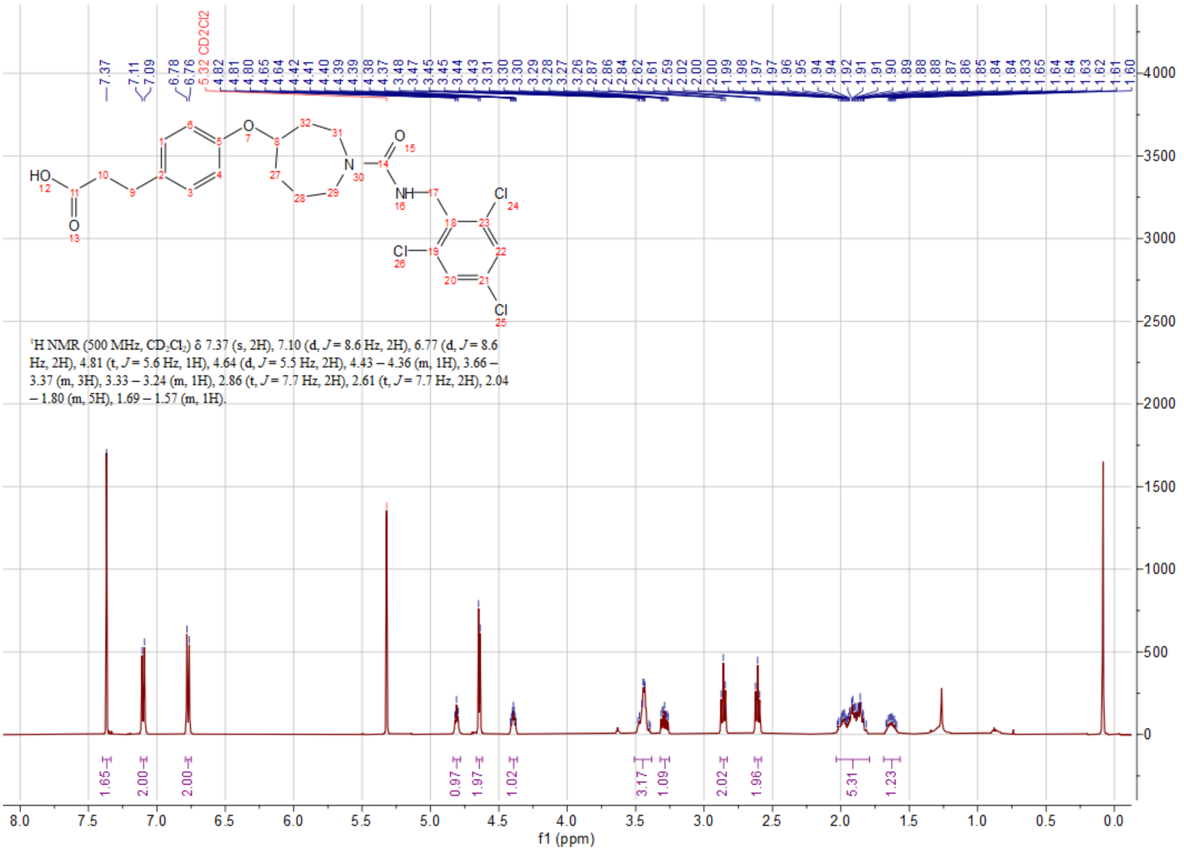

3-[4-({1-[(2,4,6-Trichlorobenzyl)carbamoyl]azepan-4-yl}oxy)phenyl] propanoic acid (8)

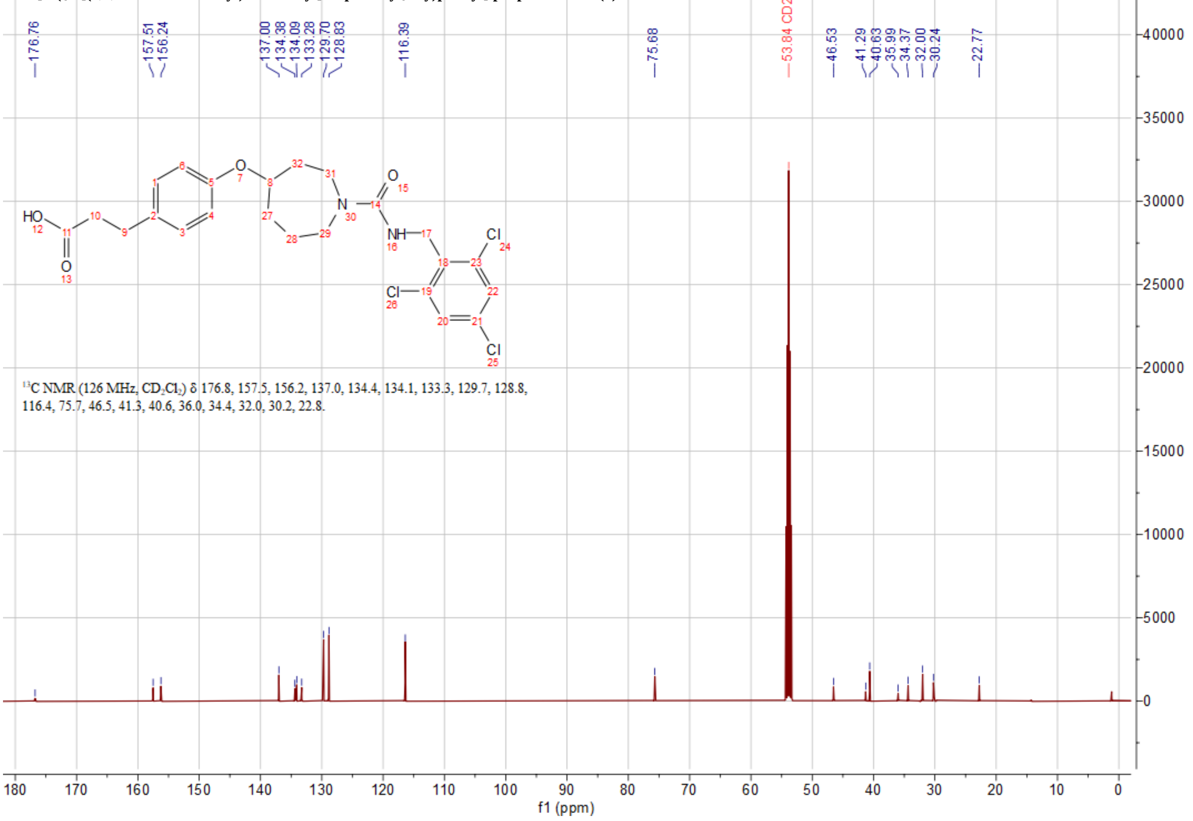

^{13}C NMR (126 MHz, CD_2Cl_2) δ 176.8, 157.5, 156.2, 137.0, 134.4, 134.1, 133.3, 129.7, 128.8, 116.4, 75.7, 46.5, 41.3, 40.6, 36.0, 34.4, 32.0, 30.2, 22.8.

3-[4-({1-[(2,4,6-Trichlorobenzyl)carbamoyl]piperidin-4-yl}oxy)phenyl]propanoic acid (9).

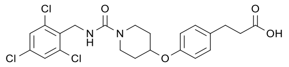

Synthesis was performed according to a standard procedure for ester deprotection and not under inert atmosphere.[22] Methyl 3-[4-({1-[(2,4,6-trichlorobenzyl)carbamoyl]piperidin-4-yl}oxy)phenyl]propanoate (**38**, 18 mg, 0.036 mmol, 1.0 eq) was dissolved in 1,4-dioxane (0.20 mL). An aqueous LiOH solution (0.4 M, 0.20 mL, 0.071 mmol, 2.0 eq) was added and the reaction stirred at rt for 3 h. Upon full conversion, H_2O was added, and the aqueous layer washed with EtOAc. Then, the aqueous layer was brought to pH 2 with aqueous HCl solution (2 M) and the aqueous layer extracted with EtOAc (3x). The combined organic layers were dried over $MgSO_4$, filtered and the solvent removed under reduced pressure. Purification was performed by precipitation with isohexane from the solution in DCM giving product **9** as beige crystals (13 mg, 0.027 mmol, 74%) Purity: 96% (qHNMR). ^{1}H NMR (400 MHz, MeOD-d$_4$): δ = 7.47 (s, 2H), 7.12 (d, J = 8.6 Hz, 2H), 6.85 (d, J = 8.6 Hz, 2H), 4.60 (s, 2H), 4.55–4.45 (m, 1H), 3.72–3.62 (m, 2H), 3.30–3.24 (m, 2H), 2.83 (t, J = 7.6 Hz, 2H), 2.55 (t, J = 7.7 Hz, 2H), 1.97–1.85 (m, 2H), 1.70–1.58 (m, 2H) ppm. ^{13}C NMR (101 MHz, MeOD-d$_4$): δ = 176.8, 159.7, 157.0, 138.2, 135.2, 134.7, 134.5, 130.4, 129.4, 117.3, 73.4, 42.2, 41.4, 37.0, 31.7, 31.2 ppm. MS (APCI+): m/z = 484.5 ([M+H]$^+$). HRMS (ESI+): m/z calcd for $C_{22}H_{24}Cl_3N_2O_4$: 485.08017, found 485.07985 ([M+H]$^+$).

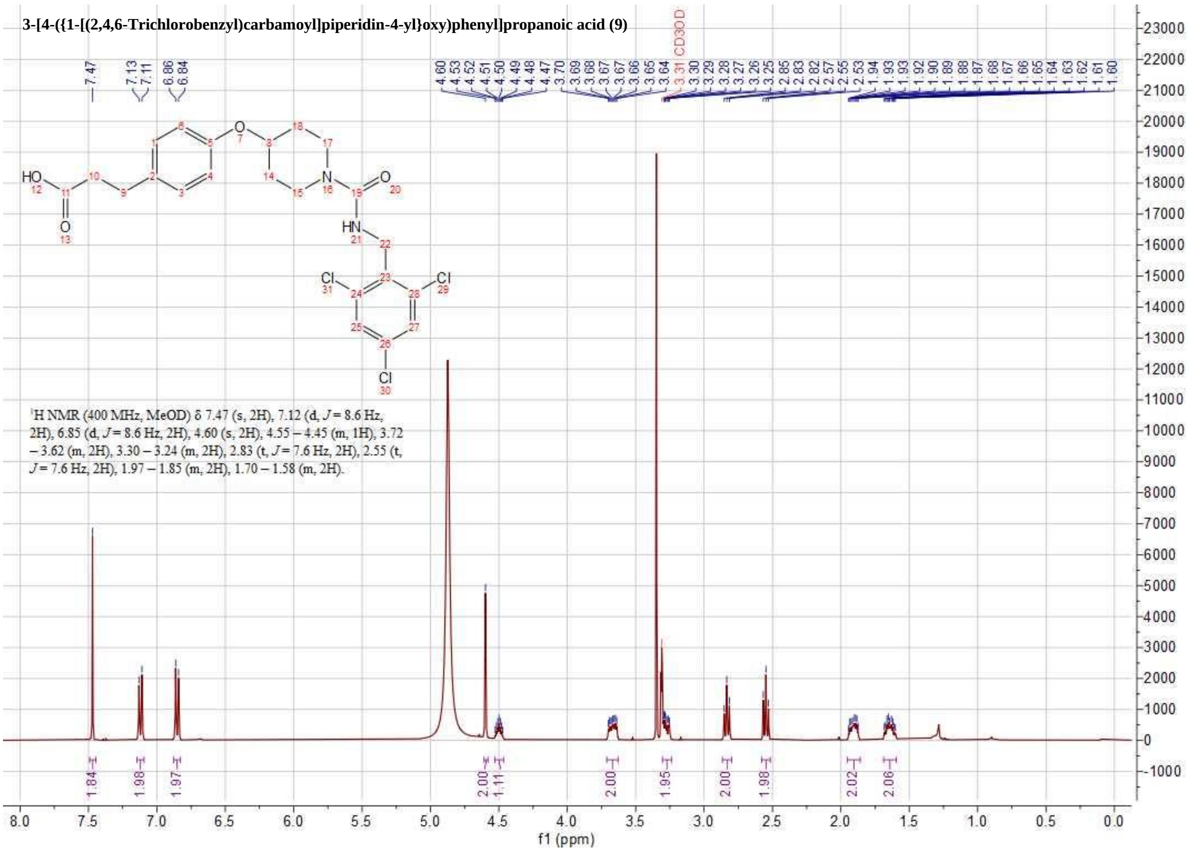

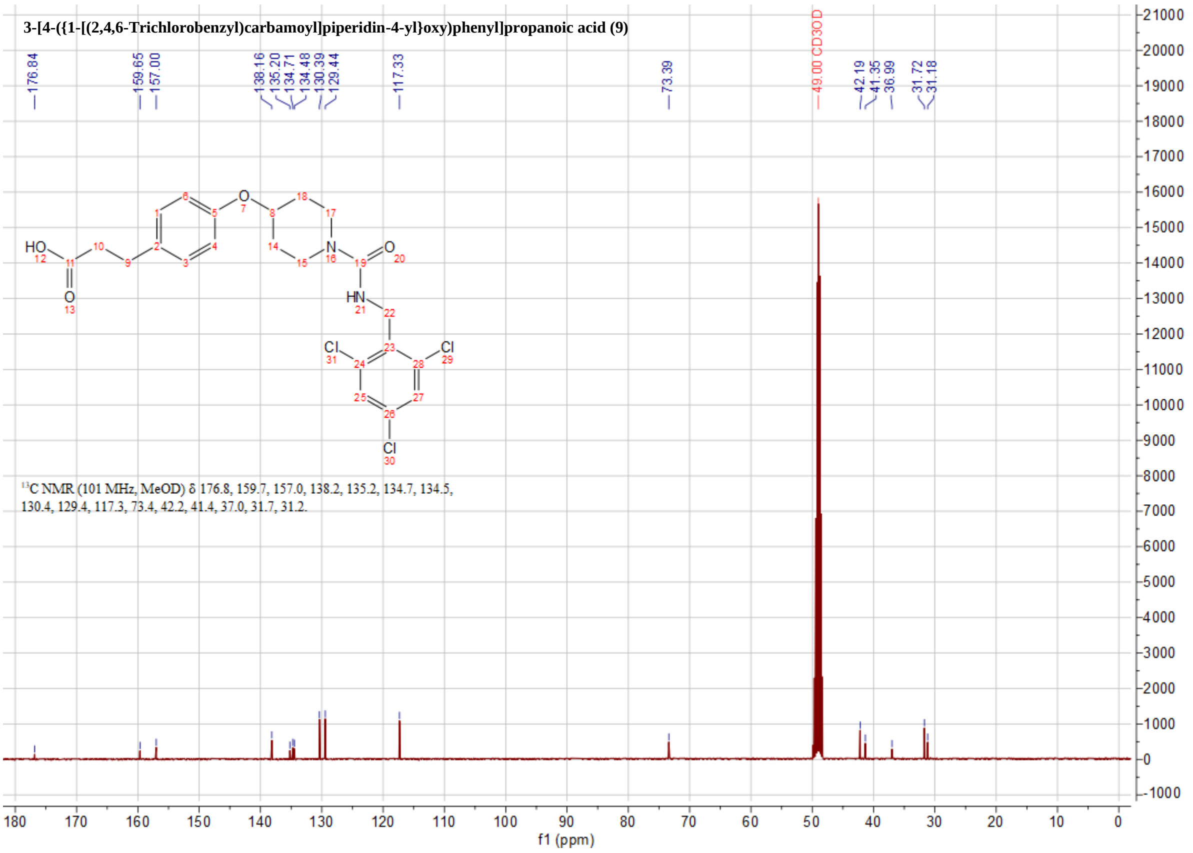
3-[4-({1-[(2,4,6-Trichlorobenzyl)carbamoyl]piperidin-4-yl}oxy)phenyl]propanoic acid (9)
176.84
159.65
157.00
138.16
135.20
134.71
134.48
130.39
129.44
117.33
73.39
49.00 CD3OD
42.19
41.35
36.99
31.72
31.18
13C NMR (101 MHz, MeOD) δ 176.8, 159.7, 157.0, 138.2, 135.2, 134.7, 134.5, 130.4, 129.4, 117.3, 73.4, 42.2, 41.4, 37.0, 31.7, 31.2.
f1 (ppm)

4-({1-[(2,4-Dichlorobenzyl)carbamoyl]azepan-4-yl}oxy)benzoic acid (**10**).

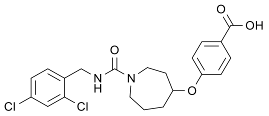

Methyl 4-({1-[(2,4-dichlorobenzyl)carbamoyl]azepan-4-yl}oxy)benzoate (**45,** 35 mg, 0.078 mmol, 1.0 eq) was dissolved in THF (10 mL), and LiOH (19 mg, 0.78 mmol, 10 eq) dissolved in H_2O (0.5 mL) was added. Subsequently, MeOH was added until a homogeneous (monophasic) solution was obtained. The reaction was stirred at RT until full conversion of the starting material (2 days). Afterwards the solvents were removed under reduced pressure and the residue was taken up in EtOAc (30 mL) and aqueous 2N HCL-solution (20 mL). The phases were separated, and the aqueous phase was extracted with EtOAc (3x20 mL). The combined organic phases were dried over $MgSO_4$ and the solvent was removed under reduced pressure. The crude product was purified by reverse phase CC (H_2O/ACN = 90:10 → 0:100). **10** was obtained as a colourless solid (25 mg, 0.057 mmol, 74%). t_R: 19.08 min (HPLC), purity: 99% (254 nm). ^1H NMR (500 MHz, MeOD-d$_4$) δ = 7.97–7.94 (m, 2H), 7.42 (d, J = 2.1 Hz, 1H), 7.34 (d, J = 8.3 Hz, 1H), 7.28 (dd, J = 8.3, 2.1 Hz, 1H), 6.97–6.94 (m, 2H), 4.67 (tt, J = 7.2, 3.2 Hz, 1H), 4.44 (s, 2H), 3.62–3.46 (m, 4H), 2.16–2.10 (m, 1H), 2.03–1.91 (m, 4H), 1.80–1.73 (m, 1H) ppm. ^{13}C NMR (500 MHz, MeOD-d$_4$) δ = 169.8, 162.9, 159.9, 137.8, 134.6, 134.2, 132.9, 130.7, 129.9, 128.2, 124.1, 116.3, 76.7, 47.4, 42.8, 42.4, 34.9, 32.4, 23.4 ppm. HRMS (ESI$^-$): m/z calcd for $C_{21}H_{21}Cl_2N_2O_4$: 435.08839, found: 435.08825 ([M-H]$^-$).

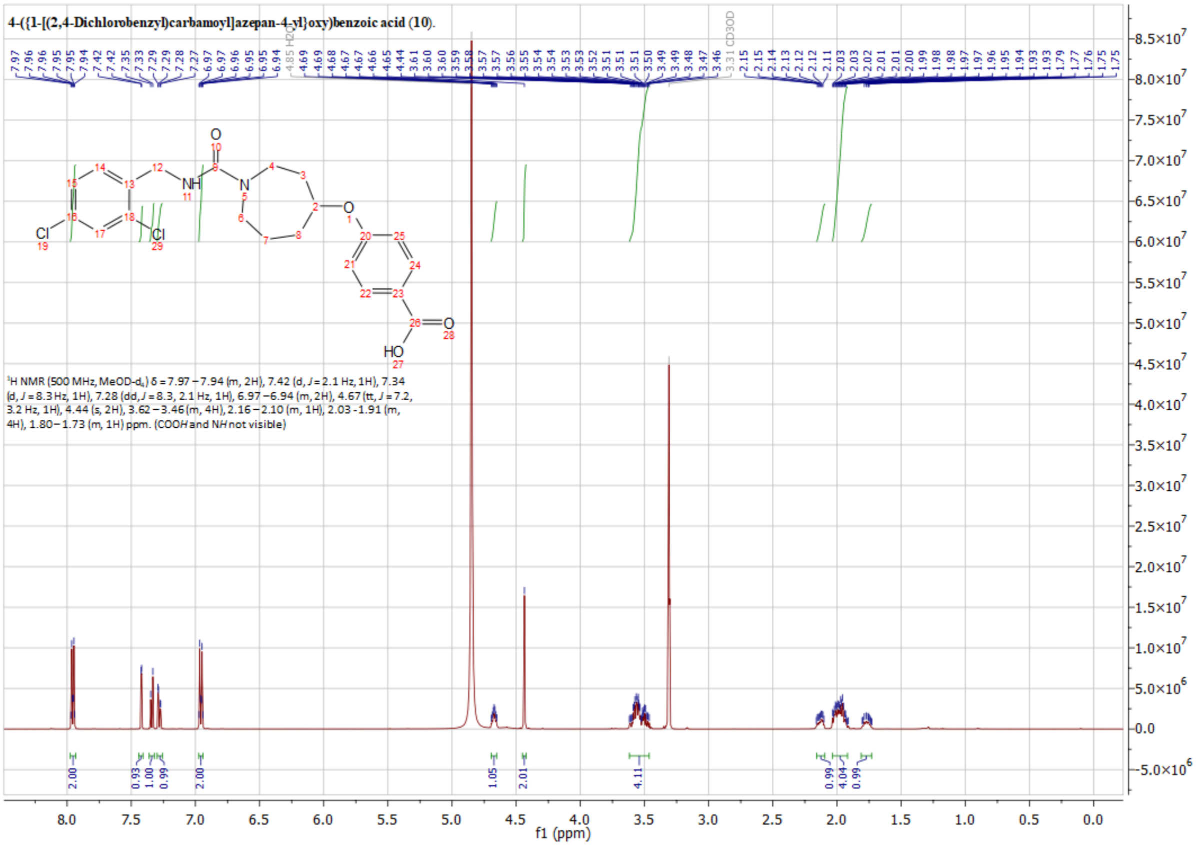

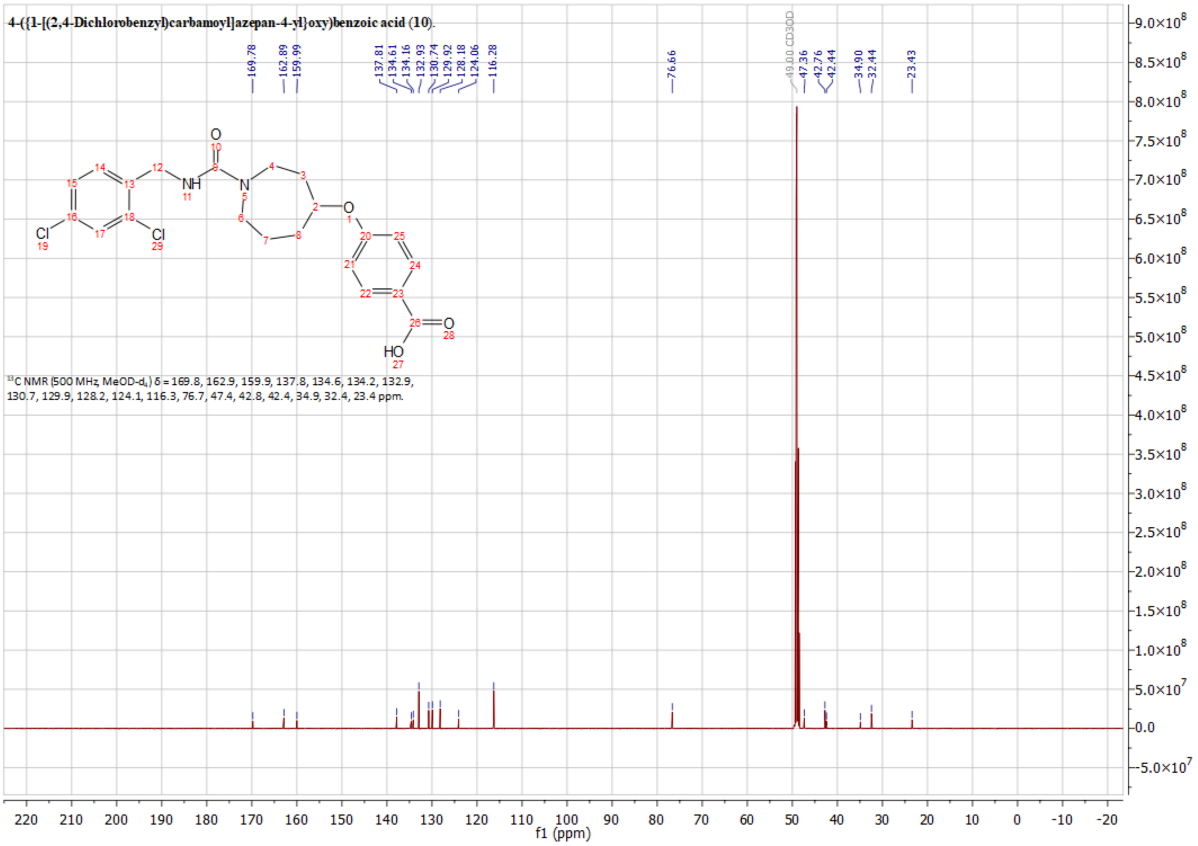
4-({1-[(2,4-Dichlorobenzyl)carbamoyl]azepan-4-yl}oxy)benzoic acid (10).
169.78
162.89
159.99
137.81
134.61
134.16
132.93
130.74
129.92
128.18
124.06
116.28
76.66
49.00 CD3OD
47.36
42.76
42.44
34.90
32.44
23.43
O
NH
N
O
Cl
Cl
HO
13C NMR (500 MHz, MeOD-d4) δ = 169.8, 162.9, 159.9, 137.8, 134.6, 134.2, 132.9, 130.7, 129.9, 128.2, 124.1, 116.3, 76.7, 47.4, 42.8, 42.4, 34.9, 32.4, 23.4 ppm.
f1 (ppm)

4-{1-[3-({[4-(Trifluoromethoxy)phenyl]carbamoyl}amino)phenyl]propyl}benzoic acid (11).

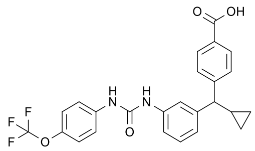

An aqueous NaOH solution (2 M, 10 mL) was added to a solution of **52** (0.26 g, 0.55 mmol, 1.0 eq) in THF (5 mL) and mixed with MeOH until no separation was observed anymore and stirred over night at rt. Then the solvent was removed under reduced pressure and the remaining residue was washed with an aqueous HCl solution (2 M, 50 ml). The crude product was further purified with semi-preparative HPLC (HPLC method 2) and **11** was obtained as colourless crystals (0.24 g, 0.52 mmol, 95%). t_R: 15.97 min (HPLC method 2), purity: 98 % (254 nm). ^{1}H-NMR (300 MHz, DMSO-d$_6$): δ = 12.77 (s, 1H), 8.83 (s, 1H), 8.68 (s, 1H), 7.86 (d, J = 7.9 Hz, 2H), 7.55 (d, J = 8.8 Hz, 2H), 7.42–7.37 (m, 3H), 7.28–7.17 (m, 4H), 6.93 (d, J = 7.6 Hz, 1H), 3.88 (t, J = 7.6 Hz, 1H), 2.03 (quint, J = 7.2 Hz, 2H), 0.84 (t, J = 7.2 Hz, 2H) ppm. ^{13}C-NMR (75 MHz, DMSO-d$_6$): δ = 167.2, 152.4, 150.2, 144.9, 142.5, 139.6, 138.9, 129.5, 128.7, 127.8, 121.9, 121.7, 121.4, 119.4, 117.6, 116.3, 55.3, 27.5, 12.5 ppm. ^{19}F-NMR (282 MHz, DMSO-d$_6$): δ = 57.09 ppm. MS (ESI+): m/z 450.90 ([M+H]$^+$). HRMS (ESI$^+$): m/z calcd for $C_{25}H_{23}F_3N_2O_4$: 459.15262, found: 459.1770 ([M+H]$^+$).

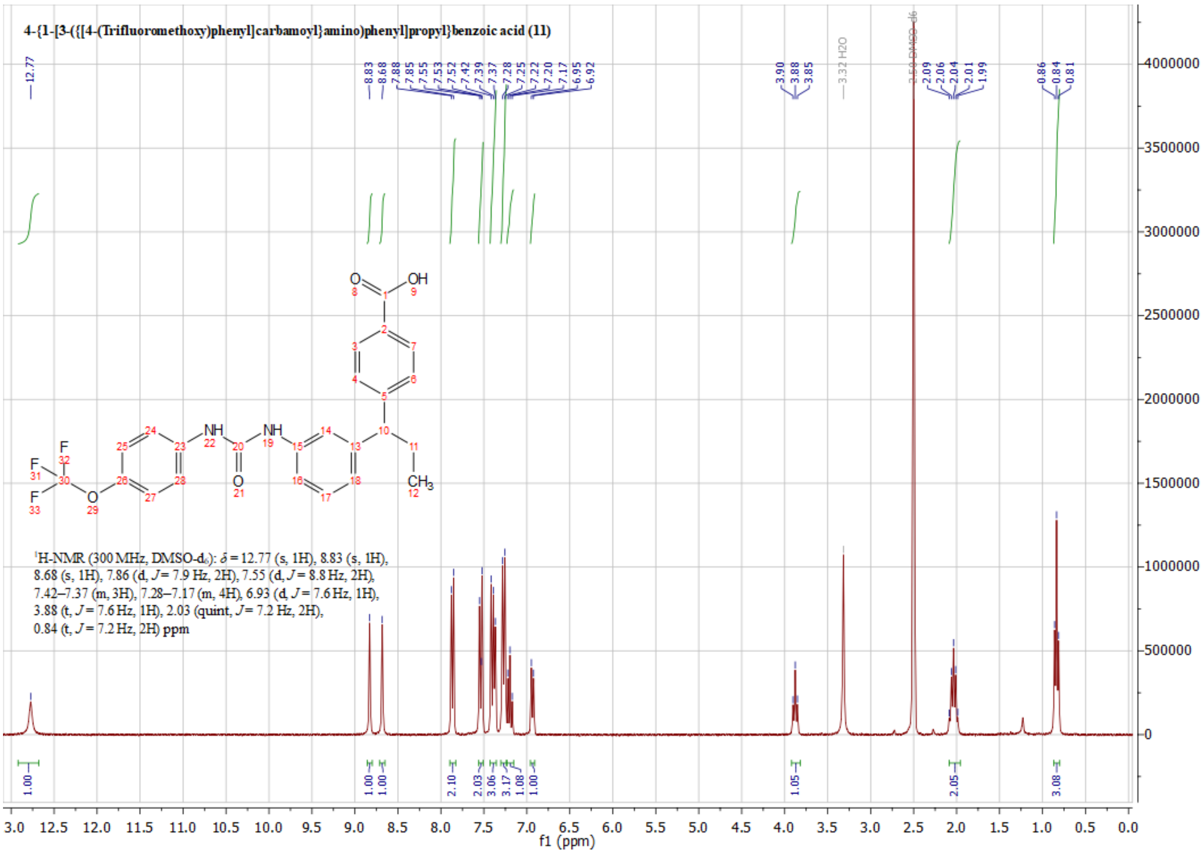

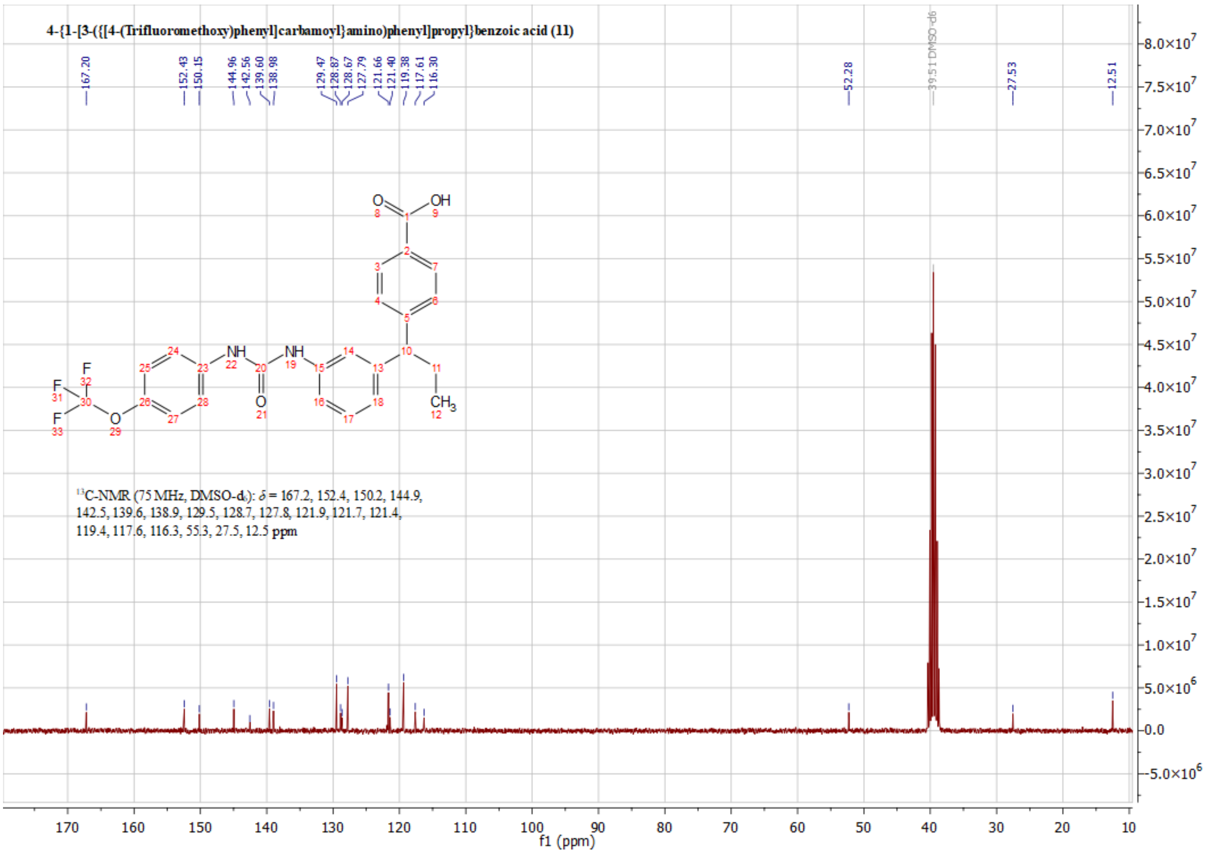
4-{1-[3-({[4-(Trifluoromethoxy)phenyl]carbamoyl}amino)phenyl]propyl}benzoic acid (11)
167.20
152.43
150.15
144.96
142.56
139.60
138.98
129.47
128.87
128.67
127.79
121.66
121.40
119.38
117.61
116.30
52.28
39.51 DMSO-d6
27.53
12.51
13C-NMR (75 MHz, DMSO-d6): δ = 167.2, 152.4, 150.2, 144.9, 142.5, 139.6, 138.9, 129.5, 128.7, 127.8, 121.9, 121.7, 121.4, 119.4, 117.6, 116.3, 55.3, 27.5, 12.5 ppm
f1 (ppm)

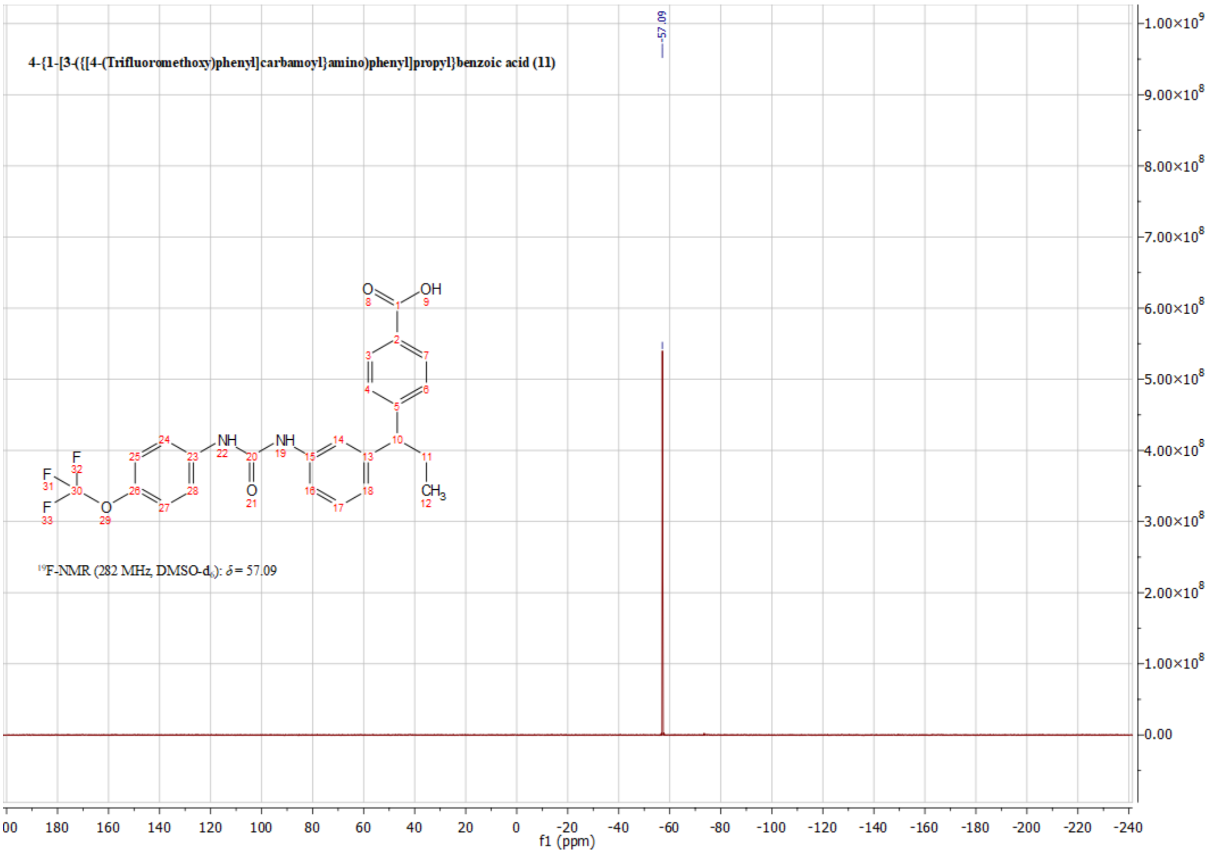
4-{1-[3-({[4-(Trifluoromethoxy)phenyl]carbamoyl}amino)phenyl]propyl}benzoic acid (11)
-57.09
19F-NMR (282 MHz, DMSO-d6): δ = 57.09
f1 (ppm)

3'-{[4-(1-Carboxypent-3-yn-2-yl)phenoxy]methyl}[1,1'-biphenyl]-2-carboxylic acid (12).

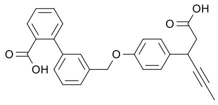

Methyl 3'-{[4-(1-methoxy-1-oxohex-4-yn-3-yl)phenoxy]methyl}[1,1'-biphenyl]-2-carboxylate (**58**, 105 mg, 0.227 mmol, 1 eq) was dissolved in a mixture of THF/H_2O/MeOH (1:2:1, 12 mL). KOH (110 mg, 1.66 mmol, 7 eq) was added and the mixture was stirred overnight at 100 °C. The solvents were removed under reduced pressure and the remaining residue was suspended in aqueous HCl solution (2 M) and extracted with EtOAc (3x). The combined organic phases were washed with brine (1x), dried over $MgSO_4$, filtered and the solvent was removed under reduced pressure. The crude product was further purified by semi-preparative HPLC (HPLC method 1) and **12** was obtained as a colorless solid (73 mg, 0.18 mmol, 74%). t_R: 14.93 min (HPLC method 2), purity: 99% (254 nm). ^{1}H NMR (500 MHz, DMSO-d_6) δ = 12.53 (bs, 2H), 7.74 (dd, J = 7.7, 1.4 Hz, 1H), 7.58 (td, J = 7.5, 1.4 Hz, 1H), 7.46 (td, J = 7.6, 1.3 Hz, 1H), 7.44–7.41 (m, 3H), 7.38 (dd, J = 7.7, 1.3 Hz, 1H), 7.321–7.27 (m, 3H), 6.99 – 6.97 (m. 2H), 5.11 (s, 2H), 3.95 (td, J = 7.6, 2.5 Hz, 1H), 2.60 (d, J = 7.5 Hz, 2H), 1.78 (d, J = 2.4 Hz, 3H) ppm. ^{13}C NMR (126 MHz, DMSO-d_6) δ = 171.8, 169.6, 157.3, 141.0, 140.7, 137.0, 133.5, 132.4, 130.9, 130.5, 129.1, 128.3, 128.2, 127.8, 127.4, 127.4, 126.4, 114.7, 80.6, 78.2, 69.2, 42.8, 32.7, 3.2 ppm. MS (ESI-): m/z 412.80 ([M-H]$^-$). HRMS (ESI$^+$): m/z calcd for $C_{26}H_{23}O_5$: 415.15400, found: 415.1515 ([M+H]$^+$).

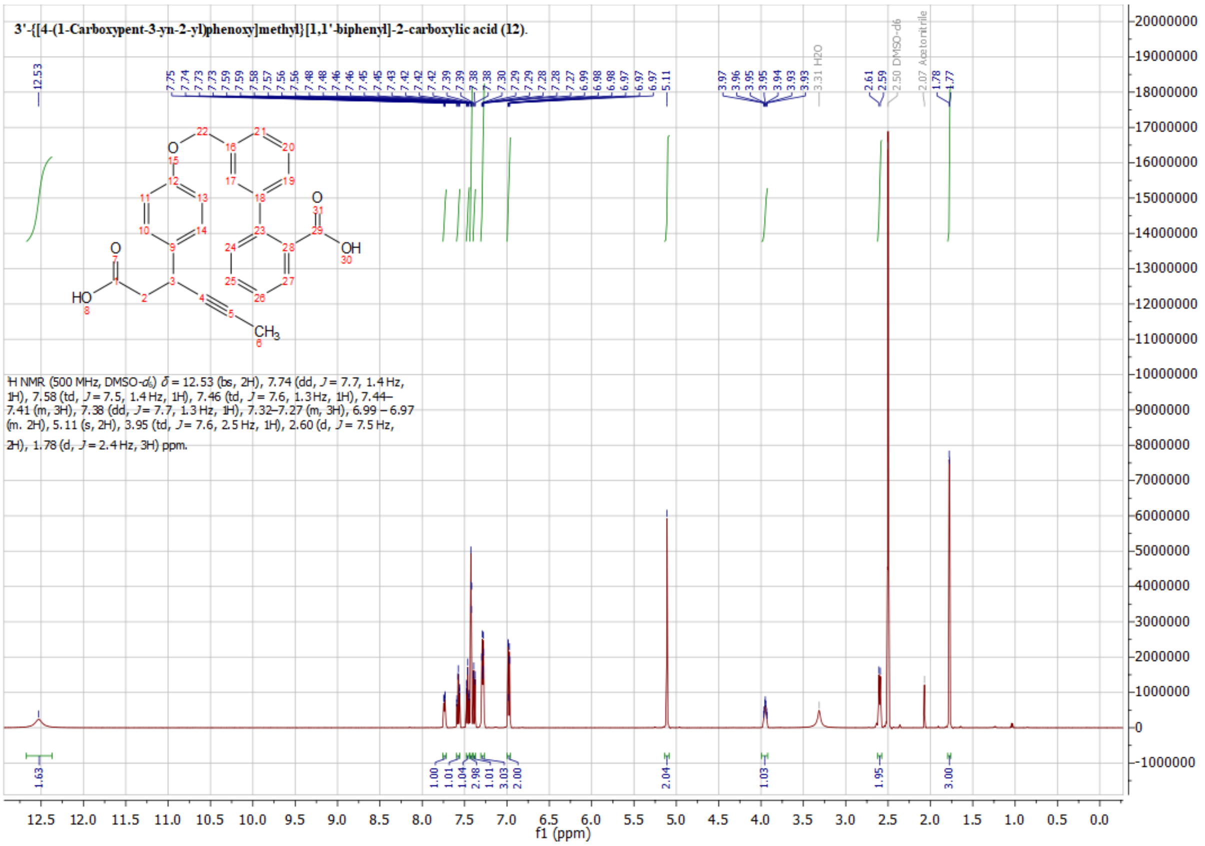

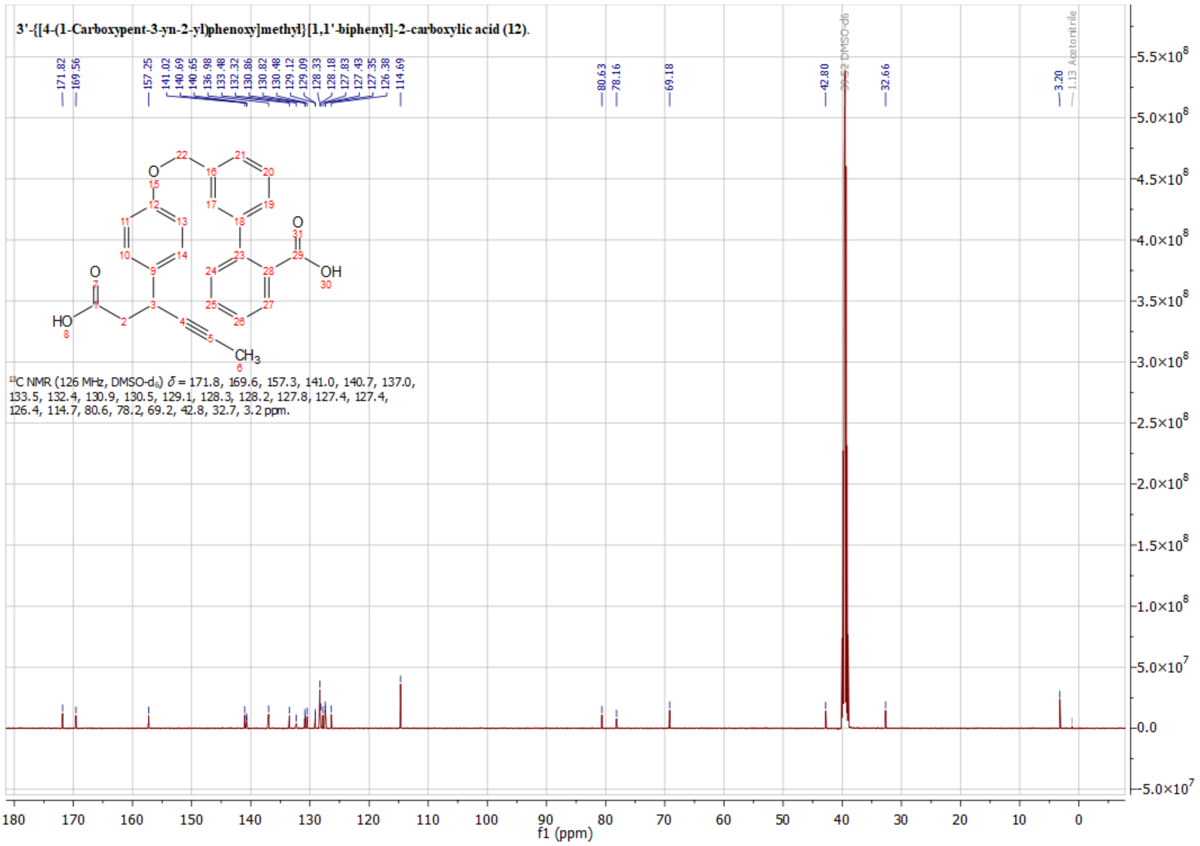
3'-{[4-(1-Carboxypent-3-yn-2-yl)phenoxy]methyl}[1,1'-biphenyl]-2-carboxylic acid (12).
171.82
169.56
157.25
141.02
140.69
140.65
136.98
133.48
132.32
130.86
130.82
130.48
129.12
129.09
128.33
128.18
127.83
127.43
127.35
126.38
114.69
80.63
78.16
69.18
42.80
39.52 DMSO-d6
32.66
3.20
1.13 Acetonitrile
13C NMR (126 MHz, DMSO-d6) δ = 171.8, 169.6, 157.3, 141.0, 140.7, 137.0, 133.5, 132.4, 130.9, 130.5, 129.1, 128.3, 128.2, 127.8, 127.4, 127.4, 126.4, 114.7, 80.6, 78.2, 69.2, 42.8, 32.7, 3.2 ppm.
f1 (ppm)

***N*-(2,6-Dichlorobenzyl)-4-iodobenzamide (15)**.

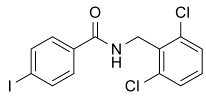

The synthesis was performed according to a modified literature procedure.[20,23] 4-Iodobenzoic acid (0.31 g, 1.2 mmol, 1.0 eq), 1-ethyl-3-(3-dimethylaminopropyl)carbodiimid hydrochloride (0.31 g, 1.6 mmol, 1.3 eq) and DMAP (0.30 g, 2.5 mmol, 2.0 eq) were mixed under Ar with dry and degassed 1,4-dioxane (12 mL). (2,6-Dichlorophenyl)methanamine (0.29 g, 1.6 mmol, 1.3 eq) was added and the resulting suspension was heated to reflux for 2 h. The reaction mixture was allowed to cool to rt and an aqueous HCl solution (10%, 20 mL) was added. The aqueous layer was extracted with EtOAc (3x30 mL), the combined organic layers dried over Na_2SO_4 and the solvent evaporated under reduced pressure to obtain colorless crystals (0.37 g, 0.91 mmol, 75%), which were used without further purification. ^{1}H NMR (400 MHz, DMSO-d_6) δ = 8.73 (t, J = 4.1 Hz, 1H), 7.82 (d, J = 8.2 Hz, 2H), 7.62 (d, J = 8.2 Hz, 2H), 7.48 (d, J = 7.9 Hz, 2H), 7.36 (t, 1H), 4.67 (d, J = 4.4 Hz, 2H) ppm. ^{13}C NMR (101 MHz, DMSO-d_6) δ = 165.8, 137.2, 135.9, 133.6, 133.2, 130.3, 129.6, 128.6, 98.9, 39.8 ppm. MS (ESI+): no molecular ion.

Ethyl 2-[4-(4-hydroxybenzyl)phenoxy]acetate (18).

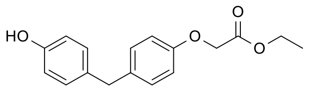

The synthesis was performed according to a modified literature procedure.[24] 4,4‘-Methylenediphenol (0.62 g, 3.1 mmol, 3.0 eq) and potassium carbonate (0.28 g, 2.0 mmol, 2.0 eq) were suspended in dry and degassed DMF. The resulting suspension was slightly pink colored. After the dropwise addition of ethyl-2-bromoacetate (115 µL, 1.02 mmol, 1.0 eq), the reaction mixture was stirred reflux for 3 h. The reaction mixture was allowed to cool to rt and H_2O (30 mL) was added to quench the reaction. The aqueous layer was extracted with EtOAc (3x30 mL). The combined organic layers were washed with brine (3x20 mL), dried over Na_2SO_4 and the solvent was evaporated under reduced pressure. Further purification was performed by CC (DCM/MeOH = 30:1) to obtain **18** as colorless crystals (0.28 g, 0.98 mmol, 96%). ^{1}H NMR (400 MHz, Acetone-d_6) δ = 8.11 (s, 1H), 7.11 (d, J = 8.6 Hz, 2H), 7.02 (d, J = 8.4 Hz, 2H), 6.84 (d, J = 8.6 Hz, 2H), 6.75 (d, J = 8.5 Hz, 2H), 4.66 (s, 2H), 4.19 (q, J = 7.1 Hz, 2H), 3.81 (s, 2H), 1.23 (t, J = 7.1 Hz, 3H) ppm. ^{13}C-NMR (101 MHz, Acetone-d_6) δ = 169.5, 157.4, 156.5, 135.9, 133.3, 130.5, 130.5, 116.0, 115.3, 65.9, 61.4, 40.7, 14.5 ppm. MS (ESI+): m/z 308.97 ([M+Na]$^+$).

***N*-[(2,6-Dichlorophenyl)methylidene]hydroxylamine (19a)**.

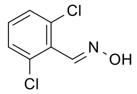

The synthesis was performed according to a literature procedure.[21] 2,6-Dichlorobenzaldehyde (8.21 g, 46.9 mmol, 1.0 eq) was dissolved in EtOH (72 mL). hydroxylamine hydrochloride (3.79 g, 54.0 mmol, 1.15 eq) and NaOH (2.16 g, 54.0 mmol, 1.15 eq) were dissolved in H_2O (12 mL). The solutions were combined and stirred at 90 °C for 18 h. The volume of the reaction mixture was reduced under reduced pressure to precipitate a colorless solid. The solid was filtered and washed serveral times with H_2O before it was dried under reduced pressure. The colorless crystals (8.68 g, 45.9 mol, 97%) were used without further purification. ^{1}H NMR (300 MHz, DMSO-d_6) δ 11.79 (s, 1H), 8.22 (s, 1H), 7.60–7.47 (m, 3H) ppm. ^{13}C NMR (75 MHz, DMSO-d_6) δ = 143.9, 134.0, 131.0, 129.4, 129.0 ppm. MS (ESI+) m/z 190.09 ([M+H]$^+$).

2,6-Dichloro-*N*-hydroxybenzoimidoyl chloride (19b).

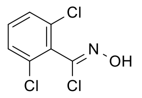

The synthesis was performed according to a modified literature procedure.[21] *N*-[(2,6-Dichlorophenyl)methylidene]hydroxylamine (**19a**, 8.68 g, 45.7 mmol, 1.0 eq) was dissolved in DMF (130.5 mL). *N*-Chlorosuccimide (7.16 g, 52.5 mmol, 1.15 eq) was added and the mixture was stirred at rt for 18 h. H_2O was added and extracted with EtOAc (3x30 mL). The combined organic layers were dried over Na_2SO_4, and the solvent was evaporated under reduced pressure to obtain **19b** as yellow crystals (9.41 g, 42.2 mol, 92%), which were used without further purification. ^{1}H NMR (300 MHz, DMSO-d_6) δ = 12.67 (s, 1H), 7.66–7.54 (m, 3H) ppm. ^{13}C NMR (75 MHz, DMSO-d_6) δ = 134.4, 132.8, 131.8, 128.8, 128.7 ppm. MS (ESI+) no molecular ion.

Methyl 3-(2,6-dichlorophenyl)-5-isopropylisoxazole-4-carboxylate (19c).

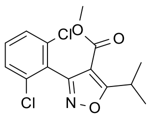

The synthesis was performed according to a modified literature procedure[21] under inert atmosphere. Methyl isobutyrylacetate (685 µL, 4.66 mmol, 1.0 eq) was added to dry and degassed THF (12 mL). After 5 minutes the solution was treated under stirring with a solution of sodium methoxide in MeOH (1.08 mL, 4.70 mmol, 1.01 eq). 2,6-Dichloro-*N*-hydroxybenzoimidoyl chloride (**19b**, 1.04g, 4.66 mmol, 1.0 eq) was solved in degassed THF (12 mL) and added after 40 minutes to the solution of methyl isobutyrylacetate. The reaction mixture was stirred at rt for 18 h. H_2O was added to quench the reaction, and the mixture was extracted with EtOAc (3x30 mL). The combined organic layers were dried over Na_2SO_4 and the solvent was evaporated under reduced pressure. The crude product was purified by CC (hexane/EtOAc = 20:1) to obtain **19c** as a yellow crystals (253 mg, 0.808 mmol, 17%). ^{1}H NMR (300 MHz, $CDCl_3$) δ = 7.33–7.28 (m, 2H), 7.26–7.17 (m, 1H), 3.78 (hept, J = 7.0 Hz, 1H), 3.57 (s, 3H), 1.34 (d, J = 7.0 Hz, 6H) ppm. ^{13}C NMR (75 MHz, $CDCl_3$) δ = 183.3, 161.7, 158.7, 135.5, 131.1, 128.4, 127.9, 107.5, 51.8, 27.9, 20.3 ppm. MS (ESI+) *m/z* 314.07 ([M+H]$^+$).

[3-(2,6-Dichlorophenyl)-5-isopropylisoxazol-4-yl]methanol (19).

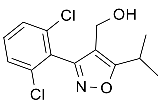

The synthesis was performed according to a modified literature procedure.[21] Methyl 3-(2,6-dichlorophenyl)-5-isopropylisoxazole-4-carboxylate (**19c**, 0.1 g, 0.3 mmol, 1 eq) was dissolved in dry THF (5 mL), and the solution was cooled to 0°C. A solution of diisobutylaluminium hydride in toluene (1 M, 0.32 µL, 0.32 mmol, 1.0 eq) was slowly (50 µL/2 min.) added. The reaction mixture was allowed to warm to rt, before it was heated to 80°C under reflux and stirred for 24 h. The reaction mixture was then cooled on ice to 0°C again and quenched with MeOH (5 mL) and an aqueous NaOH solution (2 M, 20 mL). The suspension was extracted with EtOAc (3x30 mL) and the combined organic layers were wash with brine, dried over Na_2SO_4 and the solvent was evaporated under reduced pressure. The product was purified by CC (hexane/EtOAc = 3:1) to obtain **19** as light yellow crystals (0.06 g, 0.2 mmol, 65%). ^{1}H NMR (400 MHz, $CDCl_3$) δ = 7.34–7.29 (m, 2H), 7.25–7.14 (m, 1H), 4.22 (d, J = 2.5 Hz, 2H), 3.22 (hept, J = 7.0 Hz, 1H), 1.31 (d, J = 7.0 Hz, 6H) ppm. ^{13}C NMR (101 MHz, $CDCl_3$) δ 176.3, 159.1, 135.8, 131.4, 128.2, 127.8, 112.70, 53.7, 27.0, 21.0 ppm. MS (ESI+) *m/z* 286.16 ([M+H]$^+$).

Ethyl 2-[4-(4-{[3-(2,6-dichlorophenyl)-5-isopropylisoxazol-4-yl]methoxy}benzyl)phenoxy]acetate (20).

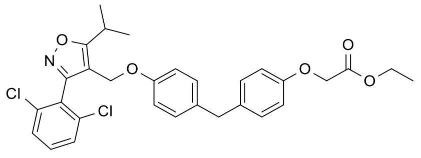

The synthesis was performed according to a modified literature procedure.[21] [3-(2,6-Dichlorophenyl)-5-isopropylisoxazol-4-yl]methanol (**19**, 0.25 g, 0.86 mmol, 1.0 eq), Ethyl 2-[4-(4-hydroxybenzyl)phenoxy]acetate (**18**, 0.25 g, 0.86 mmol, 1.0 eq) and triphenylphosphine (0.23 g, 0.86 mmol, 1.0 eq) were mixed under Ar in dry and degassed DCM (17 mL). Diisopropyl azodicarboxylate (180 µL, 0.86 mmol, 1.0 eq) was added dropwise. The reaction mixture was stirred at rt for 2 h. The reaction was quenched by evaporation of the solvent. The product was purified by CC (hexane/EtOAc = = 93.8:6.2 → 50:50) to obtain **20** as colorless crystals (0.23 g, 0.42 mmol, 47%). ^{1}H NMR (400 MHz, Acetone-d_6) δ = 7.55–7.45 (m, 3H), 7.09 (d, J = 8.7 Hz, 2H), 7.05 (d, J = 8.6 Hz, 2H), 6.83 (d, J = 8.7 Hz, 2H), 6.73 (d, J = 8.7 Hz, 2H), 4.82 (s, 2H), 4.65 (s, 2H), 4.19 (q, J = 7.1 Hz, 2H), 3.80 (s, 2H), 3.44 (hept, J = 7.0 Hz, 1H), 1.37 (d, J = 7.0 Hz, 6H), 1.23 (t, J = 7.1 Hz, 3H) ppm. ^{13}C NMR (101 MHz, Acetone-d_6) δ = 176.8, 169.4, 159.9, 157.6, 157.4, 136.2, 135.5, 135.3, 132.7, 130.5, 130.4, 129.1, 129.0, 115.6, 115.3, 110.8, 65.8, 61.4, 60.1, 40.6, 27.4, 21.1, 14.5 ppm. MS (ESI+): *m/z* 554.00 ([M+H]$^+$).

Ethyl-4-hydroxybenzoat (21a).

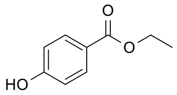

The synthesis was performed according to a modified literature procedure.[21,25] 4-Hydroxybenzoic acid (1.00 g, 7.24 mmol, 1.0 eq) was dissolved in EtOH (21 mL). Concentrated H_2SO_4 (2.00 mL, 36.2 mmol, 5.0 eq) was added dropwise. The reaction mixture was refluxed for 3 h. After cooling to rt, EtOH was evaporated under reduced pressure. Saturated aqueous $NaHCO_3$ solution was added until it stopped bubbling and the resulting aqueous phase was extracted with EtOAc (3x30 mL). The combined organic layers were dried over Na_2SO_4 and the solvent was evaporated under reduced pressure to obtain **21a** as colorless crystals (1.15 g, 6.93 mmol, 96 %). The product was used without further purification. ^{1}H NMR (400 MHz, Acetone-d_6) δ = 9.18 (s, 1H), 7.89 (dd, 2H), 6.93 (dd, 2H), 4.28 (q, J = 7.1 Hz, 2H), 1.32 (t, J = 7.1 Hz, 3H) ppm. ^{13}C NMR (101 MHz, Acetone-d_6) δ = 166.6, 162.6, 132.3, 122.6, 116.0, 60.9, 14.6 ppm. MS (ESI+) *m/z* 165.30 ([M+H]$^+$).

Ethyl-4-{[3-(2,6-dichlorophenyl)-5-isopropylisoxazol-4-yl]methoxy}benzoate (22).

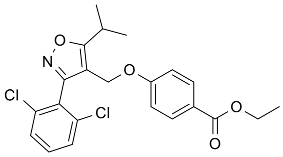

The synthesis was performed according to a modified literature procedure.[21] [3-(2,6-Dichlorophenyl)-5-isopropylisoxazol-4-yl]methanol (**19**, 0.1 g, 0.4 mmol, 1 eq), ethyl-4-hydroxybenzoat (**21a**, 0.06 g, 0.4 mmol, 1 eq) and triphenylphosphine (0.09 g, 0.4 mmol, 1 eq) were dissolved in dry and degassed DCM (7 mL). Diisopropyl azodicarboxylate (0.07 µL, 0.4 mmol, 1 eq) was added dropwise. The reaction mixture was stirred at rt for 4 h and quenched by evaporation of the solvent. The crude product was purified by CC (hexane/EtOAc = 97.7:2.3 → 81.1:18.9) to obtain **22** as colorless oil (0.08 g, 0.2 mmol, 53%). ^{1}H NMR (400 MHz, Acetone-d_6) δ = 7.89 (d, J = 8.9 Hz, 2H), 7.57–7.47 (m, 3H), 6.91 (d, J = 8.9 Hz, 2H), 4.98 (s, 2H), 4.28 (q, J = 7.1 Hz, 2H), 3.50 (hept, J = 7.0 Hz, 1H), 1.40 (d, J = 7.0 Hz, 6H), 1.32 (t, J = 7.1 Hz, 3H) ppm. ^{13}C NMR (101 MHz, Acetone-d_6) δ = 177.0, 166.2, 162.9, 159.9, 136.2, 132.8, 132.0, 129.2, 128.8, 124.2, 115.2, 110.2, 61.1, 60.2, 27.5, 21.1, 14.6 ppm. MS (ESI+) *m/z* 433.87 ([M+H]$^+$).

Ethyl 2-(4-[{3-(2,6-dichlorophenyl)-5-isopropylisoxazol-4-yl]methoxy}phenyl)acetate (23).

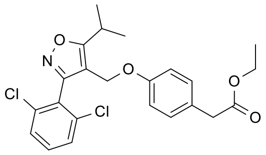

The synthesis was performed according to a modified literature procedure.[21] [3-(2,6-dichlorophenyl)-5-isopropylisoxazol-4-yl]methanol (**19**, 0.1 g, 0.4 mmol, 1 eq), ethyl 4-hydroxyphenylacetate (**21b**, 0.06 g, 0.4 mmol, 1 eq) and triphenylphosphine (0.09 g, 0.4 mmol, 1 eq) were dissolved in dry and degassed DCM (7 mL). Diisopropyl azodicarboxylate (70 µL, 0.35 mmol, 1.0 eq) was added dropwise. The reaction mixture was stirred at rt for 20 h and quenched by evaporation of the solvent. The crude product was purified by CC (hexane/EtOAc = 97.7:2.3 → 81.1:18.9) to obtain **23** as colorless crystals (0.06 g, 0.1 mmol, 39%). ^{1}H NMR (400 MHz, Acetone-*d*$_6$) δ = 7.57-7.46 (m, 3H), 7.14 (d, *J* = 8.7 Hz, 2H), 6.78 (d, *J* = 8.7 Hz, 2H), 4.85 (s, 2H), 4.07 (q, *J* = 7.1 Hz, 2H), 3.51 (s, 2H), 3.47 (hept, *J* = 7.0 Hz, 1H), 1.39 (d, *J* = 7.0 Hz, 6H), 1.18 (t, *J* = 7.1 Hz, 3H) ppm. ^{13}C NMR (101 MHz, Acetone-*d*$_6$) δ = 176.8, 171.9, 159.9, 158.2, 136.2, 132.7, 131.1, 129.1, 128.9, 128.2, 115.5, 110.7, 60.9, 60.0, 40.6, 27.4, 21.1, 14.5 ppm. MS (ESI+) *m/z* 496.84 ([M+Na]$^+$).

(2,4,6-Trichlorophenyl)methanamine (26).

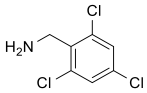

26 was synthesized according to a modified literature procedure for the reduction of nitriles[26]. A solution of $LiAlH_4$ (95 mg, 2.5 mmol, 1.0 eq) in THF (5 mL) was cooled to 0 °C and 2,4,6-trichlorobenzonitrile (0.52 mg, 2.5 mmol, 1.0 eq) dissolved in THF (0.5 mL) was added dropwise. After stirring for 45 min at 0 °C, H_2O (0.1 mL), NaOH (aqu., 2 m, 0.2 mL) and again H_2O (0.25 mL) was added dropwise. The mixture was diluted with DCM and the aqueous layer was extracted with DCM (2x15 mL). The combined organic layers were washed with brine, dried over Na_2SO_4, filtrated and the solvent removed under reduced pressure. Purification was performed by CC (isohexane + 1% NEt_3 → isohexane/EtOAc 9:1 + 1% NEt_3, R_f = 0.13 for isohexane/EtOAc 9:1 + 1% NEt_3) giving the colourless solid product **13** (0.25 g, 1.2 mmol, 48%). ^{1}H-NMR (400 MHz, $CDCl_3$): δ = 7.33 (s, 2H), 4.07 (s, 2H), 1.51 (s, 2H). ^{13}C NMR (101 MHz, $CDCl_3$): δ = 137.6, 135.8, 133.6; 128.5; 41.7 ppm. MS (APCI+): *m/z* 209.7 ([M+H]$^+$).

***tert*-Butyl (2,4,6-trichlorobenzyl)carbamate (27).**

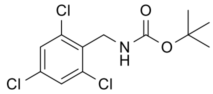

Synthesis was performed according to a standard procedure for the Boc protection of amines.[27,28] **26** (73 mg, 0.35 mmol, 1.0 eq), NEt_3 (96 µL, 0.70 mmol, 2.0 eq) and Boc_2O (0.14 mL, 2.5 m in DCM, 0.35 mmol, 1.0 eq) were dissolved in DCM (1.5 mL) and stirred at rt for 18 h. H_2O was added, the layers separated, and the aqueous layer extracted with DCM. The combined organic layers were dried over Na_2SO_4, filtered and the solvent removed under reduced pressure. The product was obtained as yellowish crystals (105 mg, 0.34 mmol, 97%) which were used without further purification. ^{1}H NMR (400 MHz, $CDCl_3$): δ = 7.33 (s, 2H), 4.84 (bs, 1H), 4.59 (d, *J* = 6.0 Hz, 2H), 1.44 (s, 9H) ppm. ^{13}C NMR (101 MHz, $CDCl_3$): δ = 155.3; 136.7; 134.5, 133.1; 128.6; 80.0; 39.9; 28.5 ppm. MS (APCI−): *m/z* 307.6 ([M–H]$^-$).

***tert*-Butyl [*cis*-4-hydroxycyclohexyl]carbamate (30)**.

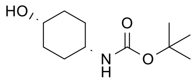

Synthesis was performed according to a standard procedure for the Boc protection of amines.[27,28] *cis*-4-Aminocyclohexanol hydrochloride (0.30 g, 2.0 mmol, 1.0 eq), NEt_3 (0.55 mL, 4.0 mmol, 2.0 eq) and Boc_2O (0.80 mL, 2.5 M in DCM, 2.0 mmol, 1.0 eq) were dissolved in DCM (3.5 mL) and stirred at rt for 4 days until full conversion. H_2O was added, the layers separated, and the aqueous layer extracted with DCM. The combined organic layers were dried over $MgSO_4$, filtered and the solvent removed under reduced pressure. Product **30** was obtained as colourless crystals (0.40 g, 1.9 mmol, 94%) which was used without further purification. NMR spectra matched the ones found in the literature.[29] MS (APCI+): *m/z* 215.8 ($[M+H]^+$).

Methyl 3-(4-{[*trans*-4-(*tert*-butoxycarbonylamino)cyclohexyl]oxy}phenyl)propanoate (31).

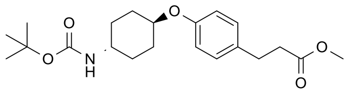

Synthesis was performed according to a similar literature procedure.[30,31] Methyl 3-(4-hydroxyphenyl)propionate (0.18 g, 1.0 mmol, 1.0 eq) and PPh_3 (0.29 g, 1.1 mmol, 1.1 eq) were dissolved in THF (2 mL), and the solution was cooled to 0 °C. A solution of **30** (0.22 g, 1.0 mmol, 1.0 eq) and DEAD (0.5 mL, 40% in toluene, 1.1 mmol, 1.1 eq) in THF (1.5 mL) was added dropwise within 20 min before the mixture was allowed to warm to rt within 18 h. When no further conversion was observed by TLC, H_2O was added, and the aqueous layer extracted with DCM. The combined organic layers were washed with brine, dried over Na_2SO_4, filtered and the solvent was removed under reduced pressure. Purification was performed by CC (isohexane → isohexane/EtOAc = 19:1) giving **31** as colourless crystals (0.16 g, 0.42 mmol, 42%). R_f = 0.69 (isohexane/EtOAc 7:3 + 1% NEt_3). ^1H NMR (400 MHz, $CDCl_3$): δ = 7.08 (d, J = 8.6 Hz, 2H), 6.80 (d, J = 8.6 Hz, 2H), 4.41 (s, 1H), 4.18–4.06 (m, 1H), 3.66 (s, 3H), 3.50 (s, 1H), 2.87 (t, J = 7.8 Hz, 2H), 2.59 (t, J = 7.8 Hz, 2H), 2.15–2.03 (m, 4H), 1.62–1.47 (m, 2H), 1.44 (s, 9H), 1.31–1.16 (m, 2H) ppm. ^{13}C NMR (101 MHz, $CDCl_3$): δ = 173.6, 156.2, 155.4, 133.0, 129.4, 116.3, 79.5, 75.3, 51.7, 48.9, 36.1, 31.0, 30.4, 30.2, 28.6 ppm. MS (APCI+): *m/z* 277.8 ($[M+H–C_5H_6O_2]^+$).

Methyl 3-(4-{[*trans*-4-{[(4-*tert*-butylphenyl)carbamoyl]amino}cyclohexyl]oxy}phenyl)propanoate (32).

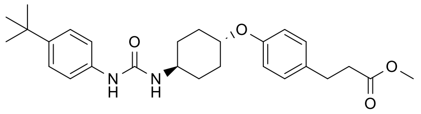

Synthesis was performed according to a modified literature procedure.[32] **31** (46 mg, 0.12 mmol, 1.0 eq) was dissolved in DCM (1.5 mL), DIPEA (25 µL, 0.15 mmol, 1.2 eq) was added, and the mixture cooled to −35 °C. SiH_2I_2 (24 µL, 0.24 mmol, 2.0 eq) was added under exclusion of light and the mixture rewarmed to −5 °C. After 2 h, upon full conversion of the starting material as indicated by TLC, the reaction was cooled to −50 °C and 4-*tert*-butylaniline (18 mg, 0.12 mmol, 1.0 eq) was added in DCM (0.75 mL). After 45 min of stirring while warming to −35 °C, further DIPEA (16 µL, 0.096 mmol, 1.2 eq) was added. The mixture was warmed to rt and stirred for 17 h. The solvent was removed under reduced pressure and the residue dissolved in EtOAc. The organic layer was washed with H_2O (3x) and brine, dried over $MgSO_4$, filtered and the solvent was removed under reduced pressure. The crude product was purified by CC (isohexane/EtOAc = 9:1 → 3:7) to give **32** as colourless crystals (30 mg, 0.066 mmol, 54%). R_f = 0.49 (isohexane/EtOAc = 7:3). ^1H NMR (500 MHz, $CDCl_3$): δ = 7.29 (d, J = 8.7 Hz, 2H), 7.19 (d, J = 8.7 Hz, 2H), 7.06 (d, J = 8.7 Hz, 2H), 7.01 (s, 1H), 6.78 (d, J = 8.7 Hz, 2H), 5.18 (d, J = 7.9 Hz, 1H), 4.12–4.03 (m, 1H), 3.77–3.67 (m, 1H), 3.66 (s, 3H), 2.87 (t, J = 7.8 Hz, 2H), 2.59 (t, J = 7.8 Hz, 2H), 2.11–2.03 (m, 4H), 1.57–1.45 (m, 2H), 1.28 (s, 9H), 1.26–1.19 (m, 2H) ppm. ^{13}C NMR (126 Hz, $CDCl_3$): δ = 173.6, 156.2, 155.8, 146.8, 136.1, 132.9, 129.4, 126.2, 120.9, 116.2, 75.2, 51.7, 48.3, 36.1, 34.4, 31.5, 31.0, 30.3, 30.2 ppm. MS (APCI+): *m/z* 452.6 ($[M+H]^+$).

***tert*-Butyl 4-[4-(3-methoxy-3-oxopropyl)phenoxy]piperidine-1-carboxylate (34)**.

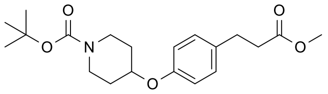

Synthesis was performed according to a modified literature procedure.[30,31] Methyl 3-(4-hydroxyphenyl)propionate (0.18 g, 1.0 mmol, 1.0 eq) and PPh_3 (0.29 g, 1.1 mmol, 1.1 eq) were dissolved in THF (2 mL), and the solution was cooled to 0 °C. A solution of *tert*-butyl 4-hydroxypiperidine-1-carboxylate (0.20 g, 1.0 mmol, 1.0 eq) and DEAD (0.5 mL, 40% in toluene, 1.1 mmol, 1.1 eq) in THF (1.5 mL) was added dropwise within 20 min before the mixture was allowed to warm to rt and stirred for 23 h. H_2O was added, and the aqueous layer extracted with DCM. The combined organic layers were washed with brine, dried over Na_2SO_4, filtered and the solvent was removed under reduced pressure. Purification by CC (isohexane → isohexane/EtOAc 3:2) and recrystallization from isohexane gave product **34** as colourless crystals (0.17 g, 0.46 mmol, 46%). NMR spectra match the ones found in the literature.[30] MS (APCI+): *m/z* 363.7 ($[M+H]^+$).

***tert*-Butyl 4-[4-(3-methoxy-3-oxopropyl)phenoxy]azepane-1-carboxylate (35)**.

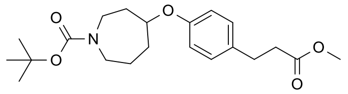

Synthesis was performed according to a similar literature procedure[30,31]. Methyl 3-(4-hydroxyphenyl)propionate (0.18 g, 1.0 mmol, 1.0 eq) and PPh_3 (0.29 g, 1.1 mmol, 1.1 eq) were dissolved in THF (2 mL), and the solution was cooled to 0 °C. A solution of *tert*-butyl 4-hydroxyazepane-1-carboxylate (0.22 g, 1.0 mmol, 1.0 eq) and DEAD (0.5 mL, 40% in toluene, 1.1 mmol, 1.1 eq) in THF (1.5 mL) was added dropwise within 20 min before the mixture was allowed to warm to rt within 19 h. When no further conversion was observed by TLC, H_2O was added, and the aqueous layer extracted with DCM. The combined organic layers were washed with brine, dried over Na_2SO_4, filtered and the solvent was removed under reduced pressure. Purification was performed by CC (isohexane → isohexane/EtOAc 9:1) giving the product **35** as colourless oil (48 mg, 0.13 mmol, 13%). R_f = 0.82 (isohexane/EtOAc = 3:2). ^{1}H NMR (400 MHz, $CDCl_3$): δ = 7.09 (d, J = 8.5 Hz, 2H), 6.79 (d, J = 8.5 Hz, 2H), 4.47–4.30 (m, 1H), 3.66 (s, 3H), 3.64–3.36 (m, 3H), 3.36–3.20 (m, 1H), 2.88 (t, J = 7.8 Hz, 2H), 2.59 (t, J = 7.8 Hz, 2H), 2.12–1.98 (m, 1H), 1.98–1.83 (m, 4H), 1.70–1.60 (m, 1H), 1.47 (s, 9H) ppm. ^{13}C NMR (101 MHz, $CDCl_3$): δ = 173.6, 156.0, 155.7, 132.9, 129.5, 115.7, 79.5, 75.9, 75.7, 51.7, 46.6, 46.1, 41.9, 41.5, 36.1, 34.3, 34.0, 32.0, 31.3, 30.2, 28.7, 22.8, 22.3 ppm. MS (APCI+): *m/z* 377.8 ($[M+H]^+$).

Methyl 3-[4-(piperidin-4-yloxy)phenyl]propanoate (36).

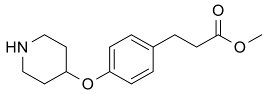

36 was synthesized according to a literature procedure.[27,30] Reaction was not performed under inert atmosphere. A solution of **34** (94 mg, 0.26 mmol, 1.0 eq) in DCM (1.4 mL) was cooled to 0 °C and TFA (0.20 mL, 2.6 mmol, 10 eq) was added dropwise. The mixture was stirred and allowed to warm to rt for 19 h. Upon full conversion, the solvent was removed under reduced pressure, the residue again dissolved in DCM and washed with a saturated aqueous $NaHCO_3$ solution. The aqueous layer was extracted with DCM, the combined organic layers were dried over Na_2SO_4, filtered and the solvent removed under reduced pressure. Product **36** was obtained as yellowish oil (68 mg, 0.26 mmol, quant.) and used without further purification. ^{1}H NMR (400 MHz, $CDCl_3$): δ = 7.11 (d, J = 8.6 Hz, 2H), 6.83 (d, J = 8.6 Hz, 2H), 5.38 (bs, 1H), 4.50–4.41 (m, 1H), 3.66 (s, 3H), 3.29–3.19 (m, 2H), 2.98–2.91 (m, 2H), 2.89 (t, J = 7.8 Hz, 2H), 2.59 (t, J = 7.8 Hz, 2H), 2.13–2.02 (m, 2H), 1.93–1.82 (m, 2H) ppm. ^{13}C NMR (101 MHz, $CDCl_3$): δ = 173.5, 155.4, 133.5, 129.6, 116.3, 70.8, 51.7, 42.0, 36.0, 30.2, 29.8 ppm. NMR values deviate from those found in literature.[30] This is considered to be a result of amine protonation in the literature spectra. MS (APCI+): *m/z* 263.8 ($[M+H]^+$).

Methyl 3-[4-(azepan-4-yloxy)phenyl]propanoate (37).

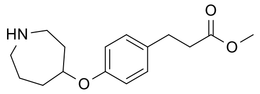

37 was synthesized according to a similar literature procedure and not under inert atmosphere.[27,30] A solution of **35** (50 mg, 0.13 mmol, 1.0 eq) in DCM (0.7 mL) was cooled to 0 °C and TFA (0.10 mL, 1.3 mmol, 10 eq) was added dropwise. The mixture was stirred and allowed to warm to rt within 14 h. Upon full conversion, the solvent was removed under reduced pressure, the residue again dissolved in DCM and washed with a solution of $NaHCO_3$ (sat. aqu.). The aqueous layer was extracted with DCM, the combined organic layers were dried over Na_2SO_4, filtered and the solvent removed under reduced pressure. Product **37** was obtained as colourless oil (35 mg, 0.13 mmol, 96%) and used without further purification. ^{1}H NMR (400 MHz, $CDCl_3$): δ = 7.11 (d, J = 8.6 Hz, 2H), 6.81 (d, J = 8.6 Hz, 2H), 4.68–4.59 (m, 1H), 3.67 (s, 3H), 3.44–3.32 (m, 1H), 3.28–3.14 (m, 3H), 2.89 (t, J = 7.7 Hz, 2H), 2.59 (t, J = 7.7 Hz, 2H), 2.30–1.92 (m, 5H), 1.88–1.75 (m, 1H) ppm. ^{13}C NMR (101 MHz, $CDCl_3$): δ = 173.5, 155.2, 133.6, 129.6, 116.3, 73.2, 51.7, 46.1, 40.1, 36.0, 31.6, 30.2, 30.1, 19.7 ppm. MS (APCI+): *m/z* 277.8 ($[M+H]^+$).

Methyl 3-[4-({1-[(2,4,6-trichlorobenzyl)carbamoyl]piperidin-4-yl}oxy)phenyl]propanoate (38).

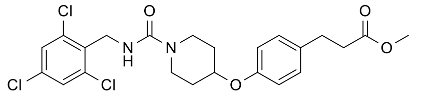

Synthesis was performed according to a modified literature procedure.[32] **27** (50 mg, 0.16 mmol, 1.0 eq) was dissolved in DCM (1.2 mL), DIPEA (33 µL, 0.19 mmol, 1.2 eq) was added, and the mixture cooled to −35 °C. SiH_2I_2 (19 µL, 0.19 mmol, 1.2 eq) was added under exclusion of light and the mixture rewarmed to −5 °C within 3 h. Upon full conversion of the starting material as indicated by TLC, the reaction was cooled to −50 °C and **36** (51 mg, 0.19 mmol, 1.2 eq) was added in DCM (1.5 mL). After 45 min of stirring while warming to −35 °C, further DIPEA (33 µL, 0.19 mmol, 1.2 eq) was added. The mixture was warmed to rt within 23 h. The solvent was removed under reduced pressure and the residue dissolved in EtOAc. The organic layer was washed with H_2O (3x) and brine, dried over $MgSO_4$, filtered and the solvent was removed under reduced pressure. The crude product was purified by CC (isohexane/EtOAc 3:2 +1% NEt_3,) to give product **38** (19 mg, 0.038 mmol, 24%). R_f = 0.19 (isohexane/EtOAc = 3:2). ^{1}H NMR (400 MHz, $CDCl_3$): δ = 7.34 (s, 2H), 7.09 (d, J = 8.5 Hz, 2H), 6.81 (d, J = 8.5 Hz, 2H), 4.89 (t, J = 5.5 Hz, 1H), 4.67 (d, J = 5.5 Hz, 2H), 4.48–4.38 (m, 1H), 3.66 (s, 3H), 3.64–3.54 (m, 2H), 3.35–3.24 (m, 2H), 2.88 (t, J = 7.8 Hz, 2H), 2.59 (t, J = 7.8 Hz, 2H), 1.96–1.84 (m, 2H), 1.83–1.71 (m, 2H) ppm. ^{13}C NMR (101 MHz, $CDCl_3$): δ = 173.5, 157.0, 155.7, 136.7, 134.3, 133.5, 133.3, 129.5, 128.6, 116.3, 71.9, 51.7, 41.1, 40.4, 36.0, 30.4, 30.2 ppm. MS (APCI+) *m/z* 498.5 ($[M+H]^+$).

Methyl 3-[4-({1-[(2,4,6-trichlorobenzyl)carbamoyl]azepan-4-yl}oxy)phenyl]propanoate (39).

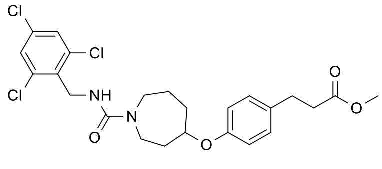

Synthesis was performed according to a modified literature procedure.[32] **27** (37 mg, 0.12 mmol, 1.0 eq) was dissolved in DCM (1.0 mL), DIPEA (24 µL, 0.14 mmol, 1.2 eq) was added, and the mixture cooled to −35 °C. SiH_2I_2 (14 µL, 0.14 mmol, 1.2 eq) was added under exclusion of light and the mixture rewarmed to −5 °C. After 2 h, upon full conversion of the starting material as indicated by TLC, the reaction was cooled to −50 °C and **37** (33 mg, 0.12 mmol, 1.0 eq) was added in DCM (1.2 mL). After 45 min of stirring while warming to −35 °C, further DIPEA (24 µL, 0.14 mmol, 1.2 eq) was added. The mixture was warmed to rt and stirred for 19 h. The solvent was removed under reduced pressure and the residue dissolved in EtOAc. The organic layer was washed with H_2O (3x) and brine, dried over $MgSO_4$, filtered and the solvent was removed under reduced pressure. The crude product was

purified by CC (isohexane +1% NEt_3 → isohexane/EtOAc 1:3 + 1% NEt_3) to give product **39** as colourless solid (22 mg, 0.043 mmol, 36%). R_f = 0.46 (isohexane/EtOAc = 2:3). ^{1}H NMR (400 MHz, $CDCl_3$): δ = 7.33 (s, 2H), 7.08 (d, J = 8.6 Hz, 2H), 6.77 (d, J = 8.6 Hz, 2H), 4.80 (t, J = 5.6 Hz, 1H), 4.67 (d, J = 5.6 Hz, 2H), 4.46–4.37 (m, 1H), 3.66 (s, 3H), 3.58–3.38 (m, 3H), 3.38–3.25 (m, 1H), 2.87 (t, J = 7.8 Hz, 2H), 2.59 (t, J = 7.8 Hz, 2H), 2.08–1.57 (m, 6H) ppm. ^{13}C NMR (101 MHz, $CDCl_3$): δ = 173.5, 157.3, 155.9, 136.7, 134.2, 133.7, 133.0, 129.4, 128.6, 116.2, 75.3, 51.7, 46.2, 41.0, 40.4, 36.1, 34.1, 31.7, 30.2, 22.4 ppm. MS (APCI+): *m/z* 512.5 ($[M+H]^+$).

***tert*-Butyl 4-[4-(methoxycarbonyl)phenoxy]azepane-1-carboxylate (42)**.

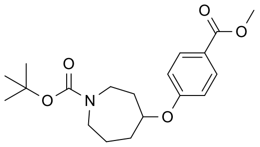

Methyl 4-hydroxybenzoate (0.10 g, 0.65 mmol, 1.0 eq), *tert*-butyl 4-hydroxyazepane-1-carboxylate (0.14 g, 0.65 mmol, 1 eq), and triphenylphosphine (0.36 g, 1.3 mmol, 2.0 eq) were added consecutively to anhydrous THF (8 mL). The resulting solution was degassed with Ar and cooled to 0 °C. This was followed by dropwise addition of diethyl azodicarboxylate (0.23 g, 1.3 mmol, 2.0 eq) in THF (2 mL). The reaction solution was then stirred while warming to rt for 3 days. Subsequently, the solvent was removed under reduced pressure. The crude product was purified by CC (hexane/EtOAC = 90:10 → 50:50) and reversed phase CC (H_2O/MeCN = 87.5:12.5 → 0:100). The product was obtained as a light brownish oil (0.17 g, 0.49 mmol, 76%). t_R: 17.85 min (HPLC method 2), purity: 99% (254 nm). ^{1}H NMR (300 MHz, $CDCl_3$) δ = 8.00–7.93 (m, 2H), 6.90–6.84 (m, 2H), 4.56–4.49 (m, 1H), 3.87 (s, 3H), 3.64–3.23 (m, 4H), 2.14–1.87 (m, 5H), 1.72–1.58 (m, 1H), 1.47 (s, 9H) ppm. MS (ESI+): *m/z* 335.05 ($[M+H]^+$).

Methyl 4-(azepan-4-yloxy)benzoate hydrochlorid salt (43).

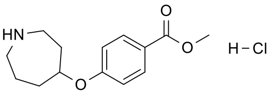

tert-butyl 4-[4-(methoxycarbonyl)phenoxy]azepane-1-carboxylate (**42**, 0.17 g, 0.50 mmol, 1.0 eq) was dissolved in dry MeOH (4 mL) and the solution was cooled to 0°C. Subsequently it was treated with HCl (4M in dioxane, 1 mL, 4 mmol, 8 eq) and stirred over night at rt. The solvent was then partially removed under reduced pressure. **43** was obtained without further purifications as a colourless solid (0.14 g, 0.49 mmol, 98%). t_R: 9.1 min (HPLC method 2), purity: 99% (254 nm). ^{1}H NMR (300 MHz, $CDCl_3$) δ = 9.69 (bs, 2H), 7.98 (d, J = 8.5 Hz, 2H), 6.91 (d, J = 8.6 Hz, 2H), 4.89–4,78 (m, 1H), 3.88 (s, 3H), 3.48b–3.32 (m, 4H), 2.48–1.63 (m, 6H) ppm. MS (ESI+): *m/z* 250.10 ($[M+H\text{-}Cl]^+$).

Methyl 4-({1-[(2,4-dichlorobenzyl)carbamoyl]azepan-4-yl}oxy)benzoate (45).

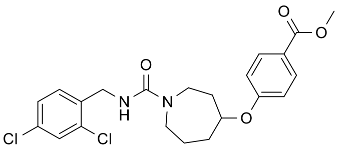

DIPEA (172 µL, 0.98 mmol, 4.0 eq) was added to methyl 4-(azepan-4-yloxy)benzoate hydrochlorid (**43**, 70 mg, 0.24 mmol, 1.0 eq) in anhydrous DCM (3 mL) and the obtained solution was degassed with Ar for several minutes and cooled to 0 °C. Then a solution of triphosgene (30 mg, 0.10 mol, 0.4 eq) in DCM (2 mL) was added dropwise. The reaction mixture was stirred for 2 h while warming to rt. Then 2,4-dichlorbenzylamine (45 mg, 0.33 mmol, 1.0 eq) in DCM (1 mL) was added at 0 °C and the resulting mixture was stirred for 18 h at rt. After addition of DCM the organic layer was washed with brine (1x), dried over $MgSO_4$, filtered, and then the solvent was removed under reduced pressure. The crude product was purified by CC (hexane/EtOAc = 70:30 → 0:100). **45** was obtained as a colourless solid (35 mg, 0.078 mmol, 32%). ^{1}H NMR (300 MHz, MeOD-d_4) δ= 7.98–7.92 (m, 2H), 7.42 (d, J

= 2.0 Hz, 1H), 7.37–7.25 (m, 2H), 7.00–6.93 (m, 2H), 6.90 (t, *J* = 5.9 Hz, 0.7H), 4.67 (tt, *J* = 7.1, 3.4 Hz, 1H), 4.44 (d, *J* = 5.8 Hz, 2H), 3.86 (s, 3H), 3.64–3.44 (m, 4H), 2.18–1.71 (m, 6H) ppm. MS (ESI+): *m/z* 451.10 ([M+H]$^+$).

Methyl (*Z*)-4-[1-(2-Tosylhydrazineylidene)propyl]benzoate (48).

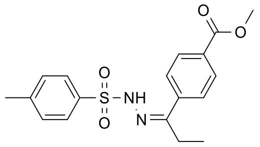

To a solution of 1-(*p*-cabomethoxyphenyl)-2-propanone (250 mg, 1.30 mmol, 1.0 eq) in anhydrous MeOH (3 mL) was added *p*-tolouenesulfonyl hydrazide (250 mg, 1.30 mmol, 1.0 eq) in a sealable vessel. The mixture was degassed, sealed and stirred overnight at 60 °C. The reaction mixture was concentrated under reduced pressure and the remaining residue was extracted with EtOAc. The organic layer was washed with saturated $NaHCO_3$ solution (1x15 mL) and brine (1x15 mL). The organic phase was dried over $MgSO_4$, filtered and the solvent was removed under reduced pressure. The crude product was purified by CC (hexane/EtOAc) to obtain **48** as colourless crystals (378 mg, 1.05 mmol, 81%). ^{1}H NMR (400 MHz, $CDCl_3$): δ = 8.15 (s, 1H), 8.00 (d, *J* = 8.4 Hz, 2H), 7.91 (d, *J* = 7.9 Hz, 2H), 7.70 (d, *J* = 8.4 Hz, 2H), 7.33 (d, *J* = 8.2 Hz, 2H), 3.95–3.92 (m, 3H), 2.61 (dd, *J* = 7.7 Hz, 2H), 2.42 (s, 3H), 1.10 (t, *J* = 7.7 Hz, 3H) ppm. MS (ESI+): *m/z* 360.90 ([M+H]$^+$).

Methyl 4-[1-(3-aminophenyl)propyl]benzoate (50).

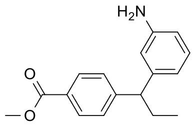

K_2CO_3 (333 mg, 2.38 mmol, 1.5 eq) was added to a solution of **48** (573 mg, 1.59 mmol, 1.0 eq) in 1,4-dioxane (6 mL) and degassed. After adding 3-aminophenylboronic acid monohydrate (370 mg, 2.38 mmol, 1.0 eq) the reaction mixture was over night at 110 °C. After cooling to rt the reaction mixture was extracted with EtOAc. The combined organic layers were washed with saturated $NaHCO_3$ solution (2x15 mL) and brine (1x), dried over $MgSO_4$, filtered and the solvent was removed under reduced pressure.The crude product was purified by CC (hexane/EtOAc) to obtain **50** as colourless crystals (0.10 g, 0.39 mmol, 24%). ^{1}H-NMR (400 MHz, $CDCl_3$): δ = 7.95 (d, *J* = 8.4 Hz, 2H), 7.30 (d, *J* = 8.2 Hz, 2H), 7.07 (m, 1H), 6.65 (d, *J* = 7.7 Hz, 1H), 6.53 (m, 2H), 3.88 (s, 3H), 3.75 (t, *J* = 7.8 Hz, 1H), 2.10–1.99 (m, 2H), 0.90 (t, *J* = 7.3 Hz, 3H) ppm. MS (ESI+): *m/z* 270.10 ([M+H]$^+$).

Methyl 4-{1-[3-({[4-(trifluoromethoxy)phenyl]carbamoyl}amino)phenyl]propyl}benzoate (52).

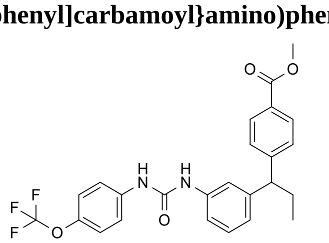

A mixture of **50** (0.20 g, 0.74 mmol, 1 eq) and 4-(trifluoromethoxy)phenylisocanat (0.16 g, 0.74 mmol, 1.0 eq) in THF (5 mL) was stirred over night. The reaction mixture was extracted using EtOAc and the combined organic layers were dried over $MgSO_4$, filtered and the solvent was removed under reduced pressure. The crude product was purified by CC (hexane/EtOAc) to obtain **50** as off-white crystals (0.23 g, 0.49 mmol, 66%). ^{1}H-NMR (300 MHz, DMSO-d_6): δ = 8.81 (s, 1H), 8.65 (s, 1H), 7.89 (d, *J* = 8.3 Hz, 2H), 7.57–7.51 (m, 2H), 7.43 (d, *J* = 8.3 Hz, 2H), 7.37 (s, 1H), 7.30–7.25 (m, 3H), 7.20 (t, *J* = 7.6 Hz, 1H), 6.93 (d, *J* = 7.5 Hz, 1H), 3.89 (t, *J* = 7.8 Hz, 1H), 3.82 (s, 3H), 2.04 (quint, *J* = 7.1 Hz, 2H), 0.83 (t, *J* = 7.2 Hz, 3H) ppm. MS (ESI+): *m/z* 473.00 ([M+H]$^+$).

Methyl 3'-methyl[1,1'-biphenyl]-2-carboxylate (55).

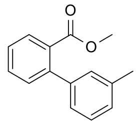

The product was synthesized according to a slightly modified procedure by Pi *et al.*[33] Palladium(II)diacetate (7 mg, 0.03 mmol, 0.05 eq), 1,1′-bis(diphenylphosphino)ferrocene (19 mg, 0.034 mmol, 0.06 eq), K_3PO_4 (358 mg, 1.65 mmol, 3.00 eq), methyl 2-bromobenzoate (0.12 g, 0.55 mmol, 1.0 eq), and *m*-tolylboronic acid (150 mg, 1.10 mmol, 2.00 eq) were added to DME (5 mL) and degassed for several minutes with Ar. Afterwards the reaction mixture was heated overnight at 80 °C. After cooling to rt, H_2O (10 mL) was added and the aqueous phase was extracted with EtOAc (2x). The combined organic phases were washed with brine (1x), dried over $MgSO_4$ and filtered. After evaporation of the solvent, the remaining residue was purified by reversed phase CC (H_2O/MeCN = 12.5:87.5 → 90:10) , followed by an additional washing step with H_2O. **55** was obtained as a colorless oil (114 mg, 0.504 mmol, 91%). R_f: 0.30 (1:1, MeCN/H_2O). t_R: 16.99 min (HPLC method 2), purity: 99% (254 nm). ^1H NMR (250 MHz, DMSO-d_6): δ = 7.71 (dd, J = 7.6, 0.6 Hz, 1H), 7.65–7.57 (m, 1H), 7.52–7.46 (m, 1H), 7.45–7.40 (m, 1H), 7.30 (t, J = 7.5 Hz, 1H), 7.21–7.15 (m, 1H), 7.13–7.03 (m, 2H), 3.58 (d, J = 0.6 Hz, 3H), 2.35 (s, 3H) ppm. MS (ESI): Product does not fly.

Methyl 3'-bromomethyl[1,1'-biphenyl]-2-carboxylate (56).

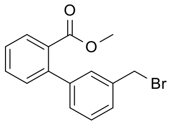

The product was synthesized according to a slightly modified procedure by Gillig *et al.*[34] AIBN (8 mg, 0.05 mmol, 0.1 eq) was added to a solution of methyl 3'-methyl[1,1'-biphenyl]-2-carboxylate (**55**, 114 mg, 0.504 mmol, 1.0 eq) in $CHCl_3$ (10 mL), the solution was degassed with Ar for several minutes and then heated to 50 °C. NBS (135 mg, 0.758 mmol, 1.5 eq) was subsequently added and the reaction mixture was heated to 76 °C overnight. After cooling to rt, H_2O was added and the aqueous phase was extracted with DCM (2x). The combined organic phases were washed with brine (1x), dried over $MgSO_4$, filtered and the solvent was removed under reduced pressure. The remaining residue was further purified by CC (hexane/EtOAc = 99:1). **56** (118 mg, 0.388 mmol, 77%) was obtained as a mixture with the dibrominated species. The mixture was used in the next step without further purification. R_f: 0.17 (hexane/EtOAc = 98:2). t_R: 17.07 min (HPLC method 2), purity: 78% (254 nm). ^1H NMR (250 MHz, DMSO-d_6): δ = 7.76 (dd, J = 7.7, 1.5 Hz, 1H), 7.65 (td, J = 7.5, 1.5 Hz, 1H), 7.53 (dd, J = 7.5, 1.4 Hz, 1H), 7.47–7.41 (m, 2H), 7.36 (d, J = 1.5 Hz, 2H), 7.26 (dt, J = 6.6, 2.0 Hz, 1H), 4.76 (s, 2H), 3.60 (s, 3H) ppm. MS (ESI): Product does not fly.

Methyl 3'-{[4-(1-methoxy-1-oxohex-4-yn-3-yl)phenoxy]methyl}[1,1'-biphenyl]-2-carboxylate (58).

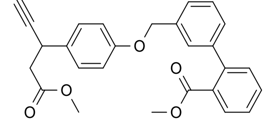

The product was synthesized according to a slightly modified procedure by Gagnon *et al.*[35] Methyl 3'-(bromomethyl)[1,1'-biphenyl]-2-carboxylate (**56**, 118 mg, 0.388 mmol, 0.95 eq, purity: 78%,) and methyl 3-(4-hydroxyphenyl)hex-4-ynoate (74 mg, 0.32 mmol, 1.0 eq) were dissolved in acetone (4 mL) followed by the addition of Cs_2CO_3 (105 mg, 0.322 mmol, 1.0 eq). After stirring overnight at rt, the reaction mixture was filtered, the filter cake was washed with further acetone and the filtrate was evaporated under reduced pressure. The crude product was purified by CC (hexane/EtOAC = 98.8:1.2 → 75:25). **58** was obtained as a slightly yellowish oil (105 mg, 0.237 mmol, 78%). R_f: 0.13 (hexane/EtOAc = 95:5). t_R: 17.99 min (HPLC method 2), purity: 99% (254 nm). ^1H NMR (250 MHz, DMSO-d_6): δ = 7.74 (dd, J = 7.6, 1.5 Hz, 1H), 7.63 (td, J = 7.5, 1.5 Hz, 1H), 7.51 (dd, J =

7.5, 1.4 Hz, 1H), 7.48–7.42 (m, 3H), 7.35 (s, 1H), 7.31–7.23 (m, 3H), 6.97 (d, *J* = 8.7 Hz, 2H), 5.13 (s, 2H), 3.98 (td, *J* = 7.7, 2.6 Hz, 1H), 3.55 (d, *J* = 5.5 Hz, 6H), 2.70 (d, *J* = 7.7 Hz, 2H), 1.77 (d, *J* = 2.4 Hz, 3H) ppm. MS (ESI+): *m/z* 465.38 ($[M+Na]^+$).

Supplementary References

1. Bickerton, G. R., Paolini, G. V., Besnard, J., Muresan, S. & Hopkins, A. L. Quantifying the chemical beauty of drugs. *Nat. Chem.* **4**, 90–98 (2012).

2. Daina, A., Michielin, O. & Zoete, V. SwissADME: a free web tool to evaluate pharmacokinetics, drug-likeness and medicinal chemistry friendliness of small molecules. *Sci. Reports 2017 71* **7**, 1–13 (2017).

3. Keiser, M. J. *et al.* Relating protein pharmacology by ligand chemistry. *Nat. Biotechnol.* **25**, 197–206 (2007).

4. Raymond, J. W. RASCAL: Calculation of Graph Similarity using Maximum Common Edge Subgraphs. *Comput. J.* **45**, 631–644 (2002).

5. Wang, N., Zou, Q., Xu, J., Zhang, J. & Liu, J. Ligand binding and heterodimerization with retinoid X receptor α (RXRα) induce farnesoid X receptor (FXR) conformational changes affecting coactivator binding. *J. Biol. Chem.* **293**, 18180–18191 (2018).

6. Akwabi-Ameyaw, A. *et al.* Conformationally constrained farnesoid X receptor (FXR) agonists: Naphthoic acid-based analogs of GW 4064. *Bioorg. Med. Chem. Lett.* **18**, 4339–4343 (2008).

7. Flatt, B. *et al.* Discovery of XL335 (WAY-362450), a highly potent, selective, and orally active agonist of the farnesoid X receptor (FXR). *J. Med. Chem.* **52**, 904–907 (2009).

8. Ye, L. *et al.* Thyroid receptor ligands. 1. Agonist ligands selective for the thyroid receptor β1. *J. Med. Chem.* **46**, 1580–1588 (2003).

9. Hangeland, J. J. *et al.* Thyroid receptor ligands. Part 2: Thyromimetics with improved selectivity for the thyroid hormone receptor beta. *Bioorg. Med. Chem. Lett.* **14**, 3549–3553 (2004).

10. Yao, B. *et al.* Revealing a Mutant-Induced Receptor Allosteric Mechanism for the Thyroid Hormone Resistance. *iScience* **20**, 489–496 (2019).

11. Batista, F. A. H. *et al.* Structural Insights into Human Peroxisome Proliferator Activated Receptor Delta (PPAR-Delta) Selective Ligand Binding. *PLoS One* **7**, e33643 (2012).

12. Wu, C. C. *et al.* Structural basis for specific ligation of the peroxisome proliferator-activated receptor δ. *Proc. Natl. Acad. Sci. U. S. A.* **114**, E2563–E2570 (2017).

13. Öster, L., Tapani, S., Xue, Y. & Käck, H. Successful generation of structural information for fragment-based drug discovery. *Drug Discov. Today* **20**, 1104–1111 (2015).

14. Tanaka, D. *et al.* A practical use of ligand efficiency indices out of the fragment-based approach: Ligand efficiency-guided lead identification of soluble epoxide hydrolase inhibitors. *J. Med. Chem.* **54**, 851–857 (2011).

15. Amano, Y., Yamaguchi, T. & Tanabe, E. Structural insights into binding of inhibitors to soluble epoxide hydrolase gained by fragment screening and X-ray crystallography. *Bioorg. Med. Chem.* **22**, 2427–2434 (2014).

16. Gilson, M. K. *et al.* BindingDB in 2015: A public database for medicinal chemistry, computational chemistry and systems pharmacology. *Nucleic Acids Res.* **44**, D1045–D1053 (2016).

17. Davies, M. *et al.* ChEMBL web services: streamlining access to drug discovery data and utilities. *Nucleic Acids Res.* **43**, W612 (2015).

18. Daina, A., Michielin, O. & Zoete, V. SwissADME: A free web tool to evaluate pharmacokinetics, drug-likeness and medicinal chemistry friendliness of small molecules. *Sci. Rep.* **7**, (2017).

19. Schierle, S. *et al.* Design and Structural Optimization of Dual FXR/PPARδActivators. *J. Med. Chem.* **63**, 8369–8379 (2020).

20. Heitel, P. *et al.* Computer-Assisted Discovery and Structural Optimization of a Novel Retinoid X Receptor Agonist Chemotype. *ACS Med. Chem. Lett.* **10**, 203–208 (2019).

21. Schierle, S. *et al.* Design and Structural Optimization of Dual FXR/PPARδActivators. *J. Med. Chem.* **63**, 8369–8379 (2020).

22. Wuts, P. G. M. *Greene's Protective Groups in Organic Synthesis*. (Wiley, 2014).

23. Blöcher, R. *et al.* N-Benzylbenzamides: A Novel Merged Scaffold for Orally Available Dual Soluble Epoxide Hydrolase/Peroxisome Proliferator-Activated Receptor γ Modulators. *J. Med. Chem.* **59**, 61–81 (2016).

24. Spurg, A. & Waldvogel, S. R. High-Yielding Cleavage of (Aryloxy)acetates. *European J. Org. Chem.* **2008**, 337–342 (2008).

25. Kareem, H. S. *et al.* Correlation of antioxidant activities with theoretical studies for new hydrazone compounds bearing a 3,4,5-trimethoxy benzyl moiety. *Eur. J. Med. Chem.* **103**, 497–505 (2015).

26. Amundsen, L. H. & Nelson, L. S. Reduction of Nitriles to Primary Amines with Lithium Aluminum Hydride. *J. Am. Chem. Soc.* **73**, 242–244 (1951).

27. Wuts, P. G. M. *Greene's Protective Groups in Organic Synthesis*. (Wiley, 2014).

28. Wu, J., Bär, R. M., Guo, L., Noble, A. & Aggarwal, V. K. Photoinduced Deoxygenative Borylations of Aliphatic Alcohols. *Angew. Chemie Int. Ed.* **58**, 18830–18834 (2019).

29. Cottrell, K. M., Maxwell, J. P. & Whittington, D. A. Preparation of substituted pyrimidinecarboxamides, pyridinecarboxamides and benzamides as PRMT5 inhibitors. (2021).

30. Pinte, J., Joly, C., Plé, K., Dole, P. & Feigenbaum, A. Proposal of a set of model polymer additives designed for confocal FRAP diffusion experiments. *J. Agric. Food Chem.* **56**, 10003–10011 (2008).

31. Ebdrup, S. & Andersen, H. S. 11β-hydroxysteroid dehydrogenase type 1 active compounds. (2011).

32. Gastaldi, S., Weinreb, S. M. & Stien, D. Diiodosilane: A reagent for mild, efficient conversion of carbamates to ureas via isocyanates. *J. Org. Chem.* **65**, 3239–3240 (2000).

33. Pi, J. J. *et al.* Exploration of Biaryl Carboxylic Acids as Proton Shuttles for the Selective Functionalization of Indole C-H Bonds. *J. Org. Chem.* **83**, 5791–5800 (2018).

34. Gillig, J. R. *et al.* Novel MCH Receptor Antagonists. (2003).

35. Gagnon, L., Lagraoui, M. & Grouix, B. Substituted phenylpropionic acids as stimulators of hematopoiesis and erythropoiesis. (2008).
